# Atomic Building Blocks of Global Buildings

**DOI:** 10.1021/acs.est.5c11079

**Published:** 2026-03-10

**Authors:** Jonathan M. Broyles, Matt A. Jungclaus, Danielle N. Beatty, Wil V. Srubar III

**Affiliations:** † Department of Civil, Environmental, and Architectural Engineering, 1877 University of Colorado Boulder, Boulder, Colorado 80309, United States; ‡ Materials Science and Engineering Program, University of Colorado Boulder, Boulder, Colorado 80303, United States

**Keywords:** Building materials, material use intensity, atomic use intensity, global building stock, circular economy, urban mining

## Abstract

Buildings require vast quantities of materials and natural resources. Quantifying and understanding the composition of whole buildings is necessary to support circular economies that mitigate the future environmental impacts of the built environment. An understanding of the chemical composition of buildings can support urban mining efforts for resource management and elemental recovery in addition to furthering the knowledge of the carbon storage potential of buildings. This study estimated the material use intensities (MUIs) (kg/m^2^) and atomic use intensities (AUIs) (mol/m^2^) of 1028 whole buildings across eight global regions. Results reveal that buildings primarily consist of six atomic elements (*i*.*e*., oxygen, calcium, silicon, carbon, iron, and aluminum). Collectively, these six elements comprise ∼97% of the mass of buildings worldwide. Our analysis also reveals that the average AUIs of whole buildings remain relatively constant and do not vary by global region or building typology. Together, the methodology and data presented herein offer valuable insights for advancing urban mining and circular economy strategies for the global construction sector.

## Graphical abstract

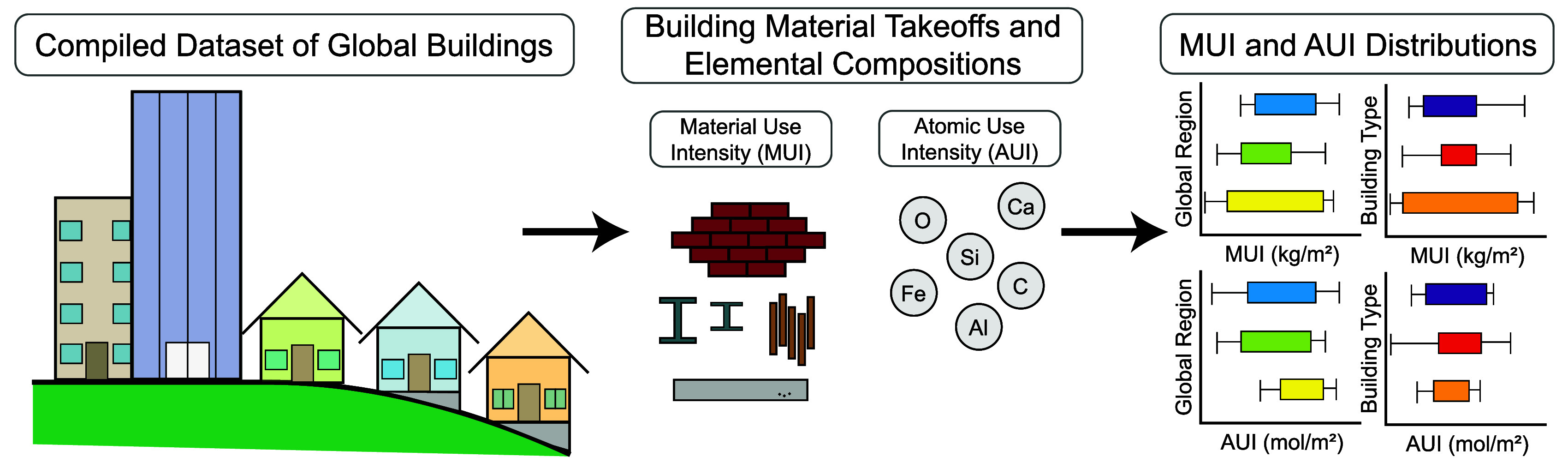

## Introduction

The construction of buildings requires more than 17 billion tonnes of material on an annual basis, which is equivalent to 24% of all raw material extracted from the lithosphere each year.^1,2^ Furthermore, the sector accounts for 23% of global CO_2_ emissions,^3^ which are responsible for global warming and climate change. The demand for new buildings is expected to increase significantly over the next century, especially in developing countries.^4,5^ By 2050, global floor space is projected to increase by 75%.^6,7^ Consequently, implementing sustainable solutions that reduce construction material consumption and emissions, while addressing the growing demand for materials, is necessary to effectively mitigate climate change, improve construction material supply chains, and aid recyclability practices.

Establishing circular economies (CEs) for building materials is an emerging sustainable building strategy. CEs have been shown to reduce the embodied carbon emissions, namely the emissions associated with the manufacture, transport, use, and disposal of building materials, up to 80% through urban mining (*i*.*e*., direct reuse of reclaimed building materials).^8–11^ CEs of structural materials (*i*.*e*., concrete or steel) would be particularly effective in reducing embodied carbon emissions of new buildings.^12^ Concrete accounts for approximately 30–40% of global material consumption by unit mass^2,13,14^ and more than 8% of global CO_2_ emissions^15^ across all sectors. Steel represents less than 5% of global material use^2^ by unit mass but is responsible for 7–9% of global CO_2_ emissions.^16^ Like building floor space, global production of concrete and steel is projected to increase through 2050 by 12–23% and 33%, respectively, from current production levels.^17,18^ Therefore, adopting sustainable material solutions, particularly those facilitated by CE frameworks, can directly address construction material resource availability, recyclability, and as a result, climate change.

While the potential environmental benefits of CEs for building materials are evident, studies that elucidate the material composition of whole buildings can inform current and future building material supply chains.^19^ Several researchers have studied the material composition of whole buildings, noting that the built environment consumes a significant amount of material.^20–25^ Miatto et al.^23^ found that concrete was the most utilized material across all building typologies by mass. However, Gonita et al.^21^ found that alternative construction materials, like steel and wood, had the largest material intensity for residential buildings in Sweden, suggesting that geographical region affects the materiality of a building. A comprehensive understanding of building material compositions also informs whole-building life cycle assessments (wbLCAs), including embodied carbon emissions, and the carbon storage potential in the built environment.^9,12,26–31^

While the material composition of buildings is, and should continue to be, further studied to catalyze CEs for building materials in different global regions, knowledge of the chemical composition of buildings can complement CEs by better supporting atomic element supply chains through elemental recovery. In recent years, the availability of certain atomic elements has become an increasingly important topic in certain industries, particularly concerning rare earth metals, high-quality limestone for cement production, and CO_2_ mineralization using free calcium leached from concrete.^32–37^ Many construction material and demolition urban mining studies focus on the high concentrations of metals in buildings, including iron, aluminum, and copper.^38–44^ Schäfer and Schmidt^42^ found that while buildings have lower concentrations of metal elements compared to primary sources (*i*.*e*., natural ores), the metals are available in larger quantities, which could be used to support the atomic elemental demand in other industries, like electronics.^45^ To help inform elemental supply chains, Koutamanis et al.^43^ determined approximate quantities of metals in residential buildings, with steel (0.1–8.6 kg/m^3^), aluminum (0.03–0.5 kg/m^3^), and copper (0.002–0.5 kg/m^3^) being the most prevalent on a per-volume basis. Zeng and Li^44^ broadly reviewed how CEs, including the underlying chemistry of building materials and processes, can provide a more comprehensive understanding of environmental performance and material recycling.

While these efforts demonstrate that urban mining of buildings can support CEs of building materials and atomic elemental supply chains, much of the material composition research is constrained to a specific geographical region and the atomic composition research is limited to metal elements. Therefore, there is a gap in knowledge on the material and atomic compositions of whole buildings at a local, regional, or global scale. Yet, as motivated by previous research, a better understanding of the material and chemical composition of buildings can inform global building material CE frameworks, catalyze elemental recyclability efforts, and improve carbon storage efforts in buildings. Such an analysis would provide valuable insights into the distribution and availability of materials within the built environment, enabling more effective strategies for resource management, material recovery, and minimizing environmental impacts.

In response, this study presents the first comprehensive material use intensity (MUI) (kg/m^2^) and atomic use intensity (AUI) (mol/m^2^) analysis of 1028 global buildings that varied by region and typology. First, the MUIs for the buildings were estimated. Then, the elemental composition of the entire building was determined using the MUIs along with industry composition standards and existing literature. Lastly, the AUIs for the global building stock were defined for six of the most prevalent atomic elements in buildings. Quantifying the AUIs of buildings can complement MUI results by revealing what materials, global regions, and building typologies could be viable sources for elemental recycling and other urban mining strategies. Overall, this paper contributes to the growing body of literature that seeks to understand the underlying material and atomic composition of the built environment to better support the establishment of CEs for buildings.

## Materials and Methods

### Material Composition of Global Buildings

The material quantities of 1028 buildings were collected from a combination of (1) material quantity and life cycle assessment (LCA) studies and (2) publicly available bill of material (BOM) whole-building databases. The building component scope of all material quantity takeoffs included the building structure and envelope. Additionally, interior partitions and finishes were only included in the building component scope if those materials were reported in the data set. The material intensity of buildings data set by Heeren and Fishman contributes most of the building material data.^46–48^ The confidence of this particular data set is high, given its rigorous approach to data harmonization and validation. The Heeren and Fishman data set is also open-source and community-driven, suggesting that errors have been flagged and corrected.^46^ Whole-building characteristics, including typologies and geographies, are summarized in Table 1 and shown in Figure 1. For more details see Supporting Information Table S-1.

**Figure 1:**
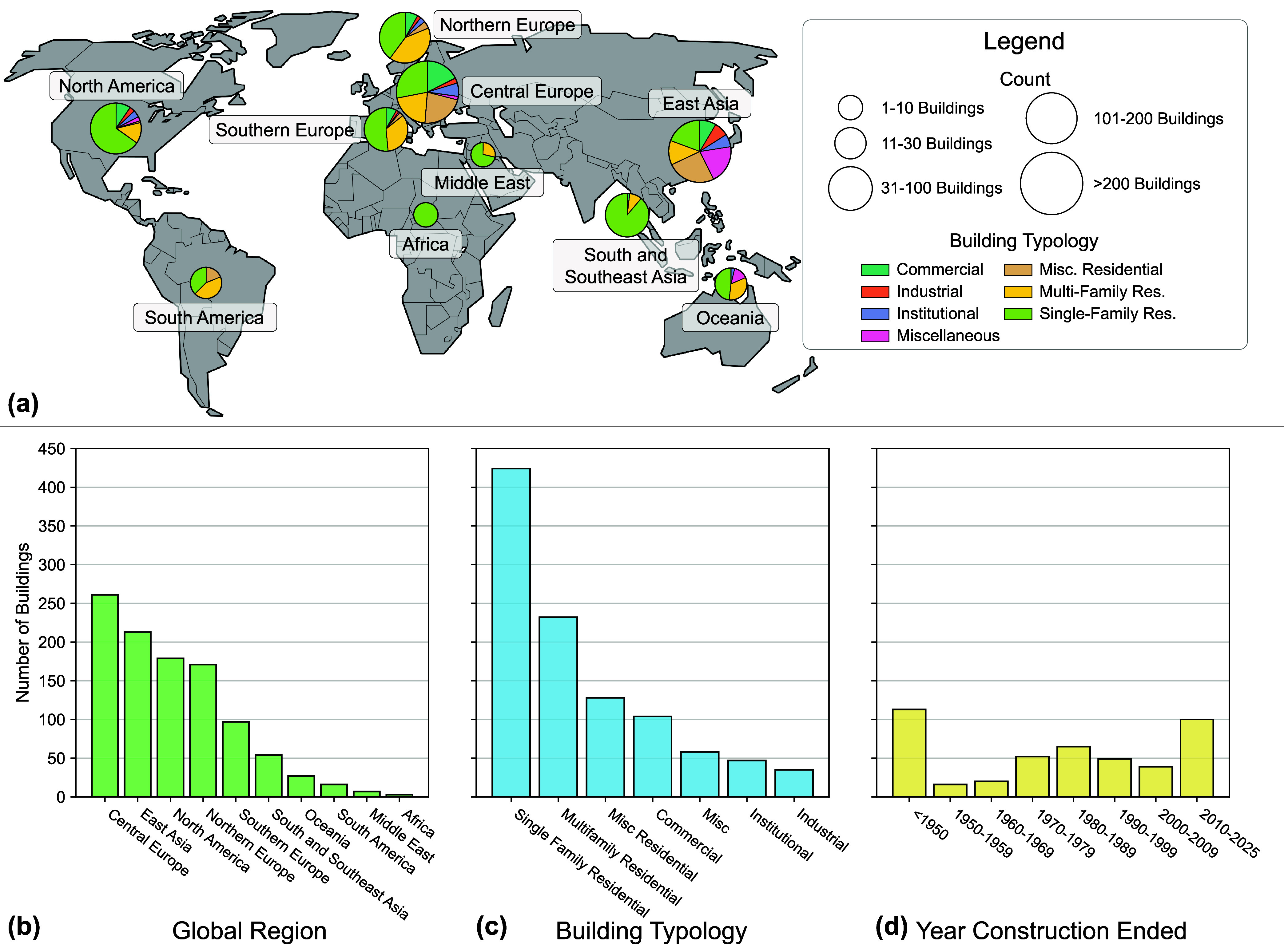
Geospatial distribution of the buildings included in this study. (a) The size and distribution of the pie charts symbolizes the number and typology of buildings represented in each global region, respectively. (b–d) The distribution of the buildings in the data set grouped by global region, building typology, and construction year.

**Table 1:** Summary of Buildings Included in This Study

No. Buildings	Building Typologies	Global Regions	Source
6	Commercial	North America	Athena Sustainable Materials Institute Publications webpage^49^
	Industrial		
	Institutional		
	Multifamily residential		
572^a^	Commercial	Africa	*A database seed for a community-driven material intensity research platform* ^46,47^
	Industrial	Asia	
	Institutional	Northern Europe	
	Misc.	Central Europe	
	Misc. residential	Southern Europe	
	Multifamily residential	North America	
	Single-family residential	Oceania	
		South America	
380	Commercial	Africa	*A database seed for a community-driven material intensity research platform* ^48^ ^b^
	Industrial	Asia	
	Institutional	Northern Europe	
	Misc.	Central Europe	
	Misc. residential	Southern Europe	
	Multifamily residential	North America	
	Single-family residential	Oceania	
		South America	
70	Commercial	North America	*A construction classification system database for understanding resource use in building construction* ^50,51^
	Institutional		
	Multifamily residential		
	Single-family residential		

a 577 buildings existed in the original database at the time of this research. Five buildings were excluded due to anomalous material quantities.

b The original data set provided by Heeren and Fishman was updated to include data provided in other published studies.^24,52–81^

Figure 1 shows that most buildings (89.6%) included in this study are located in Europe, East Asia, and North America. To better contextualize the results of the European buildings, three additional regions were defined: Northern Europe (Norway, Sweden, Finland, Lithuania, Latvia, Estonia, and Iceland), Central Europe (Austria, Belgium, Czech Republic, Denmark, Germany, Hungary, Ireland, Luxembourg, The Netherlands, Poland, Scotland, Slovakia, Switzerland, and the United Kingdom), and Southern Europe (Cyprus, France, Greece, Italy, Malta, Portugal, Spain). Africa, the Middle East, and South America are underrepresented in the data set (2.5% of buildings combined). Regarding building typology, single family residential buildings are overrepresented (41.2%) while industrial and institutional buildings are underrepresented (8.0% of buildings combined). Global regions with larger representation in the data set typically included multiple building typologies, while data for other global regions were primarily attributable to single family and multifamily residential homes. Lastly, Figure 1d shows that the data set included older and newer buildings; however, 578 of the buildings (56.2%) did not provide construction year information.

The building databases reported either a material use intensity (MUI) (kg/m^2^) for each material in each building or a material quantity of each material in various units, such as mass, material surface area, and volume. These latter values were converted as necessary to mass (kg) using relevant material properties (*e*.*g*., density), and dividing by gross floor area (m^2^), resulting in consistent MUI units of kg/m^2^ for all materials in all buildings. For the MUIs, see Supporting Information Table S-1.

### Atomic Use Intensity (AUI) of Global Buildings

The elemental composition and the atomic use intensity (AUI) of global buildings was determined using the calculated MUIs and a combination of chemical element standards and existing literature. NIST Standard Reference Materials (SRMs),^82^ which list the elemental composition of common materials, were used directly when possible. The NIST SRMs are rigorously characterized compositional reference materials with representative chemistries, ensuring applicability to materials derived from both U.S. and non-U.S. sources.^83^ When statistical distributions of material compositions were reported, only the average (mean) value was used for calculating elemental composition. For materials not listed in a NIST SRM, ASTM standards,^84^ which often list material composition limits, were used when possible. Maximum composition limits were assumed for all elements reported. For materials not listed in a NIST SRM or ASTM standard, environmental product declarations (EPDs) were utilized.^85^ Polymer databases (*e*.*g*., PubChem) were used for all polymer elemental compositions.^86^ For building materials not found in the above resources, scientific research papers that reported elemental compositions were used. For other building materials, material safety data sheets, LCA studies, and reports written by national and international organizations (*e*.*g*., NIST, the UN Environmental Council, and the World Health Organization) were used. A full list of elemental compositions and sources by material category are provided in Supporting Information Tables S-2–S-4.

The elemental composition of a building was calculated by weighing the elemental composition of each building material by the overall weight of that material in each building

1 $$c_{x,u}=\frac{{\mathrm{MUI}}_{i,u}\times c_{x,i}}{{\mathrm{MUI}}_{u}}$$

where *c*_*x,u*_ is the percent composition of the element (%), *x*, in building *u*, and MUI_*i,u*_ is the MUI (kg/m^2^) of material, *i*, in building, *u*. *c*_*x,i*_ is the percent composition of element, *x*, in material *i*, and MUI_*u*_ is the total MUI of building, *u*. Then, the AUI for each building (mol/m^2^), AUI_*u*_, was determined by summing the product of the MUIs for each material, *i*, for all materials, *n*, by the percent composition of each chemical element, *x*, in material, *i*, in building *u*, the atomic mass for a chemical element

2 $$\mathrm{AU}I_{u}=\sum_{i=1}^{m}\sum_{x=1}^{n}{\mathrm{MUI}}_{i,u}\times c_{x,i}\times \left(\frac{1}{\mathrm{Atomic}\;\mathrm{Mass}}\right)$$

where *m* is the total number of materials in a building, *u*, and *n* is the total number of elements in a material, *i*. The atomic masses used in the AUI calculation for the six most prevalent elements included in this study are provided in Table 2.

**Table 2:** Atomic Masses of the Six Most Prevalent Chemical Elements Used in the AUI Calculation

Chemical Element	Atomic Mass (g/mol)
Oxygen	15.99
Calcium	40.08
Silicon	28.09
Carbon	12.01
Iron	55.85
Aluminum	26.98

While this study considers a broad range of construction materials and assumes their most universally agreed upon elemental composition, there are three sources of uncertainty. First, regional differences (even within the same global region) result in different elemental compositions due to varying building compositions.^87^ Second, differences in material composition (e.g., concrete mixtures) and manufacturing (e.g., composite materials) may have different elemental compositions. Similarly, different species (e.g., lumber) may have subtle differences in elemental composition. Because of these sources of uncertainty, the AUI results should be interpreted as estimates. However, additional studies should be conducted to refine the findings, especially for global regions with fewer building data, using the methodology presented herein.

### Statistical Analysis

To further understand the similarities and differences in MUI and AUI between global regions and between building typologies, two-sample Kolmogorov–Smirnov (KS) tests were performed. KS tests evaluate the statistical significance between two independent samples,^89^ thus statistical significance implies that the MUI or AUI distributions between pairs of global regions or pairs of building typologies are statistically different. Statistically different MUIs and AUIs can thus confirm that different quantities of building materials or elements can be recovered and recycled, depending on the global region and building typology. Additionally, the Spearman correlation coefficient (*r*_*s*_) was found using Ydata profiling to assess how other building features (*i*.*e*., floor area) correlated with MUI and AUI.^88^

## Results and Discussion

### MUIs Vary by Global Region and Building Typology

Figure 2 shows the MUIs for all 1028 buildings by (a) global region and (c) building typology. The median MUI and IQR for all buildings analyzed herein is 1082 kg/m^2^ of building floor space and 611–1602 kg/m^2^, respectively. The probabilistic distributions of MUIs of seven global regions (*i*.*e*., Southern Europe, East Asia, Central Europe, Oceania, Northern Europe, North America, and South and Southeast Asia) were found to be statistically significant (*i*.*e*., different) from each other and from the distribution of MUIs when grouped by all global regions according to KS tests. When comparing MUIs across global region, the data show that buildings in Southern Europe exhibit the highest median MUI (1413 kg/m^2^), followed by those in East Asia (1388 kg/m^2^) and Central Europe (1323 kg/m^2^). Buildings in South and Southeast Asia exhibit the lowest median MUI (314 kg/m^2^). Some of these differences can be explained by the types of buildings included in each data set. Forty-eight of 54 buildings in South and Southeast Asia are single-family residential homes, which typically have lower MUIs than other building typologies (see Table S-1 in the Supporting Information).^9,90^ Conversely, the data sets for buildings in Central Europe and East Asia include a variety of building typologies, including residential (*i*.*e*., single-, multifamily, and miscellaneous residential) and nonresidential (*i*.*e*., commercial, industrial, and miscellaneous). Despite having different median MUIs, buildings in South and Southeast Asia (21–2039 kg/m^2^) and Oceania (759–2343 kg/m^2^) exhibit the two largest IQRs, whereas North America (517–909 kg/m^2^) and Africa (363–694 kg/m^2^) exhibit the smallest IQRs. While the small IQR for Africa is likely owing to its small sample size (*n* = 3), North America has a large sample size (*n* = 179) but most of the buildings (80%) are residential, which trend with lower MUIs compared to the other building typologies.

**Figure 2:**
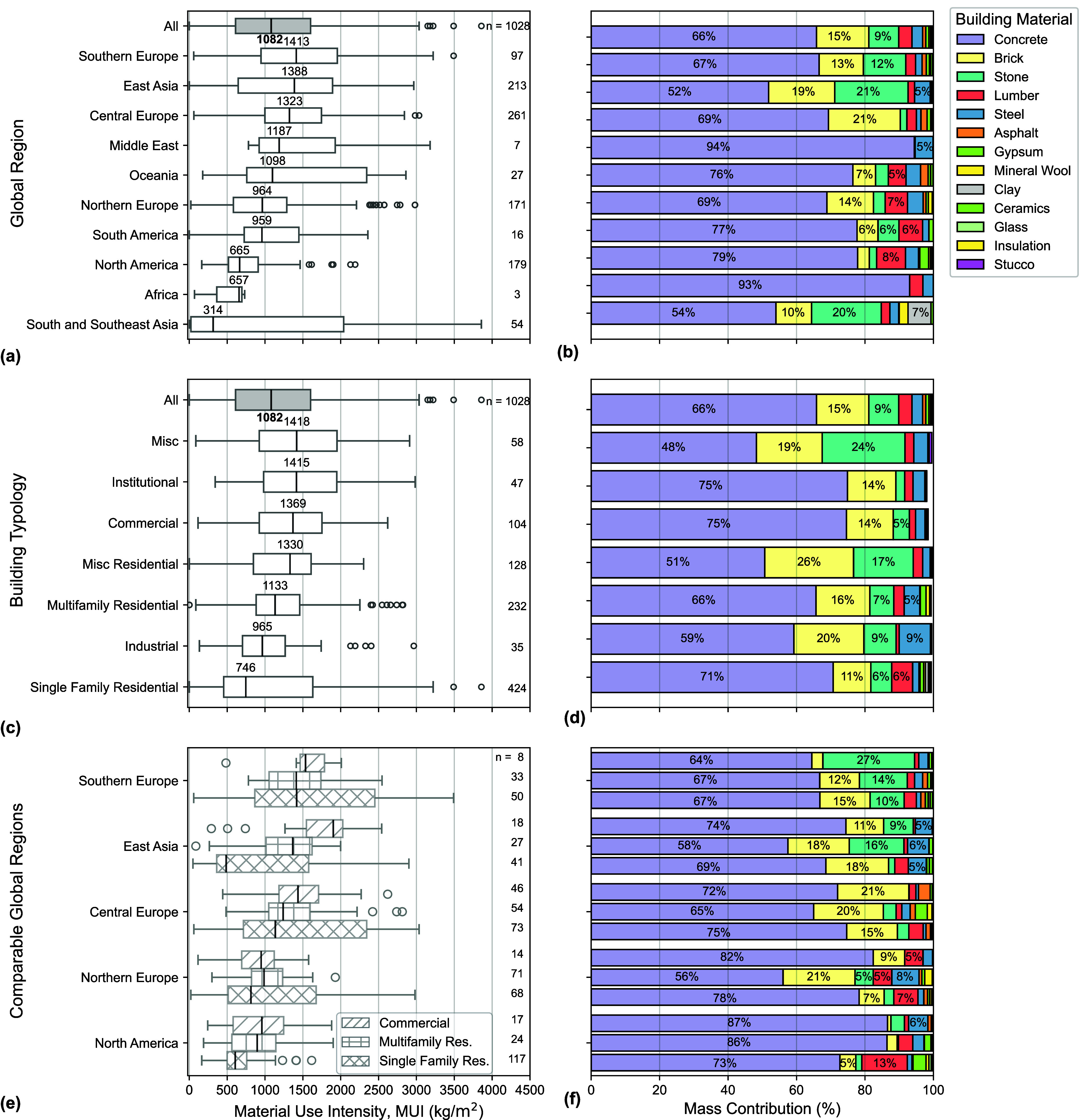
MUIs of global buildings vary by region and typology, but the mass of all buildings is dominated by concrete, brick, and stone. MUIs (kg/m^2^) and relative mass contribution (%) of all buildings classified by (a, b) global region and (c, d) building typology. (e, f) MUIs and relative mass contribution for the three most common building typologies (*i.e.,* commercial, multifamily residential, and single family residential) for the five comparable global regions.

Figure 2b shows the average material contributions (by mass) for all buildings by global region. Thirteen building materials account for >99.5% of MUI. Figure 2b reveals that concrete, which also encompasses cement and mortar in this categorization, is, in general, the most mass-intensive material across all global regions, representing 52–93% of total MUI on average. The second most intensive material varies by global region. Brick is the second largest contributor (7–21%) to the MUI of buildings in Southern Europe, Central Europe, Northern Europe, and Oceania, while stone is the second largest contributor in East Asia and South and Southeast Asia (20–21%). The next largest MUI contributors in the Middle East, Africa, and North America are steel and lumber, but they have much lower percentage contributions (∼6–8%) to total MUI than concrete.

Figure 2c shows the MUI distributions by building typology. Miscellaneous (*e*.*g*., parking garage, public assemblies), institutional, commercial, and miscellaneous residential buildings have median MUIs > 1300 kg/m^2^. Single-family residential buildings have the lowest median MUI, as expected.^9,28,90^ Single-family residential buildings, however, have the widest IQR of MUI (454–1631 kg/m^2^), despite having the lowest median MUI. Miscellaneous (913–1957 kg/m^2^), commercial (924–1752 kg/m^2^), and institutional (975–1968 kg/m^2^) buildings also have wide MUI IQRs between 900 and 2000 kg/m^2^. Miscellaneous residential (847–1609 kg/m^2^), and industrial (689–1328 kg/m^2^) buildings have narrower MUI IQRs, while multifamily buildings had the narrowest IQR (883–1458 kg/m^2^). Due to the high variability in IQR, all MUI distributions by building typology were not statistically similar from the other building typologies when evaluated using KS tests. Additionally, the correlation between gross floor area and MUI was statistically significant (*r*_*s*_ = 0.543), which gives credence to reports in existing literature and structural engineering intuition that low-rise buildings use less material per unit floor area than high-rise buildings.^23,90,91^

Figure 2d shows the average material contributions (by mass) for all buildings by building typology. Concrete contributes more to total mass (48–75%) than any other material for all building typologies. Although single family and multifamily residential buildings are typically composed of lumber in some global regions (e.g., North America), the concrete used in the foundations is significantly more mass-intensive in respect to the other building materials that compose the residential buildings. Jungclaus et al.^28^ also found that concrete was the primary contributor to the MUI and the embodied carbon intensity (kgCO_2_e/m^2^) of single family residential buildings in the United States, indicating that strategies to efficiently reduce concrete in the built environment should be prioritized in new construction.^92–94^ Aside from concrete, brick and stone contribute 11–26% and 3–24% to mass, respectively, whereas steel and lumber contribute up to 9% to the total mass, depending on the typology.

Figure 2e shows that Southern Europe, East Asia, and Central Europe typically have higher MUIs compared to Northern Europe and North America and that commercial buildings have higher MUIs compared to multifamily and single-family residential buildings. However, regional MUI differences across the three building typologies can also be observed. For example, the median MUI of single-family residential buildings in East Asia are lower than any other building type in the other four regions, despite having higher overall MUIs. The MUI of multifamily residential buildings in Northern Europe is also higher than that of commercial buildings, although commercial buildings typically have higher MUIs. The percentage contribution to the mass (Figure 2f) underscores materiality differences. Stone is much more commonly used in Northern European commercial, multifamily residential, and single-family residential buildings compared to the other global regions. Another observation is that steel contributes more to the mass of North American commercial buildings although steel contributes a relatively low percent to the mass of commercial buildings when aggregated across all global regions. These findings highlight how regional design decisions and construction practices influence building material availability, and thus building material CEs, which has also been noted by previous studies.^87,90,95–99^

### 97% of the Global Building Stock Consists of Six Atomic Elements

The elemental composition of the buildings analyzed herein is shown in Figure 3. Figure 3a reveals that oxygen (O) is the most common atomic element by mass (42–56%) for most building materials. The exception is steel, which is composed of 94% iron (Fe). Calcium (Ca) and silicon (Si) are also predominant elements in the composition of common building materials. Glass, for example, contains 3% Ca and 34% Si, whereas stucco contains 36% Ca and 12% Si. Carbon (C) contributes significantly to the composition of lumber (50%), and, to a lesser extent, concrete (8%) and stone (4%).

**Figure 3:**
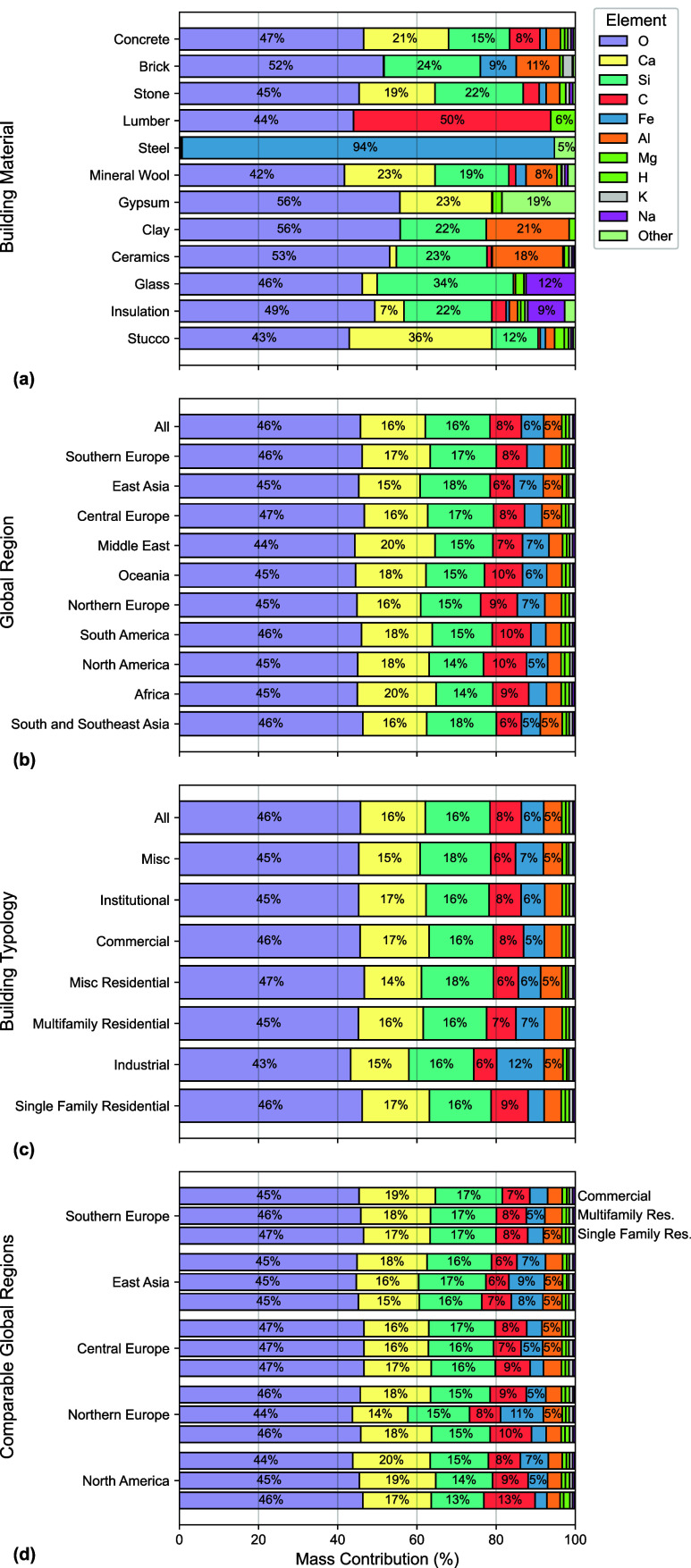
Construction materials and whole buildings are primarily composed of six atomic elements. Average elemental compositions of (a) the top 12 building materials (by mass), (b) all global buildings per global region, (c) all global buildings per building typology, and (d) commercial, multifamily residential, and single family residential buildings for the five comparable global regions.

The elemental compositions of the buildings analyzed in this work are similar irrespective of their classification by global region and building typology (Figure 3b–d). O contributes most to the mass of buildings (43–47%), followed by Ca (14–20%), Si (13–18%), C (6–13%), Fe (3–12%), and Al (3–5%). Together, approximately 97% of the global building stock is comprised of these six elements. Because the mass contributions of concrete, brick, and stone sum to 90% of all building materials (see “All” in Figure 2b,d), the elemental composition across global regions and building typologies is dominated by the elemental composition of these materials. Although lumber and steel have smaller contributions to mass in the buildings analyzed herein compared to concrete, brick, and stone, they are primarily composed of C and Fe, which explains why these elements are the fourth and fifth largest contributors. Like the MUIs, commercial, multifamily residential, and single family residential buildings across the five comparable global regions have differences in the elemental mass contribution-albeit subtle differences. However, multifamily residential buildings in Northern Europe have more Fe and North American single family homes have significantly more C compared to all other building types. These findings indicate that regionality may influence the success of urban mining efforts to recover metals (*i*.*e*., Fe and Al) and the amount of carbon that can be sequestered by building materials.^33,39,100^

The AUIs (mol/m^2^) for the six atomic elements (*i*.*e*., O, Si, Ca, C, Fe, and Al) that primarily comprise the buildings analyzed herein are shown in Figure 4. The median AUIs for the six atomic elements range from 345 mol/m^2^ to 40,423 mol/m^2^ for all buildings. The AUI is much higher for O compared to the other five elements. When comparing across global regions, the ranking of AUIs from largest to smallest closely mirrored that of MUI, a pattern also observed across different building typologies and the comparable global regions for commercial, multifamily residential, and single family residential buildings. This suggests that AUI is largely influenced by MUI, which aligns with the expectation that material-intensive buildings tend to exhibit higher AUIs.

**Figure 4:**
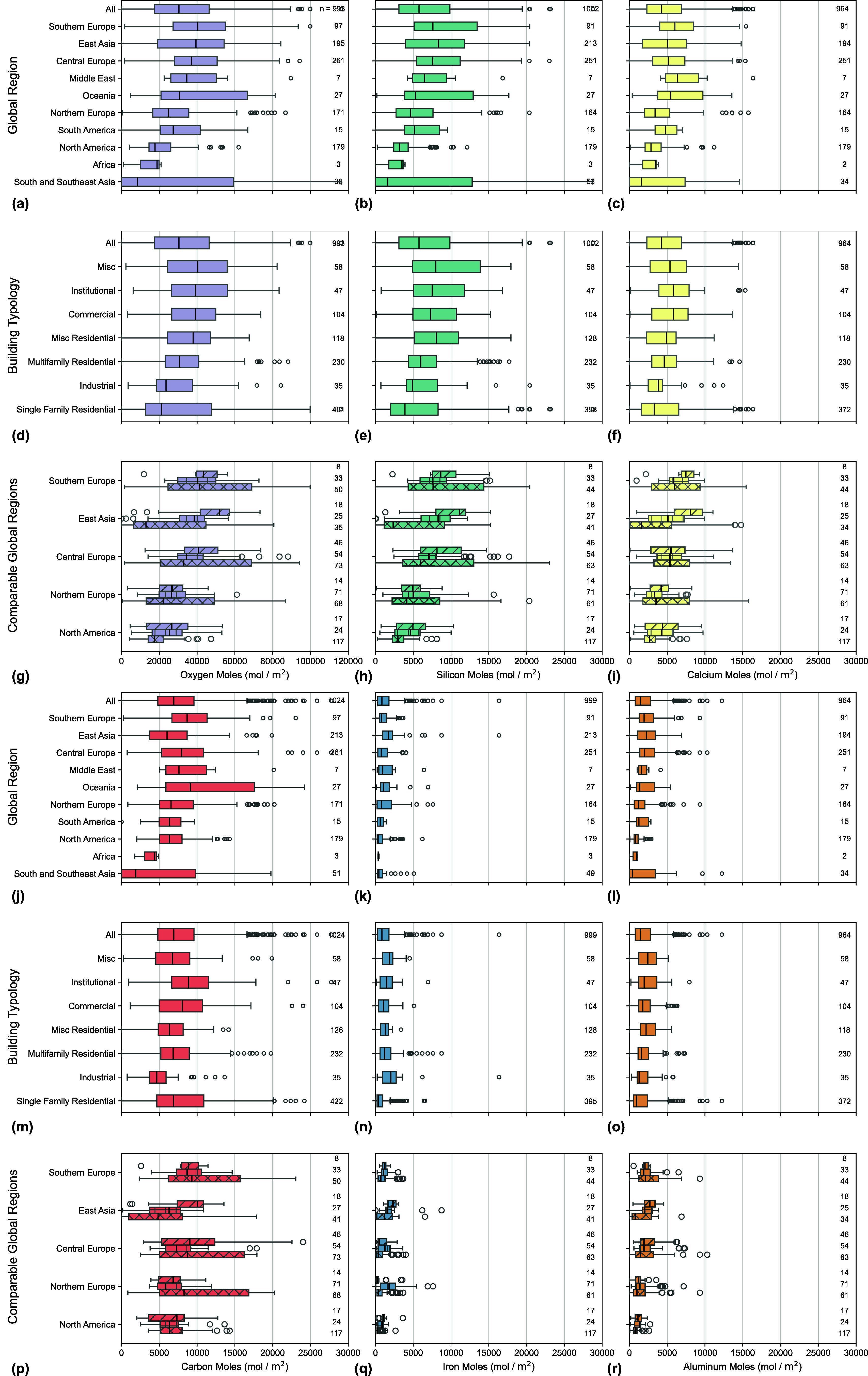
AUIs for the six major chemical elements in buildings. The AUI of (a, d, g) oxygen, (b, e, h) silicon, (c, f, i) calcium, (j, m, p) carbon, (k, n, q) iron, and (l, o, r) aluminum for buildings across (a) global region, (d) building typology, (g) comparable global regions, and respectively. The number of buildings that contain each atomic element, *n*, varies by global region and building typology.

The results of this analysis provide actionable insights for building decarbonization and material flow research. For instance, researchers exploring CO_2_ mineralization as a strategy to sequester carbon during the production of concrete may consider the elemental recovery of Ca from waste concrete.^101–104^ The median AUI for Ca across all global regions and building typologies is 4214 mol/m^2^ (169 kg/m^2^), which could theoretically mineralize 186 kg/m^2^ of CO_2_ as calcium carbonate (CaCO_3_). Thus, a building with a floor area of 5.4 m^2^ could theoretically sequester 1 tonne of CO_2_ if the Ca is carefully recovered, and the CO_2_ is completely mineralized in the concrete over time. A second impactful insight is that the AUI of O and C can guide better estimates of carbon storage potential in buildings.^100,105,106^ Given that buildings have an abundance of O, the AUI of C controls how much carbon storage is theoretically possible in a building. The median AUI for C across all global regions and building types is 6921 mol/m^2^ (83 kg/m^2^), which could store approximately 300 kgCO_2_/m^2^ as stable mineral carbon. A final insight can be gleaned from the AUI of Fe. Successful elemental recovery of Fe in buildings could support supply chains for electronics, like cell phones.^107,108^ The median AUI for Fe across all global regions and building types is 857 mol/m^2^ (48 kg/m^2^), which could theoretically provide enough iron for 1600 cell phones.^107^ These approximate calculations use the averaged AUIs, yet the calculations can be tailored to a specific building type and global region to better inform carbon sequestration and metal supply chains.

### The Atomic Composition of Global Buildings Resembles the Composition of the Earth’s Crust

To further contextualize the AUI results and better understand the sources of atomic elements, the typical atomic composition of a building was compared to the Earth’s crust and the human body. Figure 5 shows the average elemental composition of a global building compared to Earth’s crust (*i*.*e*., the lithosphere)^109^ and the human body.^110^ The comparison reveals that the primary elements that compose a typical building are reflected in the Earth’s crust (see Figure 5b). Five of the six primary atomic elements that constitute a building, namely O, Si, Ca, Fe, and Al, also dominate the composition of the lithosphere. Collectively, these five elements constitute ∼89% of the atomic elements in a building and ∼91% of the atomic elements in the Earth’s crust. This result is not surprising, given that 60% of construction materials are extracted from the lithosphere.^1^ Notably, 0.03% of the lithosphere is composed of C, compared to 8% in buildings, a result that indicates buildings utilize a nontrivial quantity of materials extracted from the biosphere (*e*.*g*., lumber). In contrast, the composition of buildings differs from that of a human body. O is the most prevalent atomic element in buildings and the human body. However, owing to our organic nature, significantly higher quantities of C (19%) and H (9%) are found in humans than in buildings. Recent research suggests that buildings could function as carbon sinks by incorporating substantial quantities of wood, thereby storing more biogenic carbon.^31,105,106^ However, the findings presented here demonstrate that the existing global building stock is overwhelmingly inorganic. Building all new structures or retrofitting existing ones with timber-intensive alternatives would require vast quantities of sustainably harvested lumber—resources that are unevenly distributed and limited in many global regions. Others have similarly concluded that, while promising in theory, such a large-scale transition is scientifically and logistically improbable on a global scale.^30^

**Figure 5:**
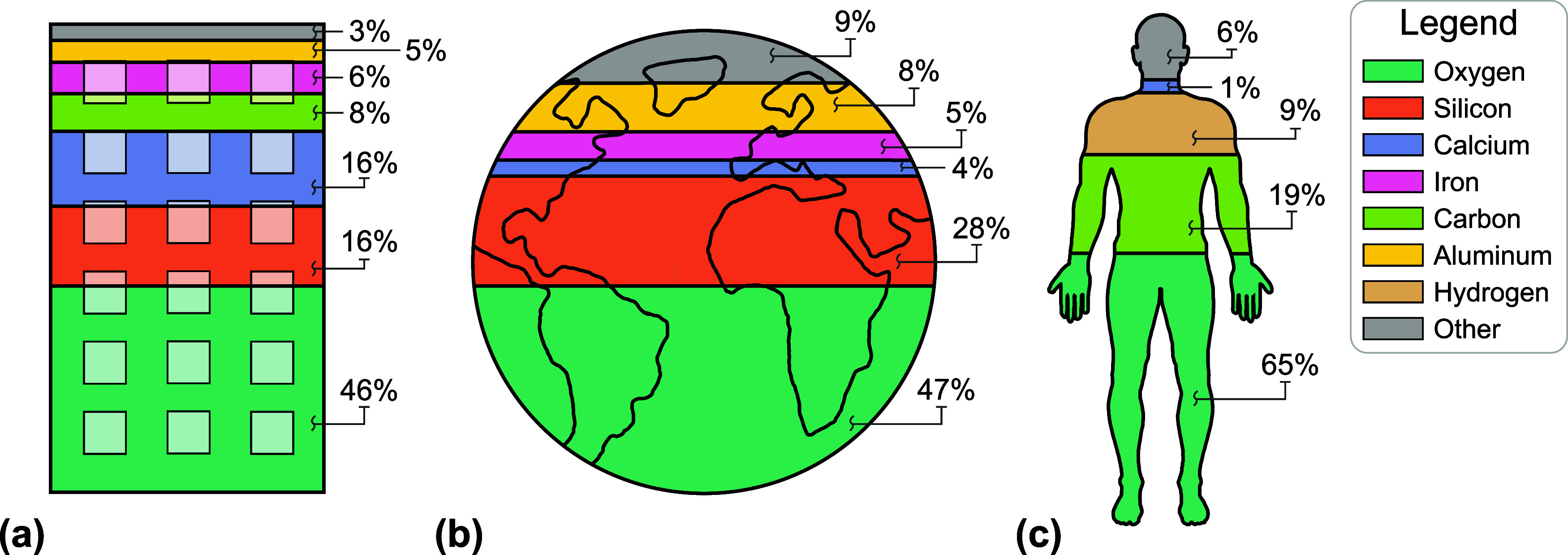
Elemental composition of a building, the Earth’s crust,^109^ and the human body.^110^ The comparison reveals that the elemental composition of the (a) building and the (b) Earth’s crust are quite similar, as five of the six elements that compose a building also compose the crust. However, the elemental composition of a (c) human body differs from a building and the earth’s crust, outside of oxygen as the primarily chemical element across all three entities.

### Implications

The MUIs and AUIs defined herein could inform generalized material flow analyses (MFA) designed to understand the quantity and consumption of materials in the current and future global building stock. Utilizing MFAs to evaluate the bank of materials stored in the building stock is key to enabling a CE of building materials and other industries, like electronics.^32,95–98^ MUI data can potentially inform benchmarks for typical MUIs of different building use types and building components in each global region.^28,111^ AUIs can also support MFAs designed to understand and mitigate the environmental impacts of buildings, such as estimating the carbon-storage potential of the future built environment.^29,105,106,112^ Lastly, the buildings included in the data set may not be fully representative of a specific building typology, or of buildings in a global region, especially in global regions that have few building data entries. Thus, the MUIs and AUIs of this study could be updated as more building material quantity data is made available to better inform carbon sequestration and MFAs.

Material scarcity, construction waste, and the limited applications of CE principles today make the direct reuse or productive recycling of building materials and atomic elements an important area of further study. Assessing the material and elemental compositions of whole buildings can help researchers better understand what resources are available in existing buildings. There are no concerted efforts at the industrial scale for properly extracting constituent elements from existing building materials for use in new materials. Because many common building materials have similar elemental compositions, future CE frameworks could leverage these compositions to remanufacture building materials to reduce material extraction and overall material consumption. Although highly dependent on scientific processing advances in elemental recovery from existing materials, like calcium recovery from concrete,^32–37,101–104^ elemental urban mining from buildings at the end of life could supplement the use of virgin materials (*i*.*e*., metals) that require energy- and carbon-intensive production techniques.^33,113^ As the complete life cycle of material manufacturing and reuse is studied, new research questions about material and atomic supply chains and environmental impacts within the construction sector will become more relevant.

It is anticipated that, over time, the distribution of building materials utilized in the global building stock will shift.^112^ While concrete is likely to continue to grow in global consumption in the coming years, regional consumption of lumber products (*e*.*g*., mass timber) and other biobased materials, such as straw,^114^ is expected to increase.^115,116^ In addition, the growing use of other nontraditional materials will further impact the elemental composition of the global building stock.^117^ Therefore, future analyses like the one presented herein will be needed to best support urban mining efforts and the development and implementation of CE frameworks worldwide.

## Supplementary Material



## Notes

Additional materials and methodological details, including the material use intensities of the buildings analyzed herein (Table S-1); the chemical elemental composition of the building materials (Tables S-2 and S-3); and worked examples to demonstrate the atomic use intensity results (PDF)
