# Supplementary material for: Atomic Building Blocks of Global Buildings
Source: Environ Sci Technol. 2026 Mar 10;60(12):9165–76. doi: 10.1021/acs.est.5c11079 (PMC13045021; doi:10.1021/acs.est.5c11079)
Supplement: Supplementary file 1 [file es5c11079_si_001.pdf]

# Supporting Information

*for the article:*

*Atomic building blocks of global buildings*

# Additional Information on the Material Use Intensities and Elemental Compositions of Building Materials

Jonathan M. Broyles, Matthew A. Jungclaus, Danielle N. Beatty, Wil V. Srubar III

February 2026

**Notes**: Please make use of the *Headings* under the *Navigation* function in Word to browse the organization and sections within this document. Section 1 provides the material use intensities and other information for all buildings analyzed. Section 2 provides the elemental composition breakdown for each building material, in addition to the methodological assumptions made to determine their elemental composition. Section 3 provides the equations and assumptions used to demonstrate the atomic use intensity results. Lastly, additional sources and references are provided in Section 4. This supporting information document includes 4 tables, 3 worked out examples, and references over its 92 pages.

# 1. Material Use Intensities

**Table S-1** conveys the material use intensities (MUIs) for each of the buildings analyzed in the study, using data provided in studies by Heeren and Fishman (2019), Heeren (2021), Guven et al. (2022), Olson (2023), and Athena Sustainable Materials Institute (2024).

**Table S-1.** The global region, building typology, and MUIs for all buildings analyzed in the study herein.

| Building No. | Global Region | Building Typology | MUI (kg/m$^2$) | Source |
|---|---|---|---|---|
| 1 | North America | Institutional | 963.1288708 | Athena |
| 2 | North America | Multifamily Residential | 635.4529562 | Athena |
| 3 | North America | Industrial | 2191.083213 | Athena |
| 4 | North America | Commercial | 1418.330116 | Athena |
| 5 | North America | Multifamily Residential | 1143.382838 | Athena |
| 6 | North America | Commercial | 393.5954023 | Athena |
| 7 | Central Europe | Misc Residential | 1485.999609 | Heeren & Fishman |
| 8 | Central Europe | Misc Residential | 1702.475159 | Heeren & Fishman |
| 9 | Central Europe | Misc Residential | 1630.963412 | Heeren & Fishman |
| 10 | East Asia | Misc | 2079.25 | Heeren & Fishman |
| 11 | East Asia | Misc | 2303 | Heeren & Fishman |
| 12 | East Asia | Misc | 1843 | Heeren & Fishman |
| 13 | East Asia | Misc Residential | 1782.6 | Heeren & Fishman |
| 14 | East Asia | Multifamily Residential | 1607.8 | Heeren & Fishman |
| 15 | East Asia | Misc Residential | 71 | Heeren & Fishman |
| 16 | East Asia | Misc Residential | 52 | Heeren & Fishman |
| 17 | East Asia | Misc Residential | 28 | Heeren & Fishman |
| 18 | East Asia | Misc Residential | 70 | Heeren & Fishman |
| 19 | Central Europe | Misc Residential | 1582.531377 | Heeren & Fishman |
| 20 | East Asia | Misc Residential | 38 | Heeren & Fishman |
| 21 | East Asia | Misc Residential | 15 | Heeren & Fishman |
| 22 | Central Europe | Single Family Residential | 1430.047393 | Heeren & Fishman |
| 23 | Central Europe | Single Family Residential | 1510.185185 | Heeren & Fishman |
| 24 | Central Europe | Single Family Residential | 904.7682119 | Heeren & Fishman |
| 25 | Central Europe | Misc Residential | 1259.587156 | Heeren & Fishman |

| | | | | |
|---|---|---|---|---|
| 26 | Central Europe | Misc Residential | 1259.427313 | Heeren & Fishman |
| 27 | Central Europe | Misc Residential | 1075.903615 | Heeren & Fishman |
| 28 | Central Europe | Misc Residential | 1309.64467 | Heeren & Fishman |
| 29 | Central Europe | Misc Residential | 1322.546839 | Heeren & Fishman |
| 30 | Central Europe | Misc Residential | 1070.57971 | Heeren & Fishman |
| 31 | Central Europe | Multifamily Residential | 1099.335038 | Heeren & Fishman |
| 32 | Central Europe | Multifamily Residential | 1244.690265 | Heeren & Fishman |
| 33 | Central Europe | Multifamily Residential | 1234.392523 | Heeren & Fishman |
| 34 | Central Europe | Multifamily Residential | 1232.630502 | Heeren & Fishman |
| 35 | Central Europe | Multifamily Residential | 1127.677543 | Heeren & Fishman |
| 36 | Central Europe | Multifamily Residential | 1345.125 | Heeren & Fishman |
| 37 | Central Europe | Multifamily Residential | 1269.360269 | Heeren & Fishman |
| 38 | Central Europe | Multifamily Residential | 1154.803172 | Heeren & Fishman |
| 39 | Central Europe | Multifamily Residential | 1139.454418 | Heeren & Fishman |
| 40 | Central Europe | Misc Residential | 1571.264535 | Heeren & Fishman |
| 41 | Central Europe | Multifamily Residential | 1187.912088 | Heeren & Fishman |
| 42 | Central Europe | Multifamily Residential | 2813 | Heeren & Fishman |
| 43 | Central Europe | Multifamily Residential | 2739 | Heeren & Fishman |
| 44 | Central Europe | Multifamily Residential | 2176 | Heeren & Fishman |
| 45 | Central Europe | Multifamily Residential | 2107 | Heeren & Fishman |
| 46 | Central Europe | Multifamily Residential | 2216 | Heeren & Fishman |
| 47 | Central Europe | Misc | 1956.666667 | Heeren & Fishman |
| 48 | North America | Misc Residential | 1427 | Heeren & Fishman |
| 49 | North America | Commercial | 1880 | Heeren & Fishman |

| | | | | |
|---|---|---|---|---|
| 50 | North America | Single Family Residential | 516 | Heeren & Fishman |
| 51 | Central Europe | Misc Residential | 908.6200882 | Heeren & Fishman |
| 52 | North America | Single Family Residential | 1409 | Heeren & Fishman |
| 53 | North America | Industrial | 2129 | Heeren & Fishman |
| 54 | North America | Commercial | 959 | Heeren & Fishman |
| 55 | North America | Institutional | 1464 | Heeren & Fishman |
| 56 | South and Southeast Asia | Single Family Residential | 3858 | Heeren & Fishman |
| 57 | South and Southeast Asia | Single Family Residential | 3149 | Heeren & Fishman |
| 58 | South and Southeast Asia | Multifamily Residential | 2399 | Heeren & Fishman |
| 59 | Southern Europe | Misc Residential | 1453.991816 | Heeren & Fishman |
| 60 | East Asia | Misc | 1002.04 | Heeren & Fishman |
| 61 | East Asia | Single Family Residential | 251 | Heeren & Fishman |
| 62 | Central Europe | Misc Residential | 1090.304826 | Heeren & Fishman |
| 63 | East Asia | Single Family Residential | 362 | Heeren & Fishman |
| 64 | East Asia | Single Family Residential | 370 | Heeren & Fishman |
| 65 | East Asia | Single Family Residential | 444 | Heeren & Fishman |
| 66 | East Asia | Single Family Residential | 451 | Heeren & Fishman |
| 67 | East Asia | Single Family Residential | 487 | Heeren & Fishman |
| 68 | East Asia | Single Family Residential | 473 | Heeren & Fishman |
| 69 | East Asia | Single Family Residential | 476 | Heeren & Fishman |
| 70 | East Asia | Single Family Residential | 531 | Heeren & Fishman |
| 71 | East Asia | Commercial | 1541.3 | Heeren & Fishman |
| 72 | East Asia | Commercial | 2026.3 | Heeren & Fishman |
| 73 | Central Europe | Misc Residential | 1435.865993 | Heeren & Fishman |
| 74 | East Asia | Commercial | 2027.3 | Heeren & Fishman |
| 75 | East Asia | Commercial | 2183.3 | Heeren & Fishman |

| | | | | |
|---|---|---|---|---|
| 76 | East Asia | Industrial | 844 | Heeren & Fishman |
| 77 | East Asia | Industrial | 913 | Heeren & Fishman |
| 78 | East Asia | Industrial | 922 | Heeren & Fishman |
| 79 | East Asia | Industrial | 968 | Heeren & Fishman |
| 80 | East Asia | Industrial | 975 | Heeren & Fishman |
| 81 | East Asia | Industrial | 1027 | Heeren & Fishman |
| 82 | East Asia | Single Family Residential | 488 | Heeren & Fishman |
| 83 | East Asia | Misc | 1164 | Heeren & Fishman |
| 84 | Central Europe | Misc Residential | 1319.929007 | Heeren & Fishman |
| 85 | East Asia | Misc | 962 | Heeren & Fishman |
| 86 | East Asia | Misc | 958 | Heeren & Fishman |
| 87 | East Asia | Misc | 2191 | Heeren & Fishman |
| 88 | Central Europe | Single Family Residential | 2641.221374 | Heeren & Fishman |
| 89 | Central Europe | Single Family Residential | 2039.672043 | Heeren & Fishman |
| 90 | Central Europe | Single Family Residential | 1937.984496 | Heeren & Fishman |
| 91 | Central Europe | Single Family Residential | 2040.40404 | Heeren & Fishman |
| 92 | Central Europe | Multifamily Residential | 2421.511628 | Heeren & Fishman |
| 93 | Central Europe | Multifamily Residential | 1868.533333 | Heeren & Fishman |
| 94 | Central Europe | Multifamily Residential | 1782.407407 | Heeren & Fishman |
| 95 | Central Europe | Misc Residential | 1455.786723 | Heeren & Fishman |
| 96 | Central Europe | Multifamily Residential | 1823.129252 | Heeren & Fishman |
| 97 | East Asia | Misc | 1375 | Heeren & Fishman |
| 98 | Central Europe | Misc Residential | 2100 | Heeren & Fishman |
| 99 | Oceania | Single Family Residential | 285.0128095 | Heeren & Fishman |
| 100 | Oceania | Single Family Residential | 188.3423 | Heeren & Fishman |
| 101 | Northern Europe | Single Family Residential | 450 | Heeren & Fishman |
| 102 | Northern Europe | Single Family Residential | 490 | Heeren & Fishman |

| | | | | |
|---|---|---|---|---|
| 103 | Northern Europe | Multifamily Residential | 371 | Heeren & Fishman |
| 104 | Northern Europe | Multifamily Residential | 821 | Heeren & Fishman |
| 105 | Northern Europe | Commercial | 910 | Heeren & Fishman |
| 106 | Central Europe | Misc Residential | 1103.277148 | Heeren & Fishman |
| 107 | Northern Europe | Misc Residential | 150 | Heeren & Fishman |
| 108 | Northern Europe | Misc Residential | 185 | Heeren & Fishman |
| 109 | Northern Europe | Misc Residential | 200 | Heeren & Fishman |
| 110 | Northern Europe | Misc Residential | 345 | Heeren & Fishman |
| 111 | Northern Europe | Misc Residential | 375 | Heeren & Fishman |
| 112 | Northern Europe | Misc Residential | 435 | Heeren & Fishman |
| 113 | Northern Europe | Misc Residential | 532 | Heeren & Fishman |
| 114 | Northern Europe | Misc Residential | 580 | Heeren & Fishman |
| 115 | Northern Europe | Single Family Residential | 601 | Heeren & Fishman |
| 116 | Northern Europe | Multifamily Residential | 570 | Heeren & Fishman |
| 117 | Central Europe | Misc Residential | 999.7770387 | Heeren & Fishman |
| 118 | Central Europe | Misc Residential | 1813.003953 | Heeren & Fishman |
| 119 | Northern Europe | Commercial | 115 | Heeren & Fishman |
| 120 | Northern Europe | Commercial | 230 | Heeren & Fishman |
| 121 | Northern Europe | Commercial | 375 | Heeren & Fishman |
| 122 | Northern Europe | Commercial | 670 | Heeren & Fishman |
| 123 | Northern Europe | Commercial | 760 | Heeren & Fishman |
| 124 | Northern Europe | Commercial | 915 | Heeren & Fishman |
| 125 | Northern Europe | Commercial | 1035 | Heeren & Fishman |
| 126 | Northern Europe | Commercial | 1128 | Heeren & Fishman |
| 127 | Northern Europe | Multifamily Residential | 1253 | Heeren & Fishman |
| 128 | Northern Europe | Commercial | 1576 | Heeren & Fishman |
| 129 | Central Europe | Misc Residential | 1232.723512 | Heeren & Fishman |
| 130 | South America | Single Family Residential | 1621.7 | Heeren & Fishman |
| 131 | South America | Multifamily Residential | 1451.4 | Heeren & Fishman |
| 132 | Northern Europe | Single Family Residential | 945 | Heeren & Fishman |
| 133 | Northern Europe | Single Family Residential | 814 | Heeren & Fishman |

| | | | | |
|---|---|---|---|---|
| 134 | Northern Europe | Single Family Residential | 923 | Heeren & Fishman |
| 135 | Northern Europe | Single Family Residential | 581 | Heeren & Fishman |
| 136 | Northern Europe | Single Family Residential | 581 | Heeren & Fishman |
| 137 | Northern Europe | Single Family Residential | 455 | Heeren & Fishman |
| 138 | Northern Europe | Single Family Residential | 805 | Heeren & Fishman |
| 139 | Central Europe | Misc Residential | 1745.79123 | Heeren & Fishman |
| 140 | Northern Europe | Single Family Residential | 539 | Heeren & Fishman |
| 141 | Northern Europe | Single Family Residential | 359 | Heeren & Fishman |
| 142 | Northern Europe | Single Family Residential | 521 | Heeren & Fishman |
| 143 | Northern Europe | Single Family Residential | 379 | Heeren & Fishman |
| 144 | Northern Europe | Single Family Residential | 348 | Heeren & Fishman |
| 145 | Northern Europe | Multifamily Residential | 534 | Heeren & Fishman |
| 146 | Northern Europe | Multifamily Residential | 913 | Heeren & Fishman |
| 147 | Northern Europe | Multifamily Residential | 492 | Heeren & Fishman |
| 148 | Northern Europe | Multifamily Residential | 769 | Heeren & Fishman |
| 149 | Northern Europe | Multifamily Residential | 665 | Heeren & Fishman |
| 150 | Central Europe | Misc Residential | 1809.844311 | Heeren & Fishman |
| 151 | Northern Europe | Multifamily Residential | 897.4 | Heeren & Fishman |
| 152 | Northern Europe | Multifamily Residential | 752.3 | Heeren & Fishman |
| 153 | Northern Europe | Multifamily Residential | 861 | Heeren & Fishman |
| 154 | Northern Europe | Multifamily Residential | 834 | Heeren & Fishman |

| | | | | |
|---|---|---|---|---|
| 155 | Northern Europe | Multifamily Residential | 1448 | Heeren & Fishman |
| 156 | Northern Europe | Multifamily Residential | 1323 | Heeren & Fishman |
| 157 | Northern Europe | Multifamily Residential | 1548 | Heeren & Fishman |
| 158 | Northern Europe | Multifamily Residential | 1512 | Heeren & Fishman |
| 159 | Northern Europe | Multifamily Residential | 1630 | Heeren & Fishman |
| 160 | Northern Europe | Multifamily Residential | 1470 | Heeren & Fishman |
| 161 | Central Europe | Misc Residential | 1179.4 | Heeren & Fishman |
| 162 | Northern Europe | Multifamily Residential | 1066.8 | Heeren & Fishman |
| 163 | Northern Europe | Multifamily Residential | 1401 | Heeren & Fishman |
| 164 | Northern Europe | Multifamily Residential | 894 | Heeren & Fishman |
| 165 | Northern Europe | Multifamily Residential | 1240 | Heeren & Fishman |
| 166 | Northern Europe | Multifamily Residential | 1365 | Heeren & Fishman |
| 167 | Northern Europe | Multifamily Residential | 1231 | Heeren & Fishman |
| 168 | Northern Europe | Multifamily Residential | 1046 | Heeren & Fishman |
| 169 | Northern Europe | Multifamily Residential | 1025 | Heeren & Fishman |
| 170 | Northern Europe | Multifamily Residential | 838 | Heeren & Fishman |
| 171 | Northern Europe | Multifamily Residential | 898 | Heeren & Fishman |
| 172 | Central Europe | Misc Residential | 1187.458638 | Heeren & Fishman |
| 173 | Northern Europe | Multifamily Residential | 1149.9 | Heeren & Fishman |
| 174 | Northern Europe | Multifamily Residential | 973.5 | Heeren & Fishman |
| 175 | Northern Europe | Multifamily Residential | 969.2 | Heeren & Fishman |

| | | | | |
|---|---|---|---|---|
| 176 | Northern Europe | Multifamily Residential | 822 | Heeren & Fishman |
| 177 | Northern Europe | Multifamily Residential | 1062.5 | Heeren & Fishman |
| 178 | Northern Europe | Multifamily Residential | 964.3 | Heeren & Fishman |
| 179 | Northern Europe | Multifamily Residential | 811 | Heeren & Fishman |
| 180 | Northern Europe | Multifamily Residential | 1160 | Heeren & Fishman |
| 181 | Northern Europe | Multifamily Residential | 943.4 | Heeren & Fishman |
| 182 | Central Europe | Misc | 1259.271678 | Heeren & Fishman |
| 183 | Central Europe | Misc Residential | 2124.347555 | Heeren & Fishman |
| 184 | East Asia | Misc Residential | 1695.65 | Heeren & Fishman |
| 185 | East Asia | Industrial | 2326.89 | Heeren & Fishman |
| 186 | East Asia | Commercial | 1784.96 | Heeren & Fishman |
| 187 | East Asia | Misc | 1933.65 | Heeren & Fishman |
| 188 | East Asia | Misc | 808.78 | Heeren & Fishman |
| 189 | East Asia | Misc | 1629.48 | Heeren & Fishman |
| 190 | East Asia | Misc | 1082 | Heeren & Fishman |
| 191 | East Asia | Misc | 1783 | Heeren & Fishman |
| 192 | East Asia | Misc | 582 | Heeren & Fishman |
| 193 | East Asia | Misc | 1197 | Heeren & Fishman |
| 194 | Central Europe | Misc Residential | 1501.400853 | Heeren & Fishman |
| 195 | Central Europe | Misc Residential | 783 | Heeren & Fishman |
| 196 | Central Europe | Misc Residential | 965 | Heeren & Fishman |
| 197 | Central Europe | Commercial | 734 | Heeren & Fishman |
| 198 | Central Europe | Industrial | 1060 | Heeren & Fishman |
| 199 | North America | Misc Residential | 609.07 | Heeren & Fishman |
| 200 | North America | Commercial | 686.31 | Heeren & Fishman |
| 201 | North America | Industrial | 675.4 | Heeren & Fishman |
| 202 | North America | Misc | 555.8333333 | Heeren & Fishman |
| 203 | North America | Single Family Residential | 480.0861141 | Heeren & Fishman |
| 204 | North America | Single Family Residential | 499.4617869 | Heeren & Fishman |
| 205 | Central Europe | Misc Residential | 615.9820741 | Heeren & Fishman |
| 206 | North America | Single Family Residential | 600.6458557 | Heeren & Fishman |

| | | | | |
|---|---|---|---|---|
| 207 | North America | Multifamily Residential | 618.9451023 | Heeren & Fishman |
| 208 | North America | Multifamily Residential | 188.3745963 | Heeren & Fishman |
| 209 | North America | Multifamily Residential | 195.9095802 | Heeren & Fishman |
| 210 | North America | Commercial | 243.2723359 | Heeren & Fishman |
| 211 | North America | Commercial | 579.0986298 | Heeren & Fishman |
| 212 | North America | Misc | 1320.732377 | Heeren & Fishman |
| 213 | North America | Industrial | 1101.14851 | Heeren & Fishman |
| 214 | North America | Industrial | 656.5988181 | Heeren & Fishman |
| 215 | Central Europe | Misc Residential | 1635.235602 | Heeren & Fishman |
| 216 | North America | Misc | 776.0782752 | Heeren & Fishman |
| 217 | North America | Misc | 814.8283694 | Heeren & Fishman |
| 218 | South and Southeast Asia | Single Family Residential | 2263.7 | Heeren & Fishman |
| 219 | South and Southeast Asia | Single Family Residential | 1883.2 | Heeren & Fishman |
| 220 | South and Southeast Asia | Single Family Residential | 2062.3 | Heeren & Fishman |
| 221 | South and Southeast Asia | Single Family Residential | 2227 | Heeren & Fishman |
| 222 | South and Southeast Asia | Single Family Residential | 2047.8 | Heeren & Fishman |
| 223 | South and Southeast Asia | Single Family Residential | 2259.3 | Heeren & Fishman |
| 224 | South and Southeast Asia | Single Family Residential | 2144.3 | Heeren & Fishman |
| 225 | South and Southeast Asia | Single Family Residential | 2063.6 | Heeren & Fishman |
| 226 | Central Europe | Misc Residential | 1166.648134 | Heeren & Fishman |
| 227 | Central Europe | Misc Residential | 1869.888547 | Heeren & Fishman |
| 228 | South and Southeast Asia | Commercial | 2495.75 | Heeren & Fishman |
| 229 | Central Europe | Institutional | 1991.305821 | Heeren & Fishman |
| 230 | Central Europe | Multifamily Residential | 1481.8 | Heeren & Fishman |
| 231 | Central Europe | Institutional | 1478.224 | Heeren & Fishman |
| 232 | Central Europe | Industrial | 1454.650077 | Heeren & Fishman |
| 233 | Central Europe | Commercial | 1587.367273 | Heeren & Fishman |

| | | | | |
|---|---|---|---|---|
| 234 | East Asia | Commercial | 2019.55103 | Heeren & Fishman |
| 235 | Southern Europe | Commercial | 1481.659969 | Heeren & Fishman |
| 236 | Southern Europe | Commercial | 2008.4275 | Heeren & Fishman |
| 237 | Northern Europe | Institutional | 1303.32298 | Heeren & Fishman |
| 238 | Central Europe | Misc Residential | 665.2312184 | Heeren & Fishman |
| 239 | North America | Institutional | 341.97 | Heeren & Fishman |
| 240 | East Asia | Institutional | 2894.21 | Heeren & Fishman |
| 241 | Oceania | Institutional | 1746.191702 | Heeren & Fishman |
| 242 | East Asia | Misc Residential | 1892 | Heeren & Fishman |
| 243 | East Asia | Misc Residential | 2093 | Heeren & Fishman |
| 244 | East Asia | Misc Residential | 2303 | Heeren & Fishman |
| 245 | East Asia | Misc Residential | 1388 | Heeren & Fishman |
| 246 | East Asia | Misc Residential | 1553 | Heeren & Fishman |
| 247 | East Asia | Misc Residential | 1843 | Heeren & Fishman |
| 248 | East Asia | Misc Residential | 2079.25 | Heeren & Fishman |
| 249 | Central Europe | Misc Residential | 1469.664165 | Heeren & Fishman |
| 250 | East Asia | Misc Residential | 2303 | Heeren & Fishman |
| 251 | East Asia | Misc Residential | 1843 | Heeren & Fishman |
| 252 | East Asia | Misc | 1861 | Heeren & Fishman |
| 253 | East Asia | Misc | 1927 | Heeren & Fishman |
| 254 | East Asia | Misc | 2086 | Heeren & Fishman |
| 255 | East Asia | Misc | 1588 | Heeren & Fishman |
| 256 | East Asia | Misc | 2050 | Heeren & Fishman |
| 257 | East Asia | Misc | 2229 | Heeren & Fishman |
| 258 | East Asia | Misc Residential | 2241 | Heeren & Fishman |
| 259 | East Asia | Industrial | 2965 | Heeren & Fishman |
| 260 | Central Europe | Misc Residential | 740.4529907 | Heeren & Fishman |
| 261 | East Asia | Commercial | 2027 | Heeren & Fishman |
| 262 | East Asia | Commercial | 2385 | Heeren & Fishman |
| 263 | East Asia | Institutional | 2160 | Heeren & Fishman |
| 264 | East Asia | Institutional | 2180 | Heeren & Fishman |
| 265 | East Asia | Industrial | 1023 | Heeren & Fishman |
| 266 | East Asia | Misc | 2141 | Heeren & Fishman |
| 267 | North America | Multifamily Residential | 1090.552885 | Heeren & Fishman |
| 268 | Middle East | Multifamily Residential | 1027.829533 | Heeren & Fishman |
| 269 | East Asia | Multifamily Residential | 1723.443157 | Heeren & Fishman |

| | | | | |
|---|---|---|---|---|
| 270 | East Asia | Multifamily Residential | 1635.488817 | Heeren & Fishman |
| 271 | Central Europe | Misc Residential | 1387.600831 | Heeren & Fishman |
| 272 | East Asia | Multifamily Residential | 1667.129726 | Heeren & Fishman |
| 273 | East Asia | Commercial | 1650.424616 | Heeren & Fishman |
| 274 | East Asia | Multifamily Residential | 1931.445515 | Heeren & Fishman |
| 275 | East Asia | Multifamily Residential | 1998.489023 | Heeren & Fishman |
| 276 | East Asia | Multifamily Residential | 1709.111111 | Heeren & Fishman |
| 277 | East Asia | Multifamily Residential | 1977.491529 | Heeren & Fishman |
| 278 | North America | Single Family Residential | 312.402222 | Heeren & Fishman |
| 279 | North America | Single Family Residential | 639.02696 | Heeren & Fishman |
| 280 | North America | Multifamily Residential | 1900.936803 | Heeren & Fishman |
| 281 | North America | Single Family Residential | 884.7277213 | Heeren & Fishman |
| 282 | Central Europe | Misc Residential | 2221.056217 | Heeren & Fishman |
| 283 | North America | Single Family Residential | 584.8811189 | Heeren & Fishman |
| 284 | South America | Multifamily Residential | 496.3978261 | Heeren & Fishman |
| 285 | South America | Multifamily Residential | 965.2959266 | Heeren & Fishman |
| 286 | South America | Single Family Residential | 782.5782918 | Heeren & Fishman |
| 287 | South America | Single Family Residential | 1065.089286 | Heeren & Fishman |
| 288 | South America | Single Family Residential | 286.15625 | Heeren & Fishman |
| 289 | South America | Multifamily Residential | 953.2857143 | Heeren & Fishman |
| 290 | Africa | Single Family Residential | 731.9916345 | Heeren & Fishman |
| 291 | Africa | Single Family Residential | 656.8191057 | Heeren & Fishman |

| | | | | |
|---|---|---|---|---|
| 292 | Africa | Single Family Residential | 68.78549383 | Heeren & Fishman |
| 293 | Central Europe | Misc Residential | 1845.638718 | Heeren & Fishman |
| 294 | Southern Europe | Single Family Residential | 91.84722222 | Heeren & Fishman |
| 295 | Southern Europe | Single Family Residential | 58.16666667 | Heeren & Fishman |
| 296 | Southern Europe | Single Family Residential | 64.9375 | Heeren & Fishman |
| 297 | Southern Europe | Single Family Residential | 1827.507599 | Heeren & Fishman |
| 298 | Southern Europe | Single Family Residential | 2483.282675 | Heeren & Fishman |
| 299 | Southern Europe | Single Family Residential | 2483.282675 | Heeren & Fishman |
| 300 | Southern Europe | Single Family Residential | 2457.953394 | Heeren & Fishman |
| 301 | Southern Europe | Single Family Residential | 2895.719351 | Heeren & Fishman |
| 302 | Central Europe | Misc Residential | 1169.610265 | Heeren & Fishman |
| 303 | Southern Europe | Single Family Residential | 2483.156028 | Heeren & Fishman |
| 304 | Southern Europe | Single Family Residential | 2483.156028 | Heeren & Fishman |
| 305 | Southern Europe | Single Family Residential | 222.0586431 | Heeren & Fishman |
| 306 | Southern Europe | Single Family Residential | 64.0542522 | Heeren & Fishman |
| 307 | Southern Europe | Single Family Residential | 64.0542522 | Heeren & Fishman |
| 308 | Southern Europe | Single Family Residential | 540.0845373 | Heeren & Fishman |
| 309 | Southern Europe | Single Family Residential | 937.7653928 | Heeren & Fishman |
| 310 | Southern Europe | Single Family Residential | 937.7653928 | Heeren & Fishman |
| 311 | Southern Europe | Single Family Residential | 1436.054125 | Heeren & Fishman |
| 312 | Southern Europe | Single Family Residential | 946.8178348 | Heeren & Fishman |
| 313 | Central Europe | Misc Residential | 1651.681369 | Heeren & Fishman |

| | | | | |
|---|---|---|---|---|
| 314 | Southern Europe | Single Family Residential | 946.8178348 | Heeren & Fishman |
| 315 | Southern Europe | Multifamily Residential | 1464.521176 | Heeren & Fishman |
| 316 | Southern Europe | Multifamily Residential | 1023.289892 | Heeren & Fishman |
| 317 | Southern Europe | Multifamily Residential | 1023.289892 | Heeren & Fishman |
| 318 | Southern Europe | Multifamily Residential | 780.9064656 | Heeren & Fishman |
| 319 | Southern Europe | Multifamily Residential | 794.6538104 | Heeren & Fishman |
| 320 | Southern Europe | Multifamily Residential | 1141.156463 | Heeren & Fishman |
| 321 | Central Europe | Single Family Residential | 68.58333333 | Heeren & Fishman |
| 322 | Central Europe | Single Family Residential | 87.33333333 | Heeren & Fishman |
| 323 | Central Europe | Single Family Residential | 222.0586431 | Heeren & Fishman |
| 324 | Central Europe | Misc Residential | 1646.444114 | Heeren & Fishman |
| 325 | Central Europe | Single Family Residential | 1826.747721 | Heeren & Fishman |
| 326 | Central Europe | Single Family Residential | 2540.225417 | Heeren & Fishman |
| 327 | Central Europe | Single Family Residential | 2425.139319 | Heeren & Fishman |
| 328 | Central Europe | Single Family Residential | 2428.235294 | Heeren & Fishman |
| 329 | Central Europe | Single Family Residential | 2375 | Heeren & Fishman |
| 330 | Central Europe | Single Family Residential | 2375 | Heeren & Fishman |
| 331 | Central Europe | Single Family Residential | 1659.605058 | Heeren & Fishman |
| 332 | Central Europe | Single Family Residential | 1659.605058 | Heeren & Fishman |
| 333 | Central Europe | Single Family Residential | 59.94868035 | Heeren & Fishman |
| 334 | Central Europe | Single Family Residential | 64.0542522 | Heeren & Fishman |

| | | | | |
|---|---|---|---|---|
| 335 | Central Europe | Misc Residential | 1429.972376 | Heeren & Fishman |
| 336 | Central Europe | Misc Residential | 1339.484039 | Heeren & Fishman |
| 337 | Central Europe | Single Family Residential | 746.3668749 | Heeren & Fishman |
| 338 | Central Europe | Single Family Residential | 1106.219968 | Heeren & Fishman |
| 339 | Central Europe | Single Family Residential | 728.7301588 | Heeren & Fishman |
| 340 | Central Europe | Single Family Residential | 728.7301588 | Heeren & Fishman |
| 341 | Central Europe | Single Family Residential | 620.641914 | Heeren & Fishman |
| 342 | Central Europe | Single Family Residential | 620.641914 | Heeren & Fishman |
| 343 | Central Europe | Single Family Residential | 714.2923396 | Heeren & Fishman |
| 344 | Central Europe | Single Family Residential | 1484.152663 | Heeren & Fishman |
| 345 | Central Europe | Multifamily Residential | 1430.298728 | Heeren & Fishman |
| 346 | Central Europe | Multifamily Residential | 1043.039158 | Heeren & Fishman |
| 347 | Central Europe | Misc Residential | 1243.078209 | Heeren & Fishman |
| 348 | Central Europe | Multifamily Residential | 576.3927528 | Heeren & Fishman |
| 349 | Central Europe | Multifamily Residential | 576.3927528 | Heeren & Fishman |
| 350 | Northern Europe | Single Family Residential | 87.33333333 | Heeren & Fishman |
| 351 | Northern Europe | Single Family Residential | 2980.911983 | Heeren & Fishman |
| 352 | Northern Europe | Single Family Residential | 423.6434109 | Heeren & Fishman |
| 353 | Northern Europe | Single Family Residential | 2381.269756 | Heeren & Fishman |
| 354 | Northern Europe | Single Family Residential | 2574.468085 | Heeren & Fishman |
| 355 | Northern Europe | Single Family Residential | 2784.891641 | Heeren & Fishman |
| 356 | Northern Europe | Single Family Residential | 2428.235294 | Heeren & Fishman |

| | | | | |
|---|---|---|---|---|
| 357 | Northern Europe | Single Family Residential | 1659.605058 | Heeren & Fishman |
| 358 | Central Europe | Misc Residential | 1163.377656 | Heeren & Fishman |
| 359 | Northern Europe | Single Family Residential | 1659.605058 | Heeren & Fishman |
| 360 | Northern Europe | Single Family Residential | 59.94868035 | Heeren & Fishman |
| 361 | Northern Europe | Single Family Residential | 1214.332607 | Heeren & Fishman |
| 362 | Northern Europe | Single Family Residential | 20.35819511 | Heeren & Fishman |
| 363 | Northern Europe | Single Family Residential | 1133.191678 | Heeren & Fishman |
| 364 | Northern Europe | Single Family Residential | 1462.261146 | Heeren & Fishman |
| 365 | Northern Europe | Single Family Residential | 194.6760521 | Heeren & Fishman |
| 366 | Northern Europe | Single Family Residential | 194.6760521 | Heeren & Fishman |
| 367 | Northern Europe | Single Family Residential | 728.7301588 | Heeren & Fishman |
| 368 | Northern Europe | Single Family Residential | 728.7301588 | Heeren & Fishman |
| 369 | Central Europe | Misc Residential | 846.4291398 | Heeren & Fishman |
| 370 | Northern Europe | Multifamily Residential | 1396.979466 | Heeren & Fishman |
| 371 | Northern Europe | Multifamily Residential | 1043.039158 | Heeren & Fishman |
| 372 | Northern Europe | Multifamily Residential | 582.3696145 | Heeren & Fishman |
| 373 | Northern Europe | Multifamily Residential | 582.3696145 | Heeren & Fishman |
| 374 | Central Europe | Multifamily Residential | 1264.35463 | Heeren & Fishman |
| 375 | Central Europe | Single Family Residential | 848.7608081 | Heeren & Fishman |
| 376 | Central Europe | Multifamily Residential | 1089.683194 | Heeren & Fishman |
| 377 | Central Europe | Single Family Residential | 450.1461539 | Heeren & Fishman |

| | | | | |
|---|---|---|---|---|
| 378 | Central Europe | Multifamily Residential | 485.2444444 | Heeren & Fishman |
| 379 | Central Europe | Multifamily Residential | 520.1 | Heeren & Fishman |
| 380 | Central Europe | Misc Residential | 1746.786304 | Heeren & Fishman |
| 381 | Central Europe | Single Family Residential | 907.9036827 | Heeren & Fishman |
| 382 | Central Europe | Multifamily Residential | 1082.358974 | Heeren & Fishman |
| 383 | Southern Europe | Single Family Residential | 782 | Heeren & Fishman |
| 384 | Southern Europe | Single Family Residential | 855 | Heeren & Fishman |
| 385 | Southern Europe | Single Family Residential | 243 | Heeren & Fishman |
| 386 | Southern Europe | Single Family Residential | 1163 | Heeren & Fishman |
| 387 | Southern Europe | Single Family Residential | 1304 | Heeren & Fishman |
| 388 | Southern Europe | Single Family Residential | 1334 | Heeren & Fishman |
| 389 | Southern Europe | Multifamily Residential | 1014 | Heeren & Fishman |
| 390 | Southern Europe | Multifamily Residential | 1217 | Heeren & Fishman |
| 391 | Central Europe | Misc Residential | 1362.015899 | Heeren & Fishman |
| 392 | Southern Europe | Multifamily Residential | 1391 | Heeren & Fishman |
| 393 | Central Europe | Single Family Residential | 1135.930233 | Heeren & Fishman |
| 394 | Central Europe | Single Family Residential | 1081.469808 | Heeren & Fishman |
| 395 | Central Europe | Single Family Residential | 755.5283557 | Heeren & Fishman |
| 396 | Central Europe | Single Family Residential | 862.0838434 | Heeren & Fishman |
| 397 | Central Europe | Single Family Residential | 68.58333333 | Heeren & Fishman |
| 398 | Central Europe | Single Family Residential | 87.33333333 | Heeren & Fishman |

| | | | | |
|---|---|---|---|---|
| 399 | Central Europe | Single Family Residential | 222.0586431 | Heeren & Fishman |
| 400 | Central Europe | Single Family Residential | 1826.747721 | Heeren & Fishman |
| 401 | Central Europe | Single Family Residential | 2540.225417 | Heeren & Fishman |
| 402 | Central Europe | Misc Residential | 765.8149026 | Heeren & Fishman |
| 403 | Central Europe | Single Family Residential | 2425.139319 | Heeren & Fishman |
| 404 | Central Europe | Single Family Residential | 2428.235294 | Heeren & Fishman |
| 405 | Central Europe | Single Family Residential | 2375 | Heeren & Fishman |
| 406 | Central Europe | Single Family Residential | 2375 | Heeren & Fishman |
| 407 | Central Europe | Single Family Residential | 1659.605058 | Heeren & Fishman |
| 408 | Central Europe | Single Family Residential | 1659.605058 | Heeren & Fishman |
| 409 | Central Europe | Single Family Residential | 59.94868035 | Heeren & Fishman |
| 410 | Central Europe | Single Family Residential | 64.0542522 | Heeren & Fishman |
| 411 | Central Europe | Single Family Residential | 746.3668749 | Heeren & Fishman |
| 412 | Central Europe | Single Family Residential | 1106.219968 | Heeren & Fishman |
| 413 | Central Europe | Misc Residential | 1383.26535 | Heeren & Fishman |
| 414 | Central Europe | Single Family Residential | 728.7301588 | Heeren & Fishman |
| 415 | Central Europe | Single Family Residential | 728.7301588 | Heeren & Fishman |
| 416 | Central Europe | Single Family Residential | 620.641914 | Heeren & Fishman |
| 417 | Central Europe | Single Family Residential | 620.641914 | Heeren & Fishman |
| 418 | Central Europe | Single Family Residential | 714.2923396 | Heeren & Fishman |
| 419 | Central Europe | Single Family Residential | 1484.152663 | Heeren & Fishman |

| | | | | |
|---|---|---|---|---|
| 420 | Central Europe | Multifamily Residential | 1430.298728 | Heeren & Fishman |
| 421 | Central Europe | Multifamily Residential | 1043.039158 | Heeren & Fishman |
| 422 | Central Europe | Multifamily Residential | 576.3927528 | Heeren & Fishman |
| 423 | Central Europe | Multifamily Residential | 576.3927528 | Heeren & Fishman |
| 424 | Central Europe | Misc Residential | 1458.156201 | Heeren & Fishman |
| 425 | Northern Europe | Single Family Residential | 87.33333333 | Heeren & Fishman |
| 426 | Northern Europe | Single Family Residential | 2980.911983 | Heeren & Fishman |
| 427 | Northern Europe | Single Family Residential | 423.6434109 | Heeren & Fishman |
| 428 | Northern Europe | Single Family Residential | 2381.269756 | Heeren & Fishman |
| 429 | Northern Europe | Single Family Residential | 2574.468085 | Heeren & Fishman |
| 430 | Northern Europe | Single Family Residential | 2784.891641 | Heeren & Fishman |
| 431 | Northern Europe | Single Family Residential | 2428.235294 | Heeren & Fishman |
| 432 | Northern Europe | Single Family Residential | 1659.605058 | Heeren & Fishman |
| 433 | Northern Europe | Single Family Residential | 1659.605058 | Heeren & Fishman |
| 434 | Northern Europe | Single Family Residential | 59.94868035 | Heeren & Fishman |
| 435 | Central Europe | Misc Residential | 1338.35052 | Heeren & Fishman |
| 436 | Northern Europe | Single Family Residential | 1229.948877 | Heeren & Fishman |
| 437 | Northern Europe | Single Family Residential | 814.8802379 | Heeren & Fishman |
| 438 | Northern Europe | Single Family Residential | 1133.191678 | Heeren & Fishman |
| 439 | Northern Europe | Single Family Residential | 1462.261146 | Heeren & Fishman |
| 440 | Northern Europe | Single Family Residential | 194.6760521 | Heeren & Fishman |

| | | | | |
|---|---|---|---|---|
| 441 | Northern Europe | Single Family Residential | 194.6760521 | Heeren & Fishman |
| 442 | Northern Europe | Single Family Residential | 728.7301588 | Heeren & Fishman |
| 443 | Northern Europe | Single Family Residential | 728.7301588 | Heeren & Fishman |
| 444 | Northern Europe | Multifamily Residential | 1396.979466 | Heeren & Fishman |
| 445 | Northern Europe | Multifamily Residential | 1043.039158 | Heeren & Fishman |
| 446 | Central Europe | Misc Residential | 1373.797739 | Heeren & Fishman |
| 447 | Central Europe | Commercial | 1938.133267 | Heeren & Fishman |
| 448 | Northern Europe | Multifamily Residential | 582.3696145 | Heeren & Fishman |
| 449 | Northern Europe | Multifamily Residential | 582.3696145 | Heeren & Fishman |
| 450 | Middle East | Multifamily Residential | 816.9975385 | Heeren & Fishman |
| 451 | Middle East | Single Family Residential | 1592.711819 | Heeren & Fishman |
| 452 | Middle East | Single Family Residential | 2261.506637 | Heeren & Fishman |
| 453 | Middle East | Single Family Residential | 3180.657652 | Heeren & Fishman |
| 454 | Middle East | Single Family Residential | 781.0493828 | Heeren & Fishman |
| 455 | Middle East | Single Family Residential | 1187.037037 | Heeren & Fishman |
| 456 | South and Southeast Asia | Multifamily Residential | 1854.808321 | Heeren & Fishman |
| 457 | South and Southeast Asia | Single Family Residential | 16 | Heeren & Fishman |
| 458 | Central Europe | Commercial | 1747.433628 | Heeren & Fishman |
| 459 | South and Southeast Asia | Single Family Residential | 15.1 | Heeren & Fishman |
| 460 | South and Southeast Asia | Single Family Residential | 21.1 | Heeren & Fishman |
| 461 | South and Southeast Asia | Single Family Residential | 20.9 | Heeren & Fishman |
| 462 | South and Southeast Asia | Single Family Residential | 1396 | Heeren & Fishman |

| | | | | |
|---|---|---|---|---|
| 463 | South and Southeast Asia | Single Family Residential | 1052.5 | Heeren & Fishman |
| 464 | South and Southeast Asia | Single Family Residential | 1028.8 | Heeren & Fishman |
| 465 | South and Southeast Asia | Single Family Residential | 941.2 | Heeren & Fishman |
| 466 | South and Southeast Asia | Single Family Residential | 436 | Heeren & Fishman |
| 467 | South and Southeast Asia | Single Family Residential | 330.9 | Heeren & Fishman |
| 468 | South and Southeast Asia | Single Family Residential | 328 | Heeren & Fishman |
| 469 | Central Europe | Commercial | 1871.562566 | Heeren & Fishman |
| 470 | South and Southeast Asia | Single Family Residential | 301.2 | Heeren & Fishman |
| 471 | South and Southeast Asia | Single Family Residential | 16 | Heeren & Fishman |
| 472 | South and Southeast Asia | Single Family Residential | 15.2 | Heeren & Fishman |
| 473 | South and Southeast Asia | Single Family Residential | 21.4 | Heeren & Fishman |
| 474 | South and Southeast Asia | Single Family Residential | 21.2 | Heeren & Fishman |
| 475 | South and Southeast Asia | Single Family Residential | 2746 | Heeren & Fishman |
| 476 | South and Southeast Asia | Single Family Residential | 2066.6 | Heeren & Fishman |
| 477 | South and Southeast Asia | Single Family Residential | 2013 | Heeren & Fishman |
| 478 | South and Southeast Asia | Single Family Residential | 1838.6 | Heeren & Fishman |
| 479 | South and Southeast Asia | Single Family Residential | 16 | Heeren & Fishman |
| 480 | Central Europe | Commercial | 1799.912635 | Heeren & Fishman |
| 481 | South and Southeast Asia | Single Family Residential | 15.2 | Heeren & Fishman |
| 482 | South and Southeast Asia | Single Family Residential | 21.4 | Heeren & Fishman |
| 483 | South and Southeast Asia | Single Family Residential | 21.2 | Heeren & Fishman |

| | | | | |
|---|---|---|---|---|
| 484 | South and Southeast Asia | Single Family Residential | 16 | Heeren & Fishman |
| 485 | South and Southeast Asia | Single Family Residential | 15.2 | Heeren & Fishman |
| 486 | South and Southeast Asia | Single Family Residential | 21.4 | Heeren & Fishman |
| 487 | South and Southeast Asia | Single Family Residential | 21.2 | Heeren & Fishman |
| 488 | South and Southeast Asia | Single Family Residential | 54.18949772 | Heeren & Fishman |
| 489 | South and Southeast Asia | Single Family Residential | 102.6380767 | Heeren & Fishman |
| 490 | South and Southeast Asia | Single Family Residential | 2.599090318 | Heeren & Fishman |
| 491 | Central Europe | Commercial | 1442.527208 | Heeren & Fishman |
| 492 | South and Southeast Asia | Single Family Residential | 48.27777778 | Heeren & Fishman |
| 493 | East Asia | Multifamily Residential | 1366.761567 | Heeren & Fishman |
| 494 | East Asia | Single Family Residential | 2253.069491 | Heeren & Fishman |
| 495 | East Asia | Single Family Residential | 2124.639249 | Heeren & Fishman |
| 496 | East Asia | Single Family Residential | 2900.659955 | Heeren & Fishman |
| 497 | East Asia | Single Family Residential | 2257.119487 | Heeren & Fishman |
| 498 | East Asia | Single Family Residential | 2689.049323 | Heeren & Fishman |
| 499 | East Asia | Single Family Residential | 2329.594946 | Heeren & Fishman |
| 500 | East Asia | Multifamily Residential | 1576.213612 | Heeren & Fishman |
| 501 | East Asia | Single Family Residential | 389.3390862 | Heeren & Fishman |
| 502 | Central Europe | Commercial | 1812.573185 | Heeren & Fishman |
| 503 | East Asia | Single Family Residential | 2661.84013 | Heeren & Fishman |
| 504 | South and Southeast Asia | Single Family Residential | 1377.42 | Heeren & Fishman |

| | | | | |
|---|---|---|---|---|
| 505 | South and Southeast Asia | Single Family Residential | 1043.46 | Heeren & Fishman |
| 506 | South and Southeast Asia | Multifamily Residential | 2822.092773 | Heeren & Fishman |
| 507 | South and Southeast Asia | Multifamily Residential | 1.05882353 | Heeren & Fishman |
| 508 | South and Southeast Asia | Multifamily Residential | 1.05882353 | Heeren & Fishman |
| 509 | South and Southeast Asia | Single Family Residential | 32.37534483 | Heeren & Fishman |
| 510 | South and Southeast Asia | Single Family Residential | 32.37534483 | Heeren & Fishman |
| 511 | East Asia | Multifamily Residential | 354 | Heeren & Fishman |
| 512 | East Asia | Multifamily Residential | 500 | Heeren & Fishman |
| 513 | Central Europe | Commercial | 1568.396245 | Heeren & Fishman |
| 514 | East Asia | Single Family Residential | 48 | Heeren & Fishman |
| 515 | East Asia | Single Family Residential | 53 | Heeren & Fishman |
| 516 | East Asia | Single Family Residential | 52 | Heeren & Fishman |
| 517 | East Asia | Single Family Residential | 64 | Heeren & Fishman |
| 518 | East Asia | Single Family Residential | 158 | Heeren & Fishman |
| 519 | East Asia | Single Family Residential | 125 | Heeren & Fishman |
| 520 | Oceania | Single Family Residential | 999.8371296 | Heeren & Fishman |
| 521 | Oceania | Single Family Residential | 1098.408951 | Heeren & Fishman |
| 522 | Oceania | Multifamily Residential | 986.5744048 | Heeren & Fishman |
| 523 | Oceania | Multifamily Residential | 769.6585317 | Heeren & Fishman |
| 524 | Central Europe | Commercial | 1542.104411 | Heeren & Fishman |
| 525 | Oceania | Multifamily Residential | 957.3984568 | Heeren & Fishman |

| | | | | |
|---|---|---|---|---|
| 526 | Oceania | Multifamily Residential | 764.0427469 | Heeren & Fishman |
| 527 | Oceania | Single Family Residential | 754.7709251 | Heeren & Fishman |
| 528 | Oceania | Single Family Residential | 513.3161689 | Heeren & Fishman |
| 529 | Oceania | Multifamily Residential | 1638.910023 | Heeren & Fishman |
| 530 | Oceania | Single Family Residential | 679.7765625 | Heeren & Fishman |
| 531 | South and Southeast Asia | Single Family Residential | 34.04 | Heeren & Fishman |
| 532 | South and Southeast Asia | Single Family Residential | 34.04 | Heeren & Fishman |
| 533 | Central Europe | Commercial | 1987.244685 | Heeren & Fishman |
| 534 | Southern Europe | Commercial | 1369.710788 | Heeren & Fishman |
| 535 | Southern Europe | Misc Residential | 1192.631581 | Heeren & Fishman |
| 536 | Southern Europe | Commercial | 1176.534661 | Heeren & Fishman |
| 537 | Southern Europe | Commercial | 1435.93196 | Heeren & Fishman |
| 538 | Southern Europe | Commercial | 1500.057929 | Heeren & Fishman |
| 539 | Southern Europe | Commercial | 1435.343196 | Heeren & Fishman |
| 540 | Southern Europe | Commercial | 614.1571043 | Heeren & Fishman |
| 541 | Southern Europe | Commercial | 2268.601564 | Heeren & Fishman |
| 542 | Southern Europe | Industrial | 1421.217325 | Heeren & Fishman |
| 543 | Southern Europe | Industrial | 1143.249984 | Heeren & Fishman |
| 544 | South America | Single Family Residential | 2358.57 | Heeren & Fishman |
| 545 | South America | Multifamily Residential | 1011.25 | Heeren & Fishman |
| 546 | Central Europe | Misc Residential | 1474.950747 | Heeren & Fishman |
| 547 | South America | Multifamily Residential | 849.02 | Heeren & Fishman |
| 548 | South America | Multifamily Residential | 737.59 | Heeren & Fishman |
| 549 | East Asia | Misc Residential | 1540.3 | Heeren & Fishman |
| 550 | East Asia | Misc Residential | 1274.8 | Heeren & Fishman |
| 551 | East Asia | Misc Residential | 1232 | Heeren & Fishman |
| 552 | East Asia | Misc Residential | 1440.8 | Heeren & Fishman |
| 553 | East Asia | Misc Residential | 1303 | Heeren & Fishman |

| | | | | |
|---|---|---|---|---|
| 554 | East Asia | Single Family Residential | 158.77 | Heeren & Fishman |
| 555 | East Asia | Multifamily Residential | 264.77 | Heeren & Fishman |
| 556 | East Asia | Commercial | 291.4 | Heeren & Fishman |
| 557 | Central Europe | Misc Residential | 1615.622899 | Heeren & Fishman |
| 558 | East Asia | Multifamily Residential | 844.45 | Heeren & Fishman |
| 559 | East Asia | Commercial | 740.5 | Heeren & Fishman |
| 560 | East Asia | Industrial | 283.8 | Heeren & Fishman |
| 561 | East Asia | Misc Residential | 30.77412099 | Heeren & Fishman |
| 562 | East Asia | Misc Residential | 4.596404 | Heeren & Fishman |
| 563 | East Asia | Misc Residential | 1370 | Heeren & Fishman |
| 564 | East Asia | Multifamily Residential | 1419 | Heeren & Fishman |
| 565 | East Asia | Misc Residential | 1892 | Heeren & Fishman |
| 566 | East Asia | Misc Residential | 2092 | Heeren & Fishman |
| 567 | East Asia | Misc Residential | 2303 | Heeren & Fishman |
| 568 | Central Europe | Misc Residential | 1590.15916 | Heeren & Fishman |
| 569 | East Asia | Misc Residential | 1861 | Heeren & Fishman |
| 570 | East Asia | Misc Residential | 1388 | Heeren & Fishman |
| 571 | East Asia | Misc Residential | 1553 | Heeren & Fishman |
| 572 | East Asia | Misc Residential | 1843 | Heeren & Fishman |
| 573 | East Asia | Misc | 1927 | Heeren & Fishman |
| 574 | East Asia | Misc | 1588 | Heeren & Fishman |
| 575 | East Asia | Misc | 2086 | Heeren & Fishman |
| 576 | East Asia | Misc | 2050 | Heeren & Fishman |
| 577 | East Asia | Misc | 2229 | Heeren & Fishman |
| 578 | East Asia | Misc | 86.22 | Heeren & Fishman |
| 579 | North America | Single Family Residential | 669.460647 | Guven et. al |
| 580 | North America | Single Family Residential | 611.8071902 | Guven et. al |
| 581 | North America | Commercial | 1363.990918 | Guven et. al |
| 582 | North America | Multifamily Residential | 786.7609163 | Guven et. al |
| 583 | North America | Single Family Residential | 604.1712724 | Guven et. al |
| 584 | North America | Single Family Residential | 608.5744855 | Guven et. al |

| | | | | |
|---|---|---|---|---|
| 585 | North America | Single Family Residential | 696.1404612 | Guven et. al |
| 586 | North America | Single Family Residential | 630.6176106 | Guven et. al |
| 587 | North America | Single Family Residential | 491.773905 | Guven et. al |
| 588 | North America | Single Family Residential | 692.0438694 | Guven et. al |
| 589 | North America | Single Family Residential | 718.7152707 | Guven et. al |
| 590 | North America | Single Family Residential | 491.4059963 | Guven et. al |
| 591 | North America | Single Family Residential | 473.3787523 | Guven et. al |
| 592 | North America | Single Family Residential | 605.6640957 | Guven et. al |
| 593 | North America | Single Family Residential | 726.0455551 | Guven et. al |
| 594 | North America | Single Family Residential | 541.9922739 | Guven et. al |
| 595 | North America | Multifamily Residential | 560.7144378 | Guven et. al |
| 596 | North America | Single Family Residential | 500.4501086 | Guven et. al |
| 597 | North America | Single Family Residential | 509.9630412 | Guven et. al |
| 598 | North America | Commercial | 1246.157466 | Guven et. al |
| 599 | North America | Single Family Residential | 396.9998446 | Guven et. al |
| 600 | North America | Single Family Residential | 492.5143313 | Guven et. al |
| 601 | North America | Single Family Residential | 741.3181664 | Guven et. al |
| 602 | North America | Multifamily Residential | 530.7391659 | Guven et. al |
| 603 | North America | Single Family Residential | 778.9233958 | Guven et. al |
| 604 | North America | Single Family Residential | 709.730812 | Guven et. al |
| 605 | North America | Single Family Residential | 527.817611 | Guven et. al |

| | | | | |
|---|---|---|---|---|
| 606 | North America | Multifamily Residential | 562.751545 | Guven et. al |
| 607 | North America | Single Family Residential | 537.3970641 | Guven et. al |
| 608 | North America | Single Family Residential | 706.2647184 | Guven et. al |
| 609 | North America | Single Family Residential | 758.4054176 | Guven et. al |
| 610 | North America | Single Family Residential | 699.5946324 | Guven et. al |
| 611 | North America | Single Family Residential | 693.4203354 | Guven et. al |
| 612 | North America | Single Family Residential | 547.8798783 | Guven et. al |
| 613 | North America | Institutional | 599.4601057 | Guven et. al |
| 614 | North America | Single Family Residential | 567.4372508 | Guven et. al |
| 615 | North America | Single Family Residential | 535.5526446 | Guven et. al |
| 616 | North America | Single Family Residential | 591.4872698 | Guven et. al |
| 617 | North America | Single Family Residential | 788.6805807 | Guven et. al |
| 618 | North America | Single Family Residential | 513.1772823 | Guven et. al |
| 619 | North America | Single Family Residential | 716.3855952 | Guven et. al |
| 620 | North America | Single Family Residential | 522.3680524 | Guven et. al |
| 621 | North America | Institutional | 1035.411291 | Guven et. al |
| 622 | North America | Single Family Residential | 499.9495709 | Guven et. al |
| 623 | North America | Commercial | 1146.810103 | Guven et. al |
| 624 | North America | Single Family Residential | 677.1645324 | Guven et. al |
| 625 | North America | Institutional | 1294.999893 | Guven et. al |
| 626 | North America | Multifamily Residential | 1261.915614 | Guven et. al |
| 627 | North America | Commercial | 1331.228073 | Guven et. al |

| | | | | |
|---|---|---|---|---|
| 628 | North America | Multifamily Residential | 1081.238084 | Guven et. al |
| 629 | North America | Multifamily Residential | 1142.621037 | Guven et. al |
| 630 | North America | Multifamily Residential | 1339.025403 | Guven et. al |
| 631 | North America | Single Family Residential | 509.8286818 | Guven et. al |
| 632 | North America | Single Family Residential | 501.7756003 | Guven et. al |
| 633 | North America | Single Family Residential | 565.1129964 | Guven et. al |
| 634 | North America | Multifamily Residential | 1299.132154 | Guven et. al |
| 635 | North America | Single Family Residential | 586.0559223 | Guven et. al |
| 636 | North America | Multifamily Residential | 985.2593658 | Guven et. al |
| 637 | North America | Commercial | 1180.494615 | Guven et. al |
| 638 | North America | Multifamily Residential | 1022.158384 | Guven et. al |
| 639 | North America | Multifamily Residential | 1018.962101 | Guven et. al |
| 640 | North America | Multifamily Residential | 230.2254991 | Guven et. al |
| 641 | North America | Commercial | 1201.09003 | Guven et. al |
| 642 | North America | Single Family Residential | 554.2629771 | Guven et. al |
| 643 | North America | Single Family Residential | 908.6743662 | Guven et. al |
| 644 | North America | Single Family Residential | 633.4741837 | Guven et. al |
| 645 | North America | Single Family Residential | 518.4187762 | Guven et. al |
| 646 | North America | Single Family Residential | 413.1296183 | Guven et. al |
| 647 | North America | Single Family Residential | 574.2443609 | Guven et. al |
| 648 | North America | Single Family Residential | 608.9657176 | Guven et. al |

| | | | | |
|---|---|---|---|---|
| 649 | Southern Europe | Multifamily Residential | 1978.90 | Augiseau V, Kim E. |
| 650 | Southern Europe | Multifamily Residential | 1783.20 | Augiseau V, Kim E. |
| 651 | Southern Europe | Multifamily Residential | 1955.40 | Augiseau V, Kim E. |
| 652 | Southern Europe | Multifamily Residential | 1857.50 | Augiseau V, Kim E. |
| 653 | Southern Europe | Multifamily Residential | 1738.00 | Augiseau V, Kim E. |
| 654 | Southern Europe | Multifamily Residential | 1412.80 | Augiseau V, Kim E. |
| 655 | Southern Europe | Multifamily Residential | 1693.00 | Augiseau V, Kim E. |
| 656 | Southern Europe | Multifamily Residential | 1658.90 | Augiseau V, Kim E. |
| 657 | Southern Europe | Single Family Residential | 1858.80 | Augiseau V, Kim E. |
| 658 | Southern Europe | Single Family Residential | 1858.80 | Augiseau V, Kim E. |
| 659 | Southern Europe | Single Family Residential | 1093.80 | Augiseau V, Kim E. |
| 660 | Southern Europe | Single Family Residential | 1045.20 | Augiseau V, Kim E. |
| 661 | Southern Europe | Single Family Residential | 896.00 | Augiseau V, Kim E. |
| 662 | Southern Europe | Single Family Residential | 1398.90 | Augiseau V, Kim E. |
| 663 | Southern Europe | Single Family Residential | 1714.80 | Augiseau V, Kim E. |
| 664 | Southern Europe | Single Family Residential | 656.20 | Augiseau V, Kim E. |
| 665 | Southern Europe | Commercial | 483.80 | Augiseau V, Kim E. |
| 666 | Southern Europe | Commercial | 1957.80 | Augiseau V, Kim E. |
| 667 | Southern Europe | Commercial | 1565.00 | Augiseau V, Kim E. |
| 668 | Southern Europe | Commercial | 1727.60 | Augiseau V, Kim E. |
| 669 | Southern Europe | Commercial | 1412.80 | Augiseau V, Kim E. |
| 670 | Southern Europe | Commercial | 1505.70 | Augiseau V, Kim E. |
| 671 | Southern Europe | Industrial | 851.50 | Augiseau V, Kim E. |
| 672 | Southern Europe | Industrial | 522.10 | Augiseau V, Kim E. |
| 673 | Northern Europe | Single Family Residential | 574.32 | Bergsdal H.,Ã‚Â Bohne, R.A. &Ã‚Â BrattebÃƒÂ¸, H.Ã‚Â |
| 674 | Northern Europe | Commercial | 1102.84 | Bergsdal H.,Ã‚Â Bohne, R.A. &Ã‚Â BrattebÃƒÂ¸, H.Ã‚Â |
| 675 | Northern Europe | Industrial | 601.94 | Bergsdal H.,Ã‚Â Bohne, R.A. &Ã‚Â BrattebÃƒÂ¸, H.Ã‚Â |

| | | | | |
|---|---|---|---|---|
| 676 | North America | Single Family Residential | 616.18 | Berril, P. & Herwitch, EG |
| 677 | North America | Single Family Residential | 526.65 | Berril, P. & Herwitch, EG |
| 678 | North America | Single Family Residential | 773.69 | Berril, P. & Herwitch, EG |
| 679 | North America | Single Family Residential | 616.43 | Berril, P. & Herwitch, EG |
| 680 | North America | Single Family Residential | 458.32 | Berril, P. & Herwitch, EG |
| 681 | North America | Single Family Residential | 377.82 | Berril, P. & Herwitch, EG |
| 682 | North America | Single Family Residential | 463.53 | Berril, P. & Herwitch, EG |
| 683 | North America | Single Family Residential | 910.49 | Berril, P. & Herwitch, EG |
| 684 | North America | Single Family Residential | 760.17 | Berril, P. & Herwitch, EG |
| 685 | North America | Single Family Residential | 1231.78 | Berril, P. & Herwitch, EG |
| 686 | North America | Single Family Residential | 957.03 | Berril, P. & Herwitch, EG |
| 687 | North America | Single Family Residential | 817.83 | Berril, P. & Herwitch, EG |
| 688 | North America | Single Family Residential | 680.81 | Berril, P. & Herwitch, EG |
| 689 | North America | Single Family Residential | 906.86 | Berril, P. & Herwitch, EG |
| 690 | North America | Single Family Residential | 308.29 | Berril, P. & Herwitch, EG |
| 691 | North America | Single Family Residential | 347.83 | Berril, P. & Herwitch, EG |
| 692 | North America | Single Family Residential | 708.12 | Berril, P. & Herwitch, EG |
| 693 | North America | Single Family Residential | 618.59 | Berril, P. & Herwitch, EG |
| 694 | North America | Single Family Residential | 954.56 | Berril, P. & Herwitch, EG |
| 695 | North America | Single Family Residential | 797.29 | Berril, P. & Herwitch, EG |
| 696 | North America | Single Family Residential | 532.98 | Berril, P. & Herwitch, EG |
| 697 | North America | Single Family Residential | 452.49 | Berril, P. & Herwitch, EG |
| 698 | North America | Single Family Residential | 606.41 | Berril, P. & Herwitch, EG |

| | | | | |
|---|---|---|---|---|
| 699 | North America | Single Family Residential | 1002.43 | Berril, P. & Herwitch, EG |
| 700 | North America | Single Family Residential | 852.11 | Berril, P. & Herwitch, EG |
| 701 | North America | Single Family Residential | 1412.64 | Berril, P. & Herwitch, EG |
| 702 | North America | Single Family Residential | 1137.90 | Berril, P. & Herwitch, EG |
| 703 | North America | Single Family Residential | 892.45 | Berril, P. & Herwitch, EG |
| 704 | North America | Single Family Residential | 755.43 | Berril, P. & Herwitch, EG |
| 705 | North America | Single Family Residential | 1049.74 | Berril, P. & Herwitch, EG |
| 706 | North America | Single Family Residential | 426.98 | Berril, P. & Herwitch, EG |
| 707 | North America | Single Family Residential | 337.46 | Berril, P. & Herwitch, EG |
| 708 | North America | Single Family Residential | 601.24 | Berril, P. & Herwitch, EG |
| 709 | North America | Single Family Residential | 443.98 | Berril, P. & Herwitch, EG |
| 710 | North America | Single Family Residential | 361.99 | Berril, P. & Herwitch, EG |
| 711 | North America | Single Family Residential | 301.56 | Berril, P. & Herwitch, EG |
| 712 | North America | Single Family Residential | 361.15 | Berril, P. & Herwitch, EG |
| 713 | North America | Single Family Residential | 721.45 | Berril, P. & Herwitch, EG |
| 714 | North America | Single Family Residential | 571.13 | Berril, P. & Herwitch, EG |
| 715 | North America | Single Family Residential | 1059.50 | Berril, P. & Herwitch, EG |
| 716 | North America | Single Family Residential | 784.76 | Berril, P. & Herwitch, EG |
| 717 | North America | Single Family Residential | 721.70 | Berril, P. & Herwitch, EG |
| 718 | North America | Single Family Residential | 584.68 | Berril, P. & Herwitch, EG |
| 719 | North America | Single Family Residential | 804.78 | Berril, P. & Herwitch, EG |
| 720 | North America | Single Family Residential | 255.44 | Berril, P. & Herwitch, EG |
| 721 | North America | Single Family Residential | 279.53 | Berril, P. & Herwitch, EG |

| | | | | |
|---|---|---|---|---|
| 722 | North America | Single Family Residential | 195.70 | Berril, P. & Herwitch, EG |
| 723 | North America | Single Family Residential | 264.00 | Berril, P. & Herwitch, EG |
| 724 | North America | Single Family Residential | 164.52 | Berril, P. & Herwitch, EG |
| 725 | North America | Single Family Residential | 210.19 | Berril, P. & Herwitch, EG |
| 726 | North America | Single Family Residential | 994.49 | Berril, P. & Herwitch, EG |
| 727 | East Asia | Misc Residential | 1676.40 | Cha GW, Moon HJ, Kim YC, Hong WH, Jeon GY, Yoon YR, Hwang C, Hwang JH |
| 728 | East Asia | Misc Residential | 1538.10 | Cha GW, Moon HJ, Kim YC, Hong WH, Jeon GY, Yoon YR, Hwang C, Hwang JH |
| 729 | East Asia | Misc Residential | 1135.50 | Cha GW, Moon HJ, Kim YC, Hong WH, Jeon GY, Yoon YR, Hwang C, Hwang JH |
| 730 | East Asia | Misc Residential | 908.00 | Cha GW, Moon HJ, Kim YC, Hong WH, Jeon GY, Yoon YR, Hwang C, Hwang JH |
| 731 | East Asia | Misc | 1733.59 | Cheng, Kuang Ly; Hsu, Shu Chien; Li, Wing Man; Ma, Hwong Wen |
| 732 | North America | Multifamily Residential | 600.00 | Cochran, K., Townsend, T., Reinhart, D. & Heck, H |
| 733 | North America | Single Family Residential | 910.00 | Cochran, K., Townsend, T., Reinhart, D. & Heck, H |
| 734 | North America | Single Family Residential | 195.00 | Cochran, K., Townsend, T., Reinhart, D. & Heck, H |
| 735 | North America | Single Family Residential | 435.00 | Cochran, K., Townsend, T., Reinhart, D. & Heck, H |
| 736 | North America | Single Family Residential | 665.00 | Cochran, K., Townsend, T., Reinhart, D. & Heck, H |
| 737 | North America | Commercial | 845.50 | Cochran, K., Townsend, T., Reinhart, D. & Heck, H |
| 738 | Oceania | Multifamily Residential | 1366.01 | Durlinger B., Crossin E., Wong J. |
| 739 | Oceania | Multifamily Residential | 2609.43 | Durlinger B., Crossin E., Wong J. |
| 740 | East Asia | Multifamily Residential | 1163.80 | Gao X, Nakatani J, Zhang Q, Huang B, Wang T, Moriguchi Y. |
| 741 | East Asia | Multifamily Residential | 1160.70 | Gao X, Nakatani J, Zhang Q, Huang B, Wang T, Moriguchi Y. |

| | | | | |
|---|---|---|---|---|
| 742 | East Asia | Multifamily Residential | 1203.10 | Gao X, Nakatani J, Zhang Q, Huang B, Wang T, Moriguchi Y. |
| 743 | East Asia | Multifamily Residential | 1467.00 | Gao X, Nakatani J, Zhang Q, Huang B, Wang T, Moriguchi Y. |
| 744 | East Asia | Multifamily Residential | 1589.90 | Gao X, Nakatani J, Zhang Q, Huang B, Wang T, Moriguchi Y. |
| 745 | East Asia | Multifamily Residential | 1091.70 | Gao X, Nakatani J, Zhang Q, Huang B, Wang T, Moriguchi Y. |
| 746 | East Asia | Multifamily Residential | 1102.60 | Gao X, Nakatani J, Zhang Q, Huang B, Wang T, Moriguchi Y. |
| 747 | East Asia | Multifamily Residential | 1326.30 | Gao X, Nakatani J, Zhang Q, Huang B, Wang T, Moriguchi Y. |
| 748 | East Asia | Multifamily Residential | 1456.20 | Gao X, Nakatani J, Zhang Q, Huang B, Wang T, Moriguchi Y. |
| 749 | East Asia | Single Family Residential | 1576.70 | Gao X, Nakatani J, Zhang Q, Huang B, Wang T, Moriguchi Y. |
| 750 | East Asia | Single Family Residential | 86.22 | Gao X, Nakatani J, Zhang Q, Huang B, Wang T, Moriguchi Y. |
| 751 | East Asia | Single Family Residential | 86.22 | Gao X, Nakatani J, Zhang Q, Huang B, Wang T, Moriguchi Y. |
| 752 | East Asia | Single Family Residential | 935.95 | Gao X, Nakatani J, Zhang Q, Huang B, Wang T, Moriguchi Y. |
| 753 | East Asia | Single Family Residential | 1203.10 | Gao X, Nakatani J, Zhang Q, Huang B, Wang T, Moriguchi Y. |
| 754 | East Asia | Single Family Residential | 1467.00 | Gao X, Nakatani J, Zhang Q, Huang B, Wang T, Moriguchi Y. |
| 755 | East Asia | Single Family Residential | 1589.90 | Gao X, Nakatani J, Zhang Q, Huang B, Wang T, Moriguchi Y. |
| 756 | East Asia | Single Family Residential | 1326.30 | Gao X, Nakatani J, Zhang Q, Huang B, Wang T, Moriguchi Y. |

| | | | | |
|---|---|---|---|---|
| 757 | East Asia | Single Family Residential | 1430.20 | Gao X, Nakatani J, Zhang Q, Huang B, Wang T, Moriguchi Y. |
| 758 | East Asia | Single Family Residential | 1580.70 | Gao X, Nakatani J, Zhang Q, Huang B, Wang T, Moriguchi Y. |
| 759 | Northern Europe | Single Family Residential | 1108.00 | Gontia, P., Thuvander, L. & Wallbaum, H |
| 760 | Northern Europe | Single Family Residential | 870.00 | Gontia, P., Thuvander, L. & Wallbaum, H |
| 761 | Northern Europe | Single Family Residential | 773.00 | Gontia, P., Thuvander, L. & Wallbaum, H |
| 762 | Northern Europe | Single Family Residential | 603.00 | Gontia, P., Thuvander, L. & Wallbaum, H |
| 763 | Northern Europe | Multifamily Residential | 890.00 | Gontia, P., Thuvander, L. & Wallbaum, H |
| 764 | Northern Europe | Multifamily Residential | 1108.00 | Gontia, P., Thuvander, L. & Wallbaum, H |
| 765 | Northern Europe | Multifamily Residential | 1207.00 | Gontia, P., Thuvander, L. & Wallbaum, H |
| 766 | Northern Europe | Multifamily Residential | 1235.00 | Gontia, P., Thuvander, L. & Wallbaum, H |
| 767 | Northern Europe | Multifamily Residential | 1139.00 | Gontia, P., Thuvander, L., Ebrahimi, B., Vinas, V., Rosado, L. & Wallbaum, H |
| 768 | Northern Europe | Multifamily Residential | 1049.00 | Gontia, P., Thuvander, L., Ebrahimi, B., Vinas, V., Rosado, L. & Wallbaum, H |
| 769 | Northern Europe | Multifamily Residential | 970.00 | Gontia, P., Thuvander, L., Ebrahimi, B., Vinas, V., Rosado, L. & Wallbaum, H |
| 770 | Northern Europe | Multifamily Residential | 965.00 | Gontia, P., Thuvander, L., Ebrahimi, B., Vinas, V., Rosado, L. & Wallbaum, H |
| 771 | Northern Europe | Multifamily Residential | 895.00 | Gontia, P., Thuvander, L., Ebrahimi, B., Vinas, V., Rosado, L. & Wallbaum, H |
| 772 | Northern Europe | Multifamily Residential | 541.00 | Gontia, P., Thuvander, L., Ebrahimi, B., Vinas, V., Rosado, L. & Wallbaum, H |
| 773 | Northern Europe | Multifamily Residential | 567.00 | Gontia, P., Thuvander, L., Ebrahimi, B., Vinas, V., Rosado, L. & Wallbaum, H |
| 774 | Northern Europe | Multifamily Residential | 416.00 | Gontia, P., Thuvander, L., Ebrahimi, B., Vinas, V., Rosado, L. & Wallbaum, H |

| | | | | |
|---|---|---|---|---|
| 775 | Northern Europe | Commercial | 1350.00 | Gontia, P., Thuvander, L., Ebrahimi, B., Vinas, V., Rosado, L. & Wallbaum, H |
| 776 | Northern Europe | Commercial | 1267.00 | Gontia, P., Thuvander, L., Ebrahimi, B., Vinas, V., Rosado, L. & Wallbaum, H |
| 777 | Northern Europe | Commercial | 983.00 | Gontia, P., Thuvander, L., Ebrahimi, B., Vinas, V., Rosado, L. & Wallbaum, H |
| 778 | Northern Europe | Industrial | 2403.00 | Gontia, P., Thuvander, L., Ebrahimi, B., Vinas, V., Rosado, L. & Wallbaum, H |
| 779 | Northern Europe | Industrial | 921.00 | Gontia, P., Thuvander, L., Ebrahimi, B., Vinas, V., Rosado, L. & Wallbaum, H |
| 780 | Northern Europe | Industrial | 965.00 | Gontia, P., Thuvander, L., Ebrahimi, B., Vinas, V., Rosado, L. & Wallbaum, H |
| 781 | Northern Europe | Institutional | 1600.00 | Gontia, P., Thuvander, L., Ebrahimi, B., Vinas, V., Rosado, L. & Wallbaum, H |
| 782 | Northern Europe | Institutional | 1140.00 | Gontia, P., Thuvander, L., Ebrahimi, B., Vinas, V., Rosado, L. & Wallbaum, H |
| 783 | Northern Europe | Institutional | 968.00 | Gontia, P., Thuvander, L., Ebrahimi, B., Vinas, V., Rosado, L. & Wallbaum, H |
| 784 | East Asia | Misc Residential | 1633.90 | Han J, Chen WQ, Zhang L, Liu G. |
| 785 | East Asia | Misc Residential | 1498.80 | Han J, Chen WQ, Zhang L, Liu G. |
| 786 | East Asia | Misc Residential | 1393.00 | Han J, Chen WQ, Zhang L, Liu G. |
| 787 | East Asia | Misc Residential | 1307.10 | Han J, Chen WQ, Zhang L, Liu G. |
| 788 | East Asia | Misc Residential | 1269.00 | Han J, Chen WQ, Zhang L, Liu G. |
| 789 | East Asia | Misc Residential | 1212.90 | Han J, Chen WQ, Zhang L, Liu G. |
| 790 | East Asia | Misc Residential | 1146.10 | Han J, Chen WQ, Zhang L, Liu G. |
| 791 | East Asia | Misc Residential | 1126.20 | Han J, Chen WQ, Zhang L, Liu G. |
| 792 | East Asia | Multifamily Residential | 86.22 | Han J, Chen WQ, Zhang L, Liu G. |
| 793 | East Asia | Multifamily Residential | 935.95 | Han J, Chen WQ, Zhang L, Liu G. |

| | | | | |
|---|---|---|---|---|
| 794 | East Asia | Commercial | 1563.37 | Hui, Y., Qinping, S., Fan, L.C.H., Yoawu, W. & Lei, Z |
| 795 | Oceania | Multifamily Residential | 2658.51 | Jayalath, A.; Navaratnam, S.; Ngo, T.; Mendis, P.; Hewson, N.; Aye, L. |
| 796 | Oceania | Multifamily Residential | 1026.82 | Jayalath, A.; Navaratnam, S.; Ngo, T.; Mendis, P.; Hewson, N.; Aye, L. |
| 797 | Northern Europe | Institutional | 728.92 | Lausselet, C., Urrego, J.P.F., Resch, E. &Ã‚Â BrattebÃƒÂ¸, H |
| 798 | Northern Europe | Institutional | 995.87 | Lausselet, C., Urrego, J.P.F., Resch, E. &Ã‚Â BrattebÃƒÂ¸, H |
| 799 | Northern Europe | Single Family Residential | 694.86 | Lausselet, C., Urrego, J.P.F., Resch, E. &Ã‚Â BrattebÃƒÂ¸, H |
| 800 | Northern Europe | Single Family Residential | 694.86 | Lausselet, C., Urrego, J.P.F., Resch, E. &Ã‚Â BrattebÃƒÂ¸, H |
| 801 | Northern Europe | Single Family Residential | 694.86 | Lausselet, C., Urrego, J.P.F., Resch, E. &Ã‚Â BrattebÃƒÂ¸, H |
| 802 | East Asia | Misc Residential | 191.18 | Li H, Luo Z, Xu X, Cang Y, Yang L. |
| 803 | East Asia | Misc Residential | 84.07 | Li H, Luo Z, Xu X, Cang Y, Yang L. |
| 804 | East Asia | Misc Residential | 42.68 | Li H, Luo Z, Xu X, Cang Y, Yang L. |
| 805 | East Asia | Misc Residential | 109.76 | Li H, Luo Z, Xu X, Cang Y, Yang L. |
| 806 | East Asia | Multifamily Residential | 529.26 | Li H, Luo Z, Xu X, Cang Y, Yang L. |
| 807 | Southern Europe | Single Family Residential | 549.80 | Malia, M., de Brito, J., Pinheiro, M.D. & Bravo, M. |
| 808 | Southern Europe | Single Family Residential | 1081.70 | Malia, M., de Brito, J., Pinheiro, M.D. & Bravo, M. |
| 809 | Southern Europe | Single Family Residential | 552.45 | Malia, M., de Brito, J., Pinheiro, M.D. & Bravo, M. |
| 810 | Southern Europe | Misc | 382.35 | Malia, M., de Brito, J., Pinheiro, M.D. & Bravo, M. |
| 811 | Southern Europe | Misc Residential | 717.25 | Malia, M., de Brito, J., Pinheiro, M.D. & Bravo, M. |
| 812 | Southern Europe | Misc Residential | 684.00 | Malia, M., de Brito, J., Pinheiro, M.D. & Bravo, M. |
| 813 | East Asia | Institutional | 490.00 | Mao R, Bao Y, Huang Z, Liu Q, Liu G |

| | | | | |
|---|---|---|---|---|
| 814 | East Asia | Commercial | 2541.05 | Mao R, Bao Y, Huang Z, Liu Q, Liu G |
| 815 | East Asia | Institutional | 2176.39 | Mao R, Bao Y, Huang Z, Liu Q, Liu G |
| 816 | East Asia | Industrial | 460.88 | Mao R, Bao Y, Huang Z, Liu Q, Liu G |
| 817 | East Asia | Commercial | 1767.56 | Mao R, Bao Y, Huang Z, Liu Q, Liu G |
| 818 | East Asia | Industrial | 1741.81 | Mao R, Bao Y, Huang Z, Liu Q, Liu G |
| 819 | East Asia | Institutional | 2307.78 | Mao R, Bao Y, Huang Z, Liu Q, Liu G |
| 820 | East Asia | Institutional | 2485.69 | Mao R, Bao Y, Huang Z, Liu Q, Liu G |
| 821 | East Asia | Misc Residential | 1457.00 | Mao R, Bao Y, Huang Z, Liu Q, Liu G |
| 822 | East Asia | Misc | 2424.51 | Mao R, Bao Y, Huang Z, Liu Q, Liu G |
| 823 | East Asia | Misc | 912.80 | Mao R, Bao Y, Huang Z, Liu Q, Liu G |
| 824 | East Asia | Misc | 1039.44 | Mao R, Bao Y, Huang Z, Liu Q, Liu G |
| 825 | East Asia | Commercial | 2108.98 | Mao R, Bao Y, Huang Z, Liu Q, Liu G |
| 826 | East Asia | Institutional | 1675.21 | Mao R, Bao Y, Huang Z, Liu Q, Liu G |
| 827 | East Asia | Industrial | 518.95 | Mao R, Bao Y, Huang Z, Liu Q, Liu G |
| 828 | East Asia | Commercial | 2017.54 | Mao R, Bao Y, Huang Z, Liu Q, Liu G |
| 829 | East Asia | Industrial | 1389.54 | Mao R, Bao Y, Huang Z, Liu Q, Liu G |
| 830 | East Asia | Institutional | 1987.76 | Mao R, Bao Y, Huang Z, Liu Q, Liu G |
| 831 | East Asia | Institutional | 2019.15 | Mao R, Bao Y, Huang Z, Liu Q, Liu G |
| 832 | East Asia | Misc Residential | 1157.50 | Mao R, Bao Y, Huang Z, Liu Q, Liu G |
| 833 | East Asia | Misc | 2607.16 | Mao R, Bao Y, Huang Z, Liu Q, Liu G |
| 834 | East Asia | Misc | 1054.50 | Mao R, Bao Y, Huang Z, Liu Q, Liu G |
| 835 | East Asia | Misc | 1371.43 | Mao R, Bao Y, Huang Z, Liu Q, Liu G |
| 836 | East Asia | Commercial | 1263.91 | Mao R, Bao Y, Huang Z, Liu Q, Liu G |

| | | | | |
|---|---|---|---|---|
| 837 | East Asia | Institutional | 1000.42 | Mao R, Bao Y, Huang Z, Liu Q, Liu G |
| 838 | East Asia | Industrial | 645.90 | Mao R, Bao Y, Huang Z, Liu Q, Liu G |
| 839 | East Asia | Commercial | 506.03 | Mao R, Bao Y, Huang Z, Liu Q, Liu G |
| 840 | East Asia | Industrial | 750.83 | Mao R, Bao Y, Huang Z, Liu Q, Liu G |
| 841 | East Asia | Institutional | 1415.19 | Mao R, Bao Y, Huang Z, Liu Q, Liu G |
| 842 | East Asia | Institutional | 1518.00 | Mao R, Bao Y, Huang Z, Liu Q, Liu G |
| 843 | East Asia | Misc Residential | 846.59 | Mao R, Bao Y, Huang Z, Liu Q, Liu G |
| 844 | East Asia | Institutional | 1355.19 | Mao R, Bao Y, Huang Z, Liu Q, Liu G |
| 845 | East Asia | Misc | 2909.95 | Mao R, Bao Y, Huang Z, Liu Q, Liu G |
| 846 | East Asia | Misc | 899.34 | Mao R, Bao Y, Huang Z, Liu Q, Liu G |
| 847 | East Asia | Misc | 453.76 | Mao R, Bao Y, Huang Z, Liu Q, Liu G |
| 848 | East Asia | Misc | 247.12 | Mao R, Bao Y, Huang Z, Liu Q, Liu G |
| 849 | East Asia | Misc | 541.52 | Mao R, Bao Y, Huang Z, Liu Q, Liu G |
| 850 | Oceania | Misc | 1830.00 | Merschroth S, Miatto A, Weyand S, Tanikawa H, Schebek L. |
| 851 | Oceania | Misc | 1520.00 | Merschroth S, Miatto A, Weyand S, Tanikawa H, Schebek L. |
| 852 | Oceania | Misc | 177.00 | Merschroth S, Miatto A, Weyand S, Tanikawa H, Schebek L. |
| 853 | Oceania | Misc | 207.00 | Merschroth S, Miatto A, Weyand S, Tanikawa H, Schebek L. |
| 854 | South America | Misc Residential | 1606.80 | Mesta, Carlos; Kahhat, Ramzy; Santa-Cruz, Sandra |
| 855 | South America | Misc Residential | 689.00 | Mesta, Carlos; Kahhat, Ramzy; Santa-Cruz, Sandra |
| 856 | South America | Misc Residential | 1445.50 | Mesta, Carlos; Kahhat, Ramzy; Santa-Cruz, Sandra |
| 857 | Central Europe | Multifamily Residential | 805.54 | Mitterpach, J., HroncovÃƒÂ¡, E., |

| | | | | |
|---|---|---|---|---|
| | | | | LadomerskÃƒÂ½, J. & Ã…Â tefko, J |
| 858 | North America | Single Family Residential | 755.00 | Mollaei A, Ibrahim N, Habib K. |
| 859 | North America | Single Family Residential | 936.00 | Mollaei A, Ibrahim N, Habib K. |
| 860 | North America | Single Family Residential | 888.00 | Mollaei A, Ibrahim N, Habib K. |
| 861 | North America | Single Family Residential | 1123.81 | Mollaei A, Ibrahim N, Habib K. |
| 862 | North America | Single Family Residential | 1616.00 | Mollaei A, Ibrahim N, Habib K. |
| 863 | North America | Multifamily Residential | 1581.00 | Mollaei A, Ibrahim N, Habib K. |
| 864 | North America | Multifamily Residential | 809.00 | Mollaei A, Ibrahim N, Habib K. |
| 865 | North America | Multifamily Residential | 465.00 | Mollaei A, Ibrahim N, Habib K. |
| 866 | North America | Commercial | 587.00 | Mollaei A, Ibrahim N, Habib K. |
| 867 | North America | Commercial | 500.00 | Mollaei A, Ibrahim N, Habib K. |
| 868 | North America | Industrial | 730.00 | Mollaei A, Ibrahim N, Habib K. |
| 869 | North America | Institutional | 702.00 | Mollaei A, Ibrahim N, Habib K. |
| 870 | North America | Misc | 703.00 | Mollaei A, Ibrahim N, Habib K. |
| 871 | North America | Institutional | 880.00 | Mollaei A, Ibrahim N, Habib K. |
| 872 | North America | Commercial | 500.00 | Mollaei A, Ibrahim N, Habib K. |
| 873 | Southern Europe | Single Family Residential | 2507.31 | Nemry, F., & Uihlein, A. |
| 874 | Southern Europe | Single Family Residential | 2739.87 | Nemry, F., & Uihlein, A. |
| 875 | Southern Europe | Single Family Residential | 1716.12 | Nemry, F., & Uihlein, A. |
| 876 | Southern Europe | Single Family Residential | 2254.71 | Nemry, F., & Uihlein, A. |
| 877 | Southern Europe | Single Family Residential | 2439.91 | Nemry, F., & Uihlein, A. |
| 878 | Southern Europe | Single Family Residential | 2450.46 | Nemry, F., & Uihlein, A. |
| 879 | Southern Europe | Single Family Residential | 2665.39 | Nemry, F., & Uihlein, A. |

| | | | | |
|---|---|---|---|---|
| 880 | Southern Europe | Single Family Residential | 2675.35 | Nemry, F., & Uihlein, A. |
| 881 | Southern Europe | Single Family Residential | 2449.99 | Nemry, F., & Uihlein, A. |
| 882 | Southern Europe | Single Family Residential | 2448.09 | Nemry, F., & Uihlein, A. |
| 883 | Southern Europe | Single Family Residential | 2022.79 | Nemry, F., & Uihlein, A. |
| 884 | Southern Europe | Multifamily Residential | 1767.16 | Nemry, F., & Uihlein, A. |
| 885 | Southern Europe | Multifamily Residential | 1928.23 | Nemry, F., & Uihlein, A. |
| 886 | Southern Europe | Multifamily Residential | 1352.39 | Nemry, F., & Uihlein, A. |
| 887 | Southern Europe | Multifamily Residential | 1042.58 | Nemry, F., & Uihlein, A. |
| 888 | Southern Europe | Multifamily Residential | 1041.55 | Nemry, F., & Uihlein, A. |
| 889 | Southern Europe | Multifamily Residential | 1479.54 | Nemry, F., & Uihlein, A. |
| 890 | Southern Europe | Multifamily Residential | 1439.26 | Nemry, F., & Uihlein, A. |
| 891 | Southern Europe | Multifamily Residential | 1440.70 | Nemry, F., & Uihlein, A. |
| 892 | Southern Europe | Multifamily Residential | 1466.25 | Nemry, F., & Uihlein, A. |
| 893 | Southern Europe | Multifamily Residential | 1263.58 | Nemry, F., & Uihlein, A. |
| 894 | Southern Europe | Multifamily Residential | 1264.72 | Nemry, F., & Uihlein, A. |
| 895 | Southern Europe | Multifamily Residential | 1044.71 | Nemry, F., & Uihlein, A. |
| 896 | Southern Europe | Multifamily Residential | 1053.93 | Nemry, F., & Uihlein, A. |
| 897 | Southern Europe | Multifamily Residential | 1170.85 | Nemry, F., & Uihlein, A. |
| 898 | Central Europe | Single Family Residential | 2514.82 | Nemry, F., & Uihlein, A. |
| 899 | Central Europe | Single Family Residential | 2287.60 | Nemry, F., & Uihlein, A. |
| 900 | Central Europe | Single Family Residential | 3034.97 | Nemry, F., & Uihlein, A. |
| 901 | Central Europe | Single Family Residential | 2343.32 | Nemry, F., & Uihlein, A. |
| 902 | Central Europe | Single Family Residential | 2591.55 | Nemry, F., & Uihlein, A. |

| | | | | |
|---|---|---|---|---|
| 903 | Central Europe | Single Family Residential | 2605.96 | Nemry, F., & Uihlein, A. |
| 904 | Central Europe | Single Family Residential | 2613.54 | Nemry, F., & Uihlein, A. |
| 905 | Central Europe | Single Family Residential | 2508.18 | Nemry, F., & Uihlein, A. |
| 906 | Central Europe | Single Family Residential | 2515.76 | Nemry, F., & Uihlein, A. |
| 907 | Central Europe | Single Family Residential | 1283.32 | Nemry, F., & Uihlein, A. |
| 908 | Central Europe | Single Family Residential | 1280.89 | Nemry, F., & Uihlein, A. |
| 909 | Central Europe | Multifamily Residential | 1927.58 | Nemry, F., & Uihlein, A. |
| 910 | Central Europe | Multifamily Residential | 1718.60 | Nemry, F., & Uihlein, A. |
| 911 | Central Europe | Multifamily Residential | 1262.90 | Nemry, F., & Uihlein, A. |
| 912 | Central Europe | Multifamily Residential | 1287.56 | Nemry, F., & Uihlein, A. |
| 913 | Central Europe | Multifamily Residential | 1265.42 | Nemry, F., & Uihlein, A. |
| 914 | Central Europe | Multifamily Residential | 1270.25 | Nemry, F., & Uihlein, A. |
| 915 | Central Europe | Multifamily Residential | 1207.31 | Nemry, F., & Uihlein, A. |
| 916 | Central Europe | Multifamily Residential | 1207.70 | Nemry, F., & Uihlein, A. |
| 917 | Central Europe | Multifamily Residential | 1593.42 | Nemry, F., & Uihlein, A. |
| 918 | Central Europe | Multifamily Residential | 1596.72 | Nemry, F., & Uihlein, A. |
| 919 | Central Europe | Multifamily Residential | 1513.63 | Nemry, F., & Uihlein, A. |
| 920 | Central Europe | Multifamily Residential | 1108.18 | Nemry, F., & Uihlein, A. |
| 921 | Central Europe | Multifamily Residential | 988.71 | Nemry, F., & Uihlein, A. |
| 922 | Central Europe | Multifamily Residential | 985.92 | Nemry, F., & Uihlein, A. |
| 923 | Northern Europe | Single Family Residential | 2516.70 | Nemry, F., & Uihlein, A. |
| 924 | Northern Europe | Single Family Residential | 2745.17 | Nemry, F., & Uihlein, A. |
| 925 | Northern Europe | Single Family Residential | 1722.12 | Nemry, F., & Uihlein, A. |

| | | | | |
|---|---|---|---|---|
| 926 | Northern Europe | Single Family Residential | 2208.28 | Nemry, F., & Uihlein, A. |
| 927 | Northern Europe | Single Family Residential | 1868.19 | Nemry, F., & Uihlein, A. |
| 928 | Northern Europe | Single Family Residential | 2466.34 | Nemry, F., & Uihlein, A. |
| 929 | Northern Europe | Single Family Residential | 2471.32 | Nemry, F., & Uihlein, A. |
| 930 | Northern Europe | Single Family Residential | 1273.30 | Nemry, F., & Uihlein, A. |
| 931 | Northern Europe | Single Family Residential | 1280.89 | Nemry, F., & Uihlein, A. |
| 932 | Northern Europe | Multifamily Residential | 1928.50 | Nemry, F., & Uihlein, A. |
| 933 | Northern Europe | Multifamily Residential | 1294.48 | Nemry, F., & Uihlein, A. |
| 934 | Northern Europe | Multifamily Residential | 998.43 | Nemry, F., & Uihlein, A. |
| 935 | Northern Europe | Multifamily Residential | 1469.28 | Nemry, F., & Uihlein, A. |
| 936 | Northern Europe | Multifamily Residential | 1534.74 | Nemry, F., & Uihlein, A. |
| 937 | Northern Europe | Multifamily Residential | 299.81 | Nemry, F., & Uihlein, A. |
| 938 | Northern Europe | Multifamily Residential | 299.13 | Nemry, F., & Uihlein, A. |
| 939 | Northern Europe | Multifamily Residential | 1204.61 | Nemry, F., & Uihlein, A. |
| 940 | Northern Europe | Multifamily Residential | 1202.82 | Nemry, F., & Uihlein, A. |
| 941 | Northern Europe | Multifamily Residential | 1505.22 | Nemry, F., & Uihlein, A. |
| 942 | Northern Europe | Multifamily Residential | 1104.95 | Nemry, F., & Uihlein, A. |
| 943 | Northern Europe | Multifamily Residential | 985.48 | Nemry, F., & Uihlein, A. |
| 944 | Northern Europe | Multifamily Residential | 986.83 | Nemry, F., & Uihlein, A. |
| 945 | East Asia | Single Family Residential | 423.20 | Nishioka, Y., Yanagisawa, Y. & Spengler, J.D. |
| 946 | East Asia | Single Family Residential | 1289.20 | Nishioka, Y., Yanagisawa, Y. & Spengler, J.D. |
| 947 | Central Europe | Industrial | 133.49 | Ortlepp, Regine; Gruhler, Karin; Schiller, Georg |
| 948 | Central Europe | Multifamily Residential | 2128.47 | Sprecher, B, Verhagen, TJ, Sauer, ML, Baars, M, Heintz, J, & Fishman, T |

| | | | | |
|---|---|---|---|---|
| 949 | Central Europe | Single Family Residential | 356.90 | Sprecher, B, Verhagen, TJ, Sauer, ML, Baars, M, Heintz, J, & Fishman, T |
| 950 | Central Europe | Single Family Residential | 833.35 | Sprecher, B, Verhagen, TJ, Sauer, ML, Baars, M, Heintz, J, & Fishman, T |
| 951 | Central Europe | Single Family Residential | 737.65 | Sprecher, B, Verhagen, TJ, Sauer, ML, Baars, M, Heintz, J, & Fishman, T |
| 952 | Central Europe | Single Family Residential | 1941.15 | Sprecher, B, Verhagen, TJ, Sauer, ML, Baars, M, Heintz, J, & Fishman, T |
| 953 | Central Europe | Institutional | 1579.37 | Sprecher, B, Verhagen, TJ, Sauer, ML, Baars, M, Heintz, J, & Fishman, T |
| 954 | Central Europe | Commercial | 2270.02 | Sprecher, B, Verhagen, TJ, Sauer, ML, Baars, M, Heintz, J, & Fishman, T |
| 955 | Central Europe | Institutional | 2808.51 | Sprecher, B, Verhagen, TJ, Sauer, ML, Baars, M, Heintz, J, & Fishman, T |
| 956 | Central Europe | Single Family Residential | 371.02 | Sprecher, B, Verhagen, TJ, Sauer, ML, Baars, M, Heintz, J, & Fishman, T |
| 957 | Central Europe | Institutional | 2841.33 | Sprecher, B, Verhagen, TJ, Sauer, ML, Baars, M, Heintz, J, & Fishman, T |
| 958 | Central Europe | Institutional | 1908.67 | Sprecher, B, Verhagen, TJ, Sauer, ML, Baars, M, Heintz, J, & Fishman, T |
| 959 | Central Europe | Single Family Residential | 1001.76 | Sprecher, B, Verhagen, TJ, Sauer, ML, Baars, M, Heintz, J, & Fishman, T |
| 960 | Central Europe | Commercial | 441.16 | Sprecher, B, Verhagen, TJ, Sauer, ML, Baars, M, Heintz, J, & Fishman, T |
| 961 | Central Europe | Institutional | 1706.94 | Sprecher, B, Verhagen, TJ, Sauer, ML, Baars, M, Heintz, J, & Fishman, T |
| 962 | Central Europe | Commercial | 902.15 | Sprecher, B, Verhagen, TJ, Sauer, ML, Baars, M, Heintz, J, & Fishman, T |
| 963 | Central Europe | Institutional | 936.58 | Sprecher, B, Verhagen, TJ, Sauer, ML, Baars, M, Heintz, J, & Fishman, T |

| | | | | |
|---|---|---|---|---|
| 964 | Central Europe | Commercial | 1448.20 | Sprecher, B, Verhagen, TJ, Sauer, ML, Baars, M, Heintz, J, & Fishman, T |
| 965 | Central Europe | Institutional | 572.86 | Sprecher, B, Verhagen, TJ, Sauer, ML, Baars, M, Heintz, J, & Fishman, T |
| 966 | Central Europe | Commercial | 2620.33 | Sprecher, B, Verhagen, TJ, Sauer, ML, Baars, M, Heintz, J, & Fishman, T |
| 967 | Central Europe | Institutional | 2982.92 | Sprecher, B, Verhagen, TJ, Sauer, ML, Baars, M, Heintz, J, & Fishman, T |
| 968 | Central Europe | Commercial | 1892.95 | Sprecher, B, Verhagen, TJ, Sauer, ML, Baars, M, Heintz, J, & Fishman, T |
| 969 | Central Europe | Institutional | 1051.27 | Sprecher, B, Verhagen, TJ, Sauer, ML, Baars, M, Heintz, J, & Fishman, T |
| 970 | Central Europe | Institutional | 1522.18 | Sprecher, B, Verhagen, TJ, Sauer, ML, Baars, M, Heintz, J, & Fishman, T |
| 971 | Central Europe | Institutional | 805.37 | Sprecher, B, Verhagen, TJ, Sauer, ML, Baars, M, Heintz, J, & Fishman, T |
| 972 | Central Europe | Commercial | 1581.06 | Sprecher, B, Verhagen, TJ, Sauer, ML, Baars, M, Heintz, J, & Fishman, T |
| 973 | Central Europe | Institutional | 782.54 | Sprecher, B, Verhagen, TJ, Sauer, ML, Baars, M, Heintz, J, & Fishman, T |
| 974 | Central Europe | Misc | 1064.73 | Sprecher, B, Verhagen, TJ, Sauer, ML, Baars, M, Heintz, J, & Fishman, T |
| 975 | Central Europe | Misc | 2362.27 | Sprecher, B, Verhagen, TJ, Sauer, ML, Baars, M, Heintz, J, & Fishman, T |
| 976 | Central Europe | Institutional | 1050.61 | Sprecher, B, Verhagen, TJ, Sauer, ML, Baars, M, Heintz, J, & Fishman, T |
| 977 | Central Europe | Commercial | 1241.51 | Sprecher, B, Verhagen, TJ, Sauer, ML, Baars, M, Heintz, J, & Fishman, T |
| 978 | Central Europe | Commercial | 1575.37 | Sprecher, B, Verhagen, TJ, Sauer, ML, Baars, M, Heintz, J, & Fishman, T |

| | | | | |
|---|---|---|---|---|
| 979 | Central Europe | Commercial | 1381.11 | Sprecher, B, Verhagen, TJ, Sauer, ML, Baars, M, Heintz, J, & Fishman, T |
| 980 | Central Europe | Commercial | 1208.20 | Sprecher, B, Verhagen, TJ, Sauer, ML, Baars, M, Heintz, J, & Fishman, T |
| 981 | Central Europe | Institutional | 1555.52 | Sprecher, B, Verhagen, TJ, Sauer, ML, Baars, M, Heintz, J, & Fishman, T |
| 982 | Central Europe | Multifamily Residential | 830.63 | Sprecher, B, Verhagen, TJ, Sauer, ML, Baars, M, Heintz, J, & Fishman, T |
| 983 | Central Europe | Commercial | 1212.17 | Sprecher, B, Verhagen, TJ, Sauer, ML, Baars, M, Heintz, J, & Fishman, T |
| 984 | Central Europe | Commercial | 1217.75 | Sprecher, B, Verhagen, TJ, Sauer, ML, Baars, M, Heintz, J, & Fishman, T |
| 985 | Central Europe | Commercial | 906.25 | Sprecher, B, Verhagen, TJ, Sauer, ML, Baars, M, Heintz, J, & Fishman, T |
| 986 | Central Europe | Commercial | 1133.65 | Sprecher, B, Verhagen, TJ, Sauer, ML, Baars, M, Heintz, J, & Fishman, T |
| 987 | Central Europe | Commercial | 1384.80 | Sprecher, B, Verhagen, TJ, Sauer, ML, Baars, M, Heintz, J, & Fishman, T |
| 988 | Central Europe | Commercial | 538.15 | Sprecher, B, Verhagen, TJ, Sauer, ML, Baars, M, Heintz, J, & Fishman, T |
| 989 | Central Europe | Commercial | 927.29 | Sprecher, B, Verhagen, TJ, Sauer, ML, Baars, M, Heintz, J, & Fishman, T |
| 990 | Central Europe | Commercial | 1979.07 | Sprecher, B, Verhagen, TJ, Sauer, ML, Baars, M, Heintz, J, & Fishman, T |
| 991 | Central Europe | Commercial | 1528.21 | Sprecher, B, Verhagen, TJ, Sauer, ML, Baars, M, Heintz, J, & Fishman, T |
| 992 | Central Europe | Commercial | 507.43 | Sprecher, B, Verhagen, TJ, Sauer, ML, Baars, M, Heintz, J, & Fishman, T |
| 993 | Central Europe | Commercial | 1368.31 | Sprecher, B, Verhagen, TJ, Sauer, ML, Baars, M, Heintz, J, & Fishman, T |

| | | | | |
|---|---|---|---|---|
| 994 | Central Europe | Industrial | 836.53 | Sprecher, B, Verhagen, TJ, Sauer, ML, Baars, M, Heintz, J, & Fishman, T |
| 995 | Central Europe | Multifamily Residential | 1067.17 | Sprecher, B, Verhagen, TJ, Sauer, ML, Baars, M, Heintz, J, & Fishman, T |
| 996 | Central Europe | Multifamily Residential | 2123.32 | Sprecher, B, Verhagen, TJ, Sauer, ML, Baars, M, Heintz, J, & Fishman, T |
| 997 | Central Europe | Institutional | 1403.61 | Sprecher, B, Verhagen, TJ, Sauer, ML, Baars, M, Heintz, J, & Fishman, T |
| 998 | Central Europe | Institutional | 1118.66 | Sprecher, B, Verhagen, TJ, Sauer, ML, Baars, M, Heintz, J, & Fishman, T |
| 999 | Central Europe | Industrial | 1025.51 | Sprecher, B, Verhagen, TJ, Sauer, ML, Baars, M, Heintz, J, & Fishman, T |
| 1000 | Central Europe | Misc | 1066.35 | Sprecher, B, Verhagen, TJ, Sauer, ML, Baars, M, Heintz, J, & Fishman, T |
| 1001 | Central Europe | Multifamily Residential | 1030.22 | Sprecher, B, Verhagen, TJ, Sauer, ML, Baars, M, Heintz, J, & Fishman, T |
| 1002 | Central Europe | Commercial | 1322.62 | Sprecher, B, Verhagen, TJ, Sauer, ML, Baars, M, Heintz, J, & Fishman, T |
| 1003 | Central Europe | Commercial | 1789.12 | Sprecher, B, Verhagen, TJ, Sauer, ML, Baars, M, Heintz, J, & Fishman, T |
| 1004 | Central Europe | Commercial | 1553.22 | Sprecher, B, Verhagen, TJ, Sauer, ML, Baars, M, Heintz, J, & Fishman, T |
| 1005 | Central Europe | Commercial | 1309.40 | Sprecher, B, Verhagen, TJ, Sauer, ML, Baars, M, Heintz, J, & Fishman, T |
| 1006 | Central Europe | Commercial | 1039.71 | Sprecher, B, Verhagen, TJ, Sauer, ML, Baars, M, Heintz, J, & Fishman, T |
| 1007 | Central Europe | Multifamily Residential | 998.35 | Sprecher, B, Verhagen, TJ, Sauer, ML, Baars, M, Heintz, J, & Fishman, T |
| 1008 | Central Europe | Commercial | 959.38 | Sprecher, B, Verhagen, TJ, Sauer, ML, Baars, M, Heintz, J, & Fishman, T |
| 1009 | Oceania | Single Family Residential | 2583.50 | Surahman, Usep; Kubota, Tetsu; Higashi, Osamu |

| | | | | |
|---|---|---|---|---|
| 1010 | Oceania | Single Family Residential | 2562.60 | Surahman, Usep; Kubota, Tetsu; Higashi, Osamu |
| 1011 | Oceania | Single Family Residential | 2516.70 | Surahman, Usep; Kubota, Tetsu; Higashi, Osamu |
| 1012 | Oceania | Single Family Residential | 2169.90 | Surahman, Usep; Kubota, Tetsu; Higashi, Osamu |
| 1013 | Oceania | Single Family Residential | 2765.10 | Surahman, Usep; Kubota, Tetsu; Higashi, Osamu |
| 1014 | Oceania | Single Family Residential | 2861.20 | Surahman, Usep; Kubota, Tetsu; Higashi, Osamu |
| 1015 | East Asia | Single Family Residential | 469.70 | Surahman, Usep; Kubota, Tetsu; Higashi, Osamu |
| 1016 | Southern Europe | Single Family Residential | 1961.53 | Surahman, Usep; Kubota, Tetsu; Higashi, Osamu |
| 1017 | Southern Europe | Multifamily Residential | 2546.00 | Syngros G, Balaras CA, Koubogiannis DG |
| 1018 | Southern Europe | Multifamily Residential | 2253.00 | Syngros G, Balaras CA, Koubogiannis DG |
| 1019 | Southern Europe | Single Family Residential | 3491.00 | Syngros G, Balaras CA, Koubogiannis DG |
| 1020 | Southern Europe | Single Family Residential | 3221.00 | Syngros G, Balaras CA, Koubogiannis DG |
| 1021 | East Asia | Misc | 1461.00 | Wang H, Chen D, Duan H, Yin F, Niu Y. |
| 1022 | East Asia | Misc | 1490.00 | Wang H, Chen D, Duan H, Yin F, Niu Y. |
| 1023 | East Asia | Single Family Residential | 415.54 | Zhang X, Zhang X. |
| 1024 | East Asia | Single Family Residential | 1705.04 | Zhang X, Zhang X. |
| 1025 | East Asia | Single Family Residential | 827.69 | Zhang X, Zhang X. |
| 1026 | East Asia | Misc | 1461.00 | Wang H, Chen D, Duan H, Yin F, Niu Y. |
| 1027 | East Asia | Misc | 1490.00 | Wang H, Chen D, Duan H, Yin F, Niu Y. |
| 1028 | East Asia | Single Family Residential | 827.69 | Zhang X, Zhang X. |

# 2. Elemental Compositions of Building Materials

This subsection provides the data and assumptions used to calculate the elemental compositions of building materials. **Table S-2** provides the elemental composition (categorized by scientific elements) for the building materials. **Tables S-3** and **S-4** provide the assumptions and source information used to quantify the elemental composition for each material.

**Table S-2.** The elemental compositions (by percentage) of building materials.

| Material | Material Sheet | Element (%) | | | | | | | | | | |
|---|---|---|---|---|---|---|---|---|---|---|---|---|
| Crushed Coarse Aggregate | Aggregates | **H** | **B** | **C** | **N** | **O** | **F** | **Na** | **Mg** | **Al** | **Si** | **P** |
| | | 0 | 0 | 0.113 | 0 | 0.480 | 0 | 0 | 0.002 | 0.003 | 0.019 | 0 |
| | | **S** | **Cl** | **K** | **Ca** | **Ti** | **V** | **Cr** | **Mn** | **Fe** | **Co** | **Ni** |
| | | 0.001 | 0 | 0.001 | 0.378 | 0 | 0 | 0 | 0 | 0.002 | 0 | 0 |
| | | **Cu** | **Zn** | **Br** | **Sr** | **Zr** | **Nb** | **Mo** | **Sn** | **Ba** | **Pb** | **Bi** |
| | | 0 | 0 | 0 | 0 | 0 | 0 | 0 | 0 | 0 | 0 | 0 |
| Natural Coarse Aggregate | Aggregates | **H** | **B** | **C** | **N** | **O** | **F** | **Na** | **Mg** | **Al** | **Si** | **P** |
| | | 0 | 0 | 0.029 | 0 | 0.484 | 0 | 0.012 | 0.014 | 0.044 | 0.256 | 0 |
| | | **S** | **Cl** | **K** | **Ca** | **Ti** | **V** | **Cr** | **Mn** | **Fe** | **Co** | **Ni** |
| | | 0 | 0 | 0.009 | 0.123 | 0.002 | 0 | 0 | 0.001 | 0.025 | 0 | 0 |
| | | **Cu** | **Zn** | **Br** | **Sr** | **Zr** | **Nb** | **Mo** | **Sn** | **Ba** | **Pb** | **Bi** |
| | | 0 | 0 | 0 | 0 | 0 | 0 | 0 | 0 | 0 | 0 | 0 |
| Crushed Fine Aggregate | Aggregates | **H** | **B** | **C** | **N** | **O** | **F** | **Na** | **Mg** | **Al** | **Si** | **P** |
| | | 0 | 0 | 0.113 | 0 | 0.480 | 0 | 0 | 0.002 | 0.003 | 0.019 | 0 |
| | | **S** | **Cl** | **K** | **Ca** | **Ti** | **V** | **Cr** | **Mn** | **Fe** | **Co** | **Ni** |
| | | 0.001 | 0 | 0.001 | 0.378 | 0 | 0 | 0 | 0 | 0.002 | 0 | 0 |
| | | **Cu** | **Zn** | **Br** | **Sr** | **Zr** | **Nb** | **Mo** | **Sn** | **Ba** | **Pb** | **Bi** |
| | | 0 | 0 | 0 | 0 | 0 | 0 | 0 | 0 | 0 | 0 | 0 |

| | | | | | | | | | | | | |
|---|---|---|---|---|---|---|---|---|---|---|---|---|
| Recycled Concrete Aggregate | Aggregates | **H** | **B** | **C** | **N** | **O** | **F** | **Na** | **Mg** | **Al** | **Si** | **P** |
| | | 0 | 0 | 0 | 0 | 0.411 | 0 | 0.043 | 0.021 | 0.059 | 0.204 | 0 |
| | | **S** | **Cl** | **K** | **Ca** | **Ti** | **V** | **Cr** | **Mn** | **Fe** | **Co** | **Ni** |
| | | 0.006 | 0 | 0.012 | 0.214 | 0.002 | 0 | 0 | 0 | 0.029 | 0 | 0 |
| | | **Cu** | **Zn** | **Br** | **Sr** | **Zr** | **Nb** | **Mo** | **Sn** | **Ba** | **Pb** | **Bi** |
| | | 0 | 0 | 0 | 0 | 0 | 0 | 0 | 0 | 0 | 0 | 0 |
| Natural Fine Aggregate | Aggregates | **H** | **B** | **C** | **N** | **O** | **F** | **Na** | **Mg** | **Al** | **Si** | **P** |
| | | 0 | 0 | 0.015 | 0 | 0.496 | 0 | 0.016 | 0.015 | 0.057 | 0.314 | 0 |
| | | **S** | **Cl** | **K** | **Ca** | **Ti** | **V** | **Cr** | **Mn** | **Fe** | **Co** | **Ni** |
| | | 0 | 0 | 0.019 | 0.045 | 0 | 0 | 0 | 0 | 0.023 | 0 | 0 |
| | | **Cu** | **Zn** | **Br** | **Sr** | **Zr** | **Nb** | **Mo** | **Sn** | **Ba** | **Pb** | **Bi** |
| | | 0 | 0 | 0 | 0 | 0 | 0 | 0 | 0 | 0 | 0 | 0 |
| Coarse Aggregate Average | Aggregates | **H** | **B** | **C** | **N** | **O** | **F** | **Na** | **Mg** | **Al** | **Si** | **P** |
| | | 0 | 0 | 0.047 | 0 | 0.458 | 0 | 0.018 | 0.012 | 0.035 | 0.159 | 0 |
| | | **S** | **Cl** | **K** | **Ca** | **Ti** | **V** | **Cr** | **Mn** | **Fe** | **Co** | **Ni** |
| | | 0.002 | 0 | 0.007 | 0.238 | 0.001 | 0 | 0 | 0.001 | 0.019 | 0 | 0 |
| | | **Cu** | **Zn** | **Br** | **Sr** | **Zr** | **Nb** | **Mo** | **Sn** | **Ba** | **Pb** | **Bi** |
| | | 0 | 0 | 0 | 0 | 0 | 0 | 0 | 0 | 0 | 0 | 0 |
| Fine Aggregate Average | Aggregates | **H** | **B** | **C** | **N** | **O** | **F** | **Na** | **Mg** | **Al** | **Si** | **P** |
| | | 0 | 0 | 0.064 | 0 | 0.488 | 0 | 0.008 | 0.008 | 0.030 | 0.167 | 0 |
| | | **S** | **Cl** | **K** | **Ca** | **Ti** | **V** | **Cr** | **Mn** | **Fe** | **Co** | **Ni** |
| | | 0.001 | 0 | 0.010 | 0.211 | 0 | 0 | 0 | 0 | 0.012 | 0 | 0 |
| | | **Cu** | **Zn** | **Br** | **Sr** | **Zr** | **Nb** | **Mo** | **Sn** | **Ba** | **Pb** | **Bi** |
| | | 0 | 0 | 0 | 0 | 0 | 0 | 0 | 0 | 0 | 0 | 0 |
| | | **H** | **B** | **C** | **N** | **O** | **F** | **Na** | **Mg** | **Al** | **Si** | **P** |

| | | | | | | | | | | | | |
|---|---|---|---|---|---|---|---|---|---|---|---|---|
| Aluminum Window Frame | Aluminum Window Frame | 0 | 0 | 0 | 0 | 0 | 0 | 0 | 0.009 | 0.976 | 0.006 | 0 |
| | | **S** | **Cl** | **K** | **Ca** | **Ti** | **V** | **Cr** | **Mn** | **Fe** | **Co** | **Ni** |
| | | 0 | 0 | 0 | 0 | 0.001 | 0 | 0.001 | 0.001 | 0.004 | 0 | 0 |
| | | **Cu** | **Zn** | **Br** | **Sr** | **Zr** | **Nb** | **Mo** | **Sn** | **Ba** | **Pb** | **Bi** |
| | | 0.001 | 0.001 | 0 | 0 | 0 | 0 | 0 | 0 | 0 | 0 | 0 |
| Asphalt Shingles | Asphalt Shingles | **H** | **B** | **C** | **N** | **O** | **F** | **Na** | **Mg** | **Al** | **Si** | **P** |
| | | 0.018 | 0 | 0.398 | 0.002 | 0.205 | 0 | 0.007 | 0.007 | 0.021 | 0.126 | 0 |
| | | **S** | **Cl** | **K** | **Ca** | **Ti** | **V** | **Cr** | **Mn** | **Fe** | **Co** | **Ni** |
| | | 0.008 | 0 | 0.005 | 0.188 | 0.001 | 0 | 0 | 0.001 | 0.012 | 0 | 0 |
| | | **Cu** | **Zn** | **Br** | **Sr** | **Zr** | **Nb** | **Mo** | **Sn** | **Ba** | **Pb** | **Bi** |
| | | 0 | 0 | 0 | 0 | 0 | 0 | 0 | 0 | 0 | 0 | 0 |
| Brick | Brick | **H** | **B** | **C** | **N** | **O** | **F** | **Na** | **Mg** | **Al** | **Si** | **P** |
| | | 0 | 0 | 0 | 0 | 0.515 | 0 | 0.001 | 0.008 | 0.110 | 0.243 | 0 |
| | | **S** | **Cl** | **K** | **Ca** | **Ti** | **V** | **Cr** | **Mn** | **Fe** | **Co** | **Ni** |
| | | 0 | 0 | 0.024 | 0.002 | 0.006 | 0 | 0 | 0 | 0.091 | 0 | 0 |
| | | **Cu** | **Zn** | **Br** | **Sr** | **Zr** | **Nb** | **Mo** | **Sn** | **Ba** | **Pb** | **Bi** |
| | | 0 | 0 | 0 | 0 | 0 | 0 | 0 | 0 | 0 | 0 | 0 |
| Carpet | Carpet | **H** | **B** | **C** | **N** | **O** | **F** | **Na** | **Mg** | **Al** | **Si** | **P** |
| | | 0.064 | 0 | 0.473 | 0.044 | 0.232 | 0 | 0.001 | 0 | 0.022 | 0.004 | 0 |
| | | **S** | **Cl** | **K** | **Ca** | **Ti** | **V** | **Cr** | **Mn** | **Fe** | **Co** | **Ni** |
| | | 0.004 | 0.083 | 0 | 0.072 | 0 | 0 | 0 | 0 | 0 | 0 | 0 |
| | | **Cu** | **Zn** | **Br** | **Sr** | **Zr** | **Nb** | **Mo** | **Sn** | **Ba** | **Pb** | **Bi** |
| | | 0 | 0 | 0 | 0 | 0 | 0 | 0 | 0 | 0 | 0 | 0 |
| Cellulose Insulation | Cellulose Insulation | **H** | **B** | **C** | **N** | **O** | **F** | **Na** | **Mg** | **Al** | **Si** | **P** |
| | | 0.059 | 0.009 | 0.390 | 0.009 | 0.467 | 0 | 0 | 0 | 0.002 | 0.005 | 0 |

| | | | | | | | | | | | | |
|---|---|---|---|---|---|---|---|---|---|---|---|---|
| | | **S** | **Cl** | **K** | **Ca** | **Ti** | **V** | **Cr** | **Mn** | **Fe** | **Co** | **Ni** |
| | | 0.011 | 0 | 0.001 | 0.046 | 0 | 0 | 0 | 0 | 0.002 | 0 | 0 |
| | | **Cu** | **Zn** | **Br** | **Sr** | **Zr** | **Nb** | **Mo** | **Sn** | **Ba** | **Pb** | **Bi** |
| | | 0 | 0 | 0 | 0 | 0 | 0 | 0 | 0 | 0 | 0 | 0 |
| Cement | Cementitious Materials | **H** | **B** | **C** | **N** | **O** | **F** | **Na** | **Mg** | **Al** | **Si** | **P** |
| | | 0 | 0 | 0.003 | 0 | 0.363 | 0.001 | 0.002 | 0.024 | 0.025 | 0.095 | 0 |
| | | **S** | **Cl** | **K** | **Ca** | **Ti** | **V** | **Cr** | **Mn** | **Fe** | **Co** | **Ni** |
| | | 0.011 | 0 | 0.004 | 0.446 | 0.001 | 0 | 0 | 0.001 | 0.021 | 0 | 0 |
| | | **Cu** | **Zn** | **Br** | **Sr** | **Zr** | **Nb** | **Mo** | **Sn** | **Ba** | **Pb** | **Bi** |
| | | 0 | 0 | 0 | 0.001 | 0 | 0 | 0 | 0 | 0 | 0 | 0 |
| Mortar | Cementitious Materials | **H** | **B** | **C** | **N** | **O** | **F** | **Na** | **Mg** | **Al** | **Si** | **P** |
| | | 0.012 | 0 | 0.011 | 0 | 0.509 | 0 | 0.012 | 0.015 | 0.044 | 0.234 | 0 |
| | | **S** | **Cl** | **K** | **Ca** | **Ti** | **V** | **Cr** | **Mn** | **Fe** | **Co** | **Ni** |
| | | 0.003 | 0 | 0.014 | 0.126 | 0.001 | 0 | 0 | 0 | 0.020 | 0 | 0 |
| | | **Cu** | **Zn** | **Br** | **Sr** | **Zr** | **Nb** | **Mo** | **Sn** | **Ba** | **Pb** | **Bi** |
| | | 0 | 0 | 0 | 0 | 0 | 0 | 0 | 0 | 0 | 0 | 0 |
| Concrete | Concrete (5,000 psi, 40% SCM) Cementitious Materials | **H** | **B** | **C** | **N** | **O** | **F** | **Na** | **Mg** | **Al** | **Si** | **P** |
| | | 0.008 | 0 | 0.077 | 0 | 0.466 | 0 | 0.006 | 0.011 | 0.036 | 0.154 | 0.001 |
| | | **S** | **Cl** | **K** | **Ca** | **Ti** | **V** | **Cr** | **Mn** | **Fe** | **Co** | **Ni** |
| | | 0.002 | 0 | 0.008 | 0.214 | 0.001 | 0 | 0 | 0 | 0.016 | 0 | 0 |
| | | **Cu** | **Zn** | **Br** | **Sr** | **Zr** | **Nb** | **Mo** | **Sn** | **Ba** | **Pb** | **Bi** |
| | | 0 | 0 | 0 | 0 | 0 | 0 | 0 | 0 | 0 | 0 | 0 |
| Ceramic Tiles | Ceramic Tiles | **H** | **B** | **C** | **N** | **O** | **F** | **Na** | **Mg** | **Al** | **Si** | **P** |
| | | 0.012 | 0 | 0.011 | 0.001 | 0.531 | 0 | 0.005 | 0.004 | 0.178 | 0.229 | 0 |
| | | **S** | **Cl** | **K** | **Ca** | **Ti** | **V** | **Cr** | **Mn** | **Fe** | **Co** | **Ni** |

| | | | | | | | | | | | | |
|---|---|---|---|---|---|---|---|---|---|---|---|---|
| | | 0.001 | 0 | 0.008 | 0.017 | 0 | 0 | 0 | 0 | 0.002 | 0.001 | 0 |
| | | **Cu** | **Zn** | **Br** | **Sr** | **Zr** | **Nb** | **Mo** | **Sn** | **Ba** | **Pb** | **Bi** |
| | | 0 | 0 | 0 | 0 | 0 | 0 | 0 | 0 | 0 | 0 | 0 |
| Cross Laminated Timber | Cross Laminated Timber | **H** | **B** | **C** | **N** | **O** | **F** | **Na** | **Mg** | **Al** | **Si** | **P** |
| | | 0.062 | 0 | 0.498 | 0.001 | 0.439 | 0 | 0 | 0 | 0 | 0 | 0 |
| | | **S** | **Cl** | **K** | **Ca** | **Ti** | **V** | **Cr** | **Mn** | **Fe** | **Co** | **Ni** |
| | | 0 | 0 | 0 | 0 | 0 | 0 | 0 | 0 | 0 | 0 | 0 |
| | | **Cu** | **Zn** | **Br** | **Sr** | **Zr** | **Nb** | **Mo** | **Sn** | **Ba** | **Pb** | **Bi** |
| | | 0 | 0 | 0 | 0 | 0 | 0 | 0 | 0 | 0 | 0 | 0 |
| Dimensional Lumber and Window Frames | Dimensional Lumber and Window Frames | **H** | **B** | **C** | **N** | **O** | **F** | **Na** | **Mg** | **Al** | **Si** | **P** |
| | | 0.062 | 0 | 0.498 | 0 | 0.440 | 0 | 0 | 0 | 0 | 0 | 0 |
| | | **S** | **Cl** | **K** | **Ca** | **Ti** | **V** | **Cr** | **Mn** | **Fe** | **Co** | **Ni** |
| | | 0 | 0 | 0 | 0 | 0 | 0 | 0 | 0 | 0 | 0 | 0 |
| | | **Cu** | **Zn** | **Br** | **Sr** | **Zr** | **Nb** | **Mo** | **Sn** | **Ba** | **Pb** | **Bi** |
| | | 0 | 0 | 0 | 0 | 0 | 0 | 0 | 0 | 0 | 0 | 0 |
| EPDM | EPDM | **H** | **B** | **C** | **N** | **O** | **F** | **Na** | **Mg** | **Al** | **Si** | **P** |
| | | 0.140 | 0 | 0.860 | 0 | 0 | 0 | 0 | 0 | 0 | 0 | 0 |
| | | **S** | **Cl** | **K** | **Ca** | **Ti** | **V** | **Cr** | **Mn** | **Fe** | **Co** | **Ni** |
| | | 0 | 0 | 0 | 0 | 0 | 0 | 0 | 0 | 0 | 0 | 0 |
| | | **Cu** | **Zn** | **Br** | **Sr** | **Zr** | **Nb** | **Mo** | **Sn** | **Ba** | **Pb** | **Bi** |
| | | 0 | 0 | 0 | 0 | 0 | 0 | 0 | 0 | 0 | 0 | 0 |
| Fiber Cement | Fiber Cement | **H** | **B** | **C** | **N** | **O** | **F** | **Na** | **Mg** | **Al** | **Si** | **P** |
| | | 0.017 | 0 | 0.201 | 0 | 0.295 | 0 | 0.002 | 0.012 | 0.025 | 0.127 | 0 |
| | | **S** | **Cl** | **K** | **Ca** | **Ti** | **V** | **Cr** | **Mn** | **Fe** | **Co** | **Ni** |
| | | 0.006 | 0 | 0.014 | 0.275 | 0.004 | 0 | 0.004 | 0 | 0.016 | 0 | 0 |

| | | | | | | | | | | | | |
|---|---|---|---|---|---|---|---|---|---|---|---|---|
| | | **Cu** | **Zn** | **Br** | **Sr** | **Zr** | **Nb** | **Mo** | **Sn** | **Ba** | **Pb** | **Bi** |
| | | 0 | 0 | 0 | 0 | 0 | 0 | 0 | 0 | 0 | 0 | 0 |
| Fiberglass Batt | Fiberglass Batt | **H** | **B** | **C** | **N** | **O** | **F** | **Na** | **Mg** | **Al** | **Si** | **P** |
| | | 0.011 | 0.014 | 0.036 | 0.011 | 0.494 | 0 | 0.093 | 0.008 | 0.021 | 0.222 | 0 |
| | | **S** | **Cl** | **K** | **Ca** | **Ti** | **V** | **Cr** | **Mn** | **Fe** | **Co** | **Ni** |
| | | 0.001 | 0 | 0.007 | 0.073 | 0 | 0 | 0 | 0 | 0.008 | 0 | 0 |
| | | **Cu** | **Zn** | **Br** | **Sr** | **Zr** | **Nb** | **Mo** | **Sn** | **Ba** | **Pb** | **Bi** |
| | | 0 | 0 | 0 | 0 | 0 | 0 | 0 | 0 | 0 | 0 | 0 |
| Fiberglass Loose Fill | Fiberglass Loose Fill | **H** | **B** | **C** | **N** | **O** | **F** | **Na** | **Mg** | **Al** | **Si** | **P** |
| | | 0.008 | 0.015 | 0.028 | 0.002 | 0.505 | 0 | 0.095 | 0.008 | 0.022 | 0.226 | 0 |
| | | **S** | **Cl** | **K** | **Ca** | **Ti** | **V** | **Cr** | **Mn** | **Fe** | **Co** | **Ni** |
| | | 0 | 0 | 0.007 | 0.075 | 0 | 0 | 0 | 0 | 0.008 | 0 | 0 |
| | | **Cu** | **Zn** | **Br** | **Sr** | **Zr** | **Nb** | **Mo** | **Sn** | **Ba** | **Pb** | **Bi** |
| | | 0 | 0 | 0 | 0 | 0 | 0 | 0 | 0 | 0 | 0 | 0 |
| Fiberglass Window Frame | Fiberglass Window Frame | **H** | **B** | **C** | **N** | **O** | **F** | **Na** | **Mg** | **Al** | **Si** | **P** |
| | | 0.020 | 0.011 | 0.216 | 0 | 0.401 | 0.004 | 0.003 | 0.048 | 0.052 | 0.183 | 0 |
| | | **S** | **Cl** | **K** | **Ca** | **Ti** | **V** | **Cr** | **Mn** | **Fe** | **Co** | **Ni** |
| | | 0 | 0 | 0.003 | 0.057 | 0.003 | 0 | 0 | 0 | 0.001 | 0 | 0 |
| | | **Cu** | **Zn** | **Br** | **Sr** | **Zr** | **Nb** | **Mo** | **Sn** | **Ba** | **Pb** | **Bi** |
| | | 0 | 0 | 0 | 0 | 0 | 0 | 0 | 0 | 0 | 0 | 0 |
| Fire-rated Type X Gypsum Board | Fire-rated Type X Gypsum Board | **H** | **B** | **C** | **N** | **O** | **F** | **Na** | **Mg** | **Al** | **Si** | **P** |
| | | 0.059 | 0 | 0.012 | 0 | 0.684 | 0 | 0 | 0 | 0 | 0 | 0 |
| | | **S** | **Cl** | **K** | **Ca** | **Ti** | **V** | **Cr** | **Mn** | **Fe** | **Co** | **Ni** |
| | | 0.109 | 0 | 0 | 0.136 | 0 | 0 | 0 | 0 | 0 | 0 | 0 |
| | | **Cu** | **Zn** | **Br** | **Sr** | **Zr** | **Nb** | **Mo** | **Sn** | **Ba** | **Pb** | **Bi** |

| | | | | | | | | | | | | |
|---|---|---|---|---|---|---|---|---|---|---|---|---|
| | | 0 | 0 | 0 | 0 | 0 | 0 | 0 | 0 | 0 | 0 | 0 |
| Polyiso Foam Insulation | Foam Insulation | **H** | **B** | **C** | **N** | **O** | **F** | **Na** | **Mg** | **Al** | **Si** | **P** |
| | | 0.058 | 0 | 0.662 | 0.063 | 0.199 | 0 | 0 | 0 | 0 | 0 | 0.003 |
| | | **S** | **Cl** | **K** | **Ca** | **Ti** | **V** | **Cr** | **Mn** | **Fe** | **Co** | **Ni** |
| | | 0 | 0.011 | 0.003 | 0 | 0 | 0 | 0 | 0 | 0 | 0 | 0 |
| | | **Cu** | **Zn** | **Br** | **Sr** | **Zr** | **Nb** | **Mo** | **Sn** | **Ba** | **Pb** | **Bi** |
| | | | | | | | | | | | | |
| Polyurethane Foam Insulation | Foam Insulation | **H** | **B** | **C** | **N** | **O** | **F** | **Na** | **Mg** | **Al** | **Si** | **P** |
| | | 0.056 | 0 | 0.558 | 0.057 | 0.237 | 0.036 | 0 | 0 | 0 | 0.003 | 0.012 |
| | | **S** | **Cl** | **K** | **Ca** | **Ti** | **V** | **Cr** | **Mn** | **Fe** | **Co** | **Ni** |
| | | 0 | 0.040 | 0 | 0 | 0 | 0 | 0 | 0 | 0 | 0 | 0 |
| | | **Cu** | **Zn** | **Br** | **Sr** | **Zr** | **Nb** | **Mo** | **Sn** | **Ba** | **Pb** | **Bi** |
| | | 0 | 0 | 0 | 0 | 0 | 0 | 0 | 0 | 0 | 0 | 0 |
| Glass Mat Gypsum Panel (5-8”) | Glass Mat Gypsum Panel | **H** | **B** | **C** | **N** | **O** | **F** | **Na** | **Mg** | **Al** | **Si** | **P** |
| | | 0.062 | 0 | 0.002 | 0 | 0.700 | 0 | 0.002 | 0 | 0.001 | 0.007 | 0 |
| | | **S** | **Cl** | **K** | **Ca** | **Ti** | **V** | **Cr** | **Mn** | **Fe** | **Co** | **Ni** |
| | | 0.099 | 0 | 0 | 0.126 | 0 | 0 | 0 | 0 | 0 | 0 | 0 |
| | | **Cu** | **Zn** | **Br** | **Sr** | **Zr** | **Nb** | **Mo** | **Sn** | **Ba** | **Pb** | **Bi** |
| | | 0 | 0 | 0 | 0 | 0 | 0 | 0 | 0 | 0 | 0 | 0 |
| Glued Laminated Timber | Glued Laminated Timber | **H** | **B** | **C** | **N** | **O** | **F** | **Na** | **Mg** | **Al** | **Si** | **P** |
| | | 0.062 | 0 | 0.500 | 0 | 0.438 | 0 | 0 | 0 | 0 | 0 | 0 |
| | | **S** | **Cl** | **K** | **Ca** | **Ti** | **V** | **Cr** | **Mn** | **Fe** | **Co** | **Ni** |
| | | 0 | 0 | 0 | 0 | 0 | 0 | 0 | 0 | 0 | 0 | 0 |
| | | **Cu** | **Zn** | **Br** | **Sr** | **Zr** | **Nb** | **Mo** | **Sn** | **Ba** | **Pb** | **Bi** |
| | | 0 | 0 | 0 | 0 | 0 | 0 | 0 | 0 | 0 | 0 | 0 |

| | | | | | | | | | | | | |
|---|---|---|---|---|---|---|---|---|---|---|---|---|
| Gypsum Board | Gypsum Board | **H** | **B** | **C** | **N** | **O** | **F** | **Na** | **Mg** | **Al** | **Si** | **P** |
| | | 0.024 | 0 | 0.002 | 0 | 0.557 | 0 | 0 | 0 | 0 | 0 | 0 |
| | | **S** | **Cl** | **K** | **Ca** | **Ti** | **V** | **Cr** | **Mn** | **Fe** | **Co** | **Ni** |
| | | 0.185 | 0 | 0 | 0.232 | 0 | 0 | 0 | 0 | 0 | 0 | 0 |
| | | **Cu** | **Zn** | **Br** | **Sr** | **Zr** | **Nb** | **Mo** | **Sn** | **Ba** | **Pb** | **Bi** |
| | | 0 | 0 | 0 | 0 | 0 | 0 | 0 | 0 | 0 | 0 | 0 |
| Heraklith | Heraklith | **H** | **B** | **C** | **N** | **O** | **F** | **Na** | **Mg** | **Al** | **Si** | **P** |
| | | 0.044 | 0 | 0.141 | 0 | 0.511 | 0 | 0.001 | 0.011 | 0.012 | 0.045 | 0 |
| | | **S** | **Cl** | **K** | **Ca** | **Ti** | **V** | **Cr** | **Mn** | **Fe** | **Co** | **Ni** |
| | | 0.005 | 0 | 0.002 | 0.214 | 0.001 | 0 | 0 | 0 | 0.010 | 0 | 0 |
| | | **Cu** | **Zn** | **Br** | **Sr** | **Zr** | **Nb** | **Mo** | **Sn** | **Ba** | **Pb** | **Bi** |
| | | 0 | 0 | 0 | 0 | 0 | 0 | 0 | 0 | 0 | 0 | 0 |
| Joint Compound | Joint Compound | **H** | **B** | **C** | **N** | **O** | **F** | **Na** | **Mg** | **Al** | **Si** | **P** |
| | | 0 | 0 | 0.085 | 0 | 0.473 | 0 | 0.004 | 0.016 | 0.026 | 0.073 | 0 |
| | | **S** | **Cl** | **K** | **Ca** | **Ti** | **V** | **Cr** | **Mn** | **Fe** | **Co** | **Ni** |
| | | 0.001 | 0 | 0.023 | 0.287 | 0 | 0 | 0 | 0 | 0.011 | 0 | 0 |
| | | **Cu** | **Zn** | **Br** | **Sr** | **Zr** | **Nb** | **Mo** | **Sn** | **Ba** | **Pb** | **Bi** |
| | | 0 | 0 | 0 | 0 | 0 | 0 | 0 | 0 | 0 | 0 | 0 |
| Latex Paint | Latex Paint | **H** | **B** | **C** | **N** | **O** | **F** | **Na** | **Mg** | **Al** | **Si** | **P** |
| | | 0.075 | 0 | 0.174 | 0 | 0.626 | 0 | 0 | 0 | 0 | 0.002 | |
| | | **S** | **Cl** | **K** | **Ca** | **Ti** | **V** | **Cr** | **Mn** | **Fe** | **Co** | **Ni** |
| | | 0 | 0 | 0 | 0.047 | 0.075 | 0 | 0 | 0 | 0 | 0 | 0 |
| | | **Cu** | **Zn** | **Br** | **Sr** | **Zr** | **Nb** | **Mo** | **Sn** | **Ba** | **Pb** | **Bi** |
| | | 0 | 0 | 0 | 0 | 0 | 0 | 0 | 0 | 0 | 0 | 0 |
| Linoleum | Linoleum | **H** | **B** | **C** | **N** | **O** | **F** | **Na** | **Mg** | **Al** | **Si** | **P** |

| | | | | | | | | | | | | |
|---|---|---|---|---|---|---|---|---|---|---|---|---|
| | | 0 | 0 | 0.353 | 0.003 | 0.286 | 0.002 | 0.001 | 0 | 0 | 0.002 | 0.001 |
| | | **S** | **Cl** | **K** | **Ca** | **Ti** | **V** | **Cr** | **Mn** | **Fe** | **Co** | **Ni** |
| | | 0.002 | 0.002 | 0 | 0.079 | 0.003 | 0 | 0.004 | 0 | 0.005 | 0 | 0 |
| | | **Cu** | **Zn** | **Br** | **Sr** | **Zr** | **Nb** | **Mo** | **Sn** | **Ba** | **Pb** | **Bi** |
| | | 0 | 0 | 0 | 0 | 0 | 0 | 0 | 0 | 0 | 0 | 0 |
| Metal Roof Cladding | Metal Roof Cladding | **H** | **B** | **C** | **N** | **O** | **F** | **Na** | **Mg** | **Al** | **Si** | **P** |
| | | 0 | 0 | 0.001 | 0 | 0 | 0 | 0 | 0 | 0 | 0 | 0 |
| | | **S** | **Cl** | **K** | **Ca** | **Ti** | **V** | **Cr** | **Mn** | **Fe** | **Co** | **Ni** |
| | | 0 | 0 | 0 | 0 | 0 | 0 | 0.001 | 0.006 | 0.985 | 0 | 0.002 |
| | | **Cu** | **Zn** | **Br** | **Sr** | **Zr** | **Nb** | **Mo** | **Sn** | **Ba** | **Pb** | **Bi** |
| | | 0.002 | 0 | 0 | 0 | 0 | 0 | 0.001 | 0 | 0 | 0 | 0 |
| Mineral Fill | Mineral Fill | **H** | **B** | **C** | **N** | **O** | **F** | **Na** | **Mg** | **Al** | **Si** | **P** |
| | | 0.001 | 0 | 0.010 | 0 | 0.426 | 0 | 0.004 | 0.006 | 0.090 | 0.178 | 0 |
| | | **S** | **Cl** | **K** | **Ca** | **Ti** | **V** | **Cr** | **Mn** | **Fe** | **Co** | **Ni** |
| | | 0.010 | 0 | 0.005 | 0.256 | 0.001 | 0 | 0 | 0.001 | 0.009 | 0 | 0 |
| | | **Cu** | **Zn** | **Br** | **Sr** | **Zr** | **Nb** | **Mo** | **Sn** | **Ba** | **Pb** | **Bi** |
| | | 0 | 0 | 0 | 0 | 0 | 0 | 0 | 0 | 0 | 0 | 0 |
| Mineral Wool (Rockwool) | Mineral Wool | **H** | **B** | **C** | **N** | **O** | **F** | **Na** | **Mg** | **Al** | **Si** | **P** |
| | | 0.002 | 0 | 0.018 | 0.007 | 0.417 | 0 | 0.008 | 0.011 | 0.078 | 0.186 | 0 |
| | | **S** | **Cl** | **K** | **Ca** | **Ti** | **V** | **Cr** | **Mn** | **Fe** | **Co** | **Ni** |
| | | 0.009 | 0 | 0.007 | 0.229 | 0.002 | 0 | 0 | 0.001 | 0.026 | 0 | 0 |
| | | **Cu** | **Zn** | **Br** | **Sr** | **Zr** | **Nb** | **Mo** | **Sn** | **Ba** | **Pb** | **Bi** |
| | | 0 | 0 | 0 | 0 | 0 | 0 | 0 | 0 | 0 | 0 | 0 |
| Modified Bitumen Membrane | Modified Bitumen Membrane | **H** | **B** | **C** | **N** | **O** | **F** | **Na** | **Mg** | **Al** | **Si** | **P** |
| | | 0.056 | 0.004 | 0.488 | 0.005 | 0.232 | 0 | 0.003 | 0.003 | 0.010 | 0.058 | 0 |

| | | | | | | | | | | | | |
|---|---|---|---|---|---|---|---|---|---|---|---|---|
| | | **S** | **Cl** | **K** | **Ca** | **Ti** | **V** | **Cr** | **Mn** | **Fe** | **Co** | **Ni** |
| | | 0.018 | 0 | 0.002 | 0.114 | 0 | 0 | 0 | 0 | 0.005 | 0 | 0 |
| | | **Cu** | **Zn** | **Br** | **Sr** | **Zr** | **Nb** | **Mo** | **Sn** | **Ba** | **Pb** | **Bi** |
| | | 0 | 0 | 0 | 0 | 0 | 0 | 0 | 0 | 0 | 0 | 0 |
| Oil Paint Stain | Oil Paint Stain | **H** | **B** | **C** | **N** | **O** | **F** | **Na** | **Mg** | **Al** | **Si** | **P** |
| | | 0.079 | 0 | 0.424 | 0.033 | 0.155 | 0 | 0 | 0 | 0.007 | 0.005 | 0 |
| | | **S** | **Cl** | **K** | **Ca** | **Ti** | **V** | **Cr** | **Mn** | **Fe** | **Co** | **Ni** |
| | | 0 | 0 | 0 | 0 | 0.075 | 0 | 0 | 0.002 | 0.039 | 0 | 0 |
| | | **Cu** | **Zn** | **Br** | **Sr** | **Zr** | **Nb** | **Mo** | **Sn** | **Ba** | **Pb** | **Bi** |
| | | 0 | 0 | 0.181 | 0 | 0 | 0 | 0 | 0 | 0 | 0 | 0 |
| Oriented Strand Board | Oriented Strand Board | **H** | **B** | **C** | **N** | **O** | **F** | **Na** | **Mg** | **Al** | **Si** | **P** |
| | | 0.061 | 0 | 0.507 | 0.002 | 0.429 | 0 | 0 | 0 | 0 | 0 | 0 |
| | | **S** | **Cl** | **K** | **Ca** | **Ti** | **V** | **Cr** | **Mn** | **Fe** | **Co** | **Ni** |
| | | 0 | 0 | 0 | 0 | 0 | 0 | 0 | 0 | 0 | 0 | 0 |
| | | **Cu** | **Zn** | **Br** | **Sr** | **Zr** | **Nb** | **Mo** | **Sn** | **Ba** | **Pb** | **Bi** |
| | | 0 | 0 | 0 | 0 | 0 | 0 | 0 | 0 | 0 | 0 | 0 |
| Paper Tape | Paper Tape | **H** | **B** | **C** | **N** | **O** | **F** | **Na** | **Mg** | **Al** | **Si** | **P** |
| | | 0.062 | 0 | 0.441 | 0 | 0.494 | 0 | 0 | 0 | 0.001 | 0 | 0 |
| | | **S** | **Cl** | **K** | **Ca** | **Ti** | **V** | **Cr** | **Mn** | **Fe** | **Co** | **Ni** |
| | | 0.001 | 0 | 0 | 0.002 | 0 | 0 | 0 | 0 | 0 | 0 | 0 |
| | | **Cu** | **Zn** | **Br** | **Sr** | **Zr** | **Nb** | **Mo** | **Sn** | **Ba** | **Pb** | **Bi** |
| | | 0 | 0 | 0 | 0 | 0 | 0 | 0 | 0 | 0 | 0 | 0 |
| Plywood | Plywood | **H** | **B** | **C** | **N** | **O** | **F** | **Na** | **Mg** | **Al** | **Si** | **P** |
| | | 0.062 | 0 | 0.504 | 0 | 0.434 | 0 | 0 | 0 | 0 | 0 | 0 |
| | | **S** | **Cl** | **K** | **Ca** | **Ti** | **V** | **Cr** | **Mn** | **Fe** | **Co** | **Ni** |

| | | | | | | | | | | | | |
|---|---|---|---|---|---|---|---|---|---|---|---|---|
| | | 0 | 0 | 0 | 0 | 0 | 0 | 0 | 0 | 0 | 0 | 0 |
| | | **Cu** | **Zn** | **Br** | **Sr** | **Zr** | **Nb** | **Mo** | **Sn** | **Ba** | **Pb** | **Bi** |
| | | 0 | 0 | 0 | 0 | 0 | 0 | 0 | 0 | 0 | 0 | 0 |
| Polystyrene Insulation | Polystyrene Insulation | **H** | **B** | **C** | **N** | **O** | **F** | **Na** | **Mg** | **Al** | **Si** | **P** |
| | | 0.077 | 0 | 0.923 | 0 | 0 | 0 | 0 | 0 | 0 | 0 | 0 |
| | | **S** | **Cl** | **K** | **Ca** | **Ti** | **V** | **Cr** | **Mn** | **Fe** | **Co** | **Ni** |
| | | 0 | 0 | 0 | 0 | 0 | 0 | 0 | 0 | 0 | 0 | 0 |
| | | **Cu** | **Zn** | **Br** | **Sr** | **Zr** | **Nb** | **Mo** | **Sn** | **Ba** | **Pb** | **Bi** |
| | | 0 | 0 | 0 | 0 | 0 | 0 | 0 | 0 | 0 | 0 | 0 |
| Roofing Asphalt | Roofing Asphalt | **H** | **B** | **C** | **N** | **O** | **F** | **Na** | **Mg** | **Al** | **Si** | **P** |
| | | 0.101 | 0 | 0.839 | 0.010 | 0.009 | 0 | 0 | 0 | 0 | 0 | 0 |
| | | **S** | **Cl** | **K** | **Ca** | **Ti** | **V** | **Cr** | **Mn** | **Fe** | **Co** | **Ni** |
| | | 0.040 | 0 | 0 | 0 | 0 | 0 | 0 | 0 | 0 | 0 | 0 |
| | | **Cu** | **Zn** | **Br** | **Sr** | **Zr** | **Nb** | **Mo** | **Sn** | **Ba** | **Pb** | **Bi** |
| | | 0 | 0 | 0 | 0 | 0 | 0 | 0 | 0 | 0 | 0 | 0 |
| Structural Steel | Steel | **H** | **B** | **C** | **N** | **O** | **F** | **Na** | **Mg** | **Al** | **Si** | **P** |
| | | 0 | 0 | 0.003 | 0 | 0 | 0 | 0 | 0 | 0 | 0.004 | 0 |
| | | **S** | **Cl** | **K** | **Ca** | **Ti** | **V** | **Cr** | **Mn** | **Fe** | **Co** | **Ni** |
| | | 0.001 | 0 | 0 | 0 | 0 | 0 | 0 | 0.014 | 0.977 | 0 | 0 |
| | | **Cu** | **Zn** | **Br** | **Sr** | **Zr** | **Nb** | **Mo** | **Sn** | **Ba** | **Pb** | **Bi** |
| | | 0.002 | 0 | 0 | 0 | 0 | 0 | 0 | 0 | 0 | 0 | 0 |
| Steel Fasteners | Steel | **H** | **B** | **C** | **N** | **O** | **F** | **Na** | **Mg** | **Al** | **Si** | **P** |
| | | 0 | 0 | 0.001 | 0 | 0 | 0 | 0 | 0 | 0 | 0.011 | 0 |
| | | **S** | **Cl** | **K** | **Ca** | **Ti** | **V** | **Cr** | **Mn** | **Fe** | **Co** | **Ni** |
| | | 0 | 0 | 0 | 0 | 0 | 0 | 0.185 | 0.020 | 0.417 | 0 | 0.355 |

| | | | | | | | | | | | | |
|---|---|---|---|---|---|---|---|---|---|---|---|---|
| | | **Cu** | **Zn** | **Br** | **Sr** | **Zr** | **Nb** | **Mo** | **Sn** | **Ba** | **Pb** | **Bi** |
| | | 0.010 | 0 | 0 | 0 | 0 | 0 | 0 | 0 | 0 | 0 | 0 |
| Galvanized Steel – Cold Rolled | Steel | **H** | **B** | **C** | **N** | **O** | **F** | **Na** | **Mg** | **Al** | **Si** | **P** |
| | | 0 | 0 | 0.001 | 0 | 0 | 0 | 0 | 0 | 0 | 0 | 0 |
| | | **S** | **Cl** | **K** | **Ca** | **Ti** | **V** | **Cr** | **Mn** | **Fe** | **Co** | **Ni** |
| | | 0 | 0 | 0 | 0 | 0 | 0 | 0.001 | 0.006 | 0.985 | 0 | 0.002 |
| | | **Cu** | **Zn** | **Br** | **Sr** | **Zr** | **Nb** | **Mo** | **Sn** | **Ba** | **Pb** | **Bi** |
| | | 0.002 | 0.001 | 0 | 0 | 0 | 0 | 0.001 | 0 | 0 | 0 | 0 |
| Rebar Steel | Steel | **H** | **B** | **C** | **N** | **O** | **F** | **Na** | **Mg** | **Al** | **Si** | **P** |
| | | 0 | 0 | 0.003 | 0 | 0 | 0 | 0 | 0 | 0 | 0.005 | 0 |
| | | **S** | **Cl** | **K** | **Ca** | **Ti** | **V** | **Cr** | **Mn** | **Fe** | **Co** | **Ni** |
| | | 0 | 0 | 0 | 0 | 0 | 0 | 0 | 0.015 | 0.976 | 0 | 0 |
| | | **Cu** | **Zn** | **Br** | **Sr** | **Zr** | **Nb** | **Mo** | **Sn** | **Ba** | **Pb** | **Bi** |
| | | 0 | 0 | 0 | 0 | 0 | 0 | 0 | 0 | 0 | 0 | 0 |
| Hot Rolled Steel | Steel | **H** | **B** | **C** | **N** | **O** | **F** | **Na** | **Mg** | **Al** | **Si** | **P** |
| | | 0 | 0 | 0.001 | 0 | 0 | 0 | 0 | 0 | 0 | 0 | 0 |
| | | **S** | **Cl** | **K** | **Ca** | **Ti** | **V** | **Cr** | **Mn** | **Fe** | **Co** | **Ni** |
| | | 0 | 0 | 0 | 0 | 0 | 0 | 0.002 | 0.006 | 0.986 | 0 | 0.002 |
| | | **Cu** | **Zn** | **Br** | **Sr** | **Zr** | **Nb** | **Mo** | **Sn** | **Ba** | **Pb** | **Bi** |
| | | 0.002 | 0 | 0 | 0 | 0 | 0 | 0.001 | 0 | 0 | 0 | 0 |
| Copper | Steel | **H** | **B** | **C** | **N** | **O** | **F** | **Na** | **Mg** | **Al** | **Si** | **P** |
| | | 0 | 0 | 0 | 0 | 0 | 0 | 0 | 0 | 0 | 0 | 0 |
| | | **S** | **Cl** | **K** | **Ca** | **Ti** | **V** | **Cr** | **Mn** | **Fe** | **Co** | **Ni** |
| | | 0 | 0 | 0 | 0 | 0 | 0 | 0 | 0 | 0 | 0 | 0 |
| | | **Cu** | **Zn** | **Br** | **Sr** | **Zr** | **Nb** | **Mo** | **Sn** | **Ba** | **Pb** | **Bi** |

| | | | | | | | | | | | | |
|---|---|---|---|---|---|---|---|---|---|---|---|---|
| | | 1.000 | 0 | 0 | 0 | 0 | 0 | 0 | 0 | 0 | 0 | 0 |
| Stone | Stone | **H** | **B** | **C** | **N** | **O** | **F** | **Na** | **Mg** | **Al** | **Si** | **P** |
| | | 0 | 0 | 0.040 | 0 | 0.454 | 0 | 0.008 | 0.015 | 0.034 | 0.223 | 0 |
| | | **S** | **Cl** | **K** | **Ca** | **Ti** | **V** | **Cr** | **Mn** | **Fe** | **Co** | **Ni** |
| | | 0 | 0 | 0.009 | 0.191 | 0.002 | 0 | 0 | 0.001 | 0.018 | 0 | 0 |
| | | **Cu** | **Zn** | **Br** | **Sr** | **Zr** | **Nb** | **Mo** | **Sn** | **Ba** | **Pb** | **Bi** |
| | | 0.002 | 0 | 0 | 0 | 0 | 0 | 0 | 0 | 0 | 0 | 0 |
| Welded Wire Mesh – Ladder Wire | Welded Wire Mesh Ladder Mesh | **H** | **B** | **C** | **N** | **O** | **F** | **Na** | **Mg** | **Al** | **Si** | **P** |
| | | 0 | 0 | 0.001 | 0.001 | 0 | 0 | 0 | 0 | 0 | 0.010 | 0 |
| | | **S** | **Cl** | **K** | **Ca** | **Ti** | **V** | **Cr** | **Mn** | **Fe** | **Co** | **Ni** |
| | | 0 | 0 | 0 | 0 | 0 | 0 | 0.200 | 0.020 | 0.662 | 0 | 0.105 |
| | | **Cu** | **Zn** | **Br** | **Sr** | **Zr** | **Nb** | **Mo** | **Sn** | **Ba** | **Pb** | **Bi** |
| | | 0 | 0 | 0 | 0 | 0 | 0 | 0 | 0 | 0 | 0 | 0 |
| Window Glass | Window Glass | **H** | **B** | **C** | **N** | **O** | **F** | **Na** | **Mg** | **Al** | **Si** | **P** |
| | | 0 | 0 | 0 | 0 | 0.462 | 0 | 0.125 | 0.022 | 0.005 | 0.344 | 0 |
| | | **S** | **Cl** | **K** | **Ca** | **Ti** | **V** | **Cr** | **Mn** | **Fe** | **Co** | **Ni** |
| | | 0 | 0 | 0.005 | 0.037 | 0 | 0 | 0 | 0 | 0 | 0 | 0 |
| | | **Cu** | **Zn** | **Br** | **Sr** | **Zr** | **Nb** | **Mo** | **Sn** | **Ba** | **Pb** | **Bi** |
| | | 0 | 0 | 0 | 0 | 0 | 0 | 0 | 0 | 0 | 0 | 0 |
| Stucco | Stucco | **H** | **B** | **C** | **N** | **O** | **F** | **Na** | **Mg** | **Al** | **Si** | **P** |
| | | 0.011 | 0 | 0.005 | 0 | 0.429 | 0 | 0.005 | 0.024 | 0.024 | 0.116 | 0 |
| | | **S** | **Cl** | **K** | **Ca** | **Ti** | **V** | **Cr** | **Mn** | **Fe** | **Co** | **Ni** |
| | | 0.004 | 0 | 0.006 | 0.360 | 0.001 | 0 | 0 | 0 | 0.013 | 0 | 0 |
| | | **Cu** | **Zn** | **Br** | **Sr** | **Zr** | **Nb** | **Mo** | **Sn** | **Ba** | **Pb** | **Bi** |
| | | 0 | 0 | 0 | 0 | 0 | 0 | 0 | 0 | 0 | 0 | 0 |

| | | | | | | | | | | | | |
|---|---|---|---|---|---|---|---|---|---|---|---|---|
| Clay | Stone Mineral Materials; Clay-kaolin | **H** | **B** | **C** | **N** | **O** | **F** | **Na** | **Mg** | **Al** | **Si** | **P** |
| | | 0.016 | 0 | 0 | 0 | 0.558 | 0 | 0 | 0 | 0.209 | 0.218 | 0 |
| | | **S** | **Cl** | **K** | **Ca** | **Ti** | **V** | **Cr** | **Mn** | **Fe** | **Co** | **Ni** |
| | | 0 | 0 | 0 | 0 | 0 | 0 | 0 | 0 | 0 | 0 | 0 |
| | | **Cu** | **Zn** | **Br** | **Sr** | **Zr** | **Nb** | **Mo** | **Sn** | **Ba** | **Pb** | **Bi** |
| | | 0 | 0 | 0 | 0 | 0 | 0 | 0 | 0 | 0 | 0 | 0 |
| Polyethylene | Polymer Materials | **H** | **B** | **C** | **N** | **O** | **F** | **Na** | **Mg** | **Al** | **Si** | **P** |
| | | 0.144 | 0 | 0.856 | 0 | 0 | 0 | 0 | 0 | 0 | 0 | 0 |
| | | **S** | **Cl** | **K** | **Ca** | **Ti** | **V** | **Cr** | **Mn** | **Fe** | **Co** | **Ni** |
| | | 0 | 0 | 0 | 0 | 0 | 0 | 0 | 0 | 0 | 0 | 0 |
| | | **Cu** | **Zn** | **Br** | **Sr** | **Zr** | **Nb** | **Mo** | **Sn** | **Ba** | **Pb** | **Bi** |
| | | 0 | 0 | 0 | 0 | 0 | 0 | 0 | 0 | 0 | 0 | 0 |
| PVC | Polymer Materials | **H** | **B** | **C** | **N** | **O** | **F** | **Na** | **Mg** | **Al** | **Si** | **P** |
| | | 0.048 | 0 | 0.384 | 0 | 0 | 0 | 0 | 0 | 0 | 0 | 0 |
| | | **S** | **Cl** | **K** | **Ca** | **Ti** | **V** | **Cr** | **Mn** | **Fe** | **Co** | **Ni** |
| | | 0 | 0.567 | 0 | 0 | 0 | 0 | 0 | 0 | 0 | 0 | 0 |
| | | **Cu** | **Zn** | **Br** | **Sr** | **Zr** | **Nb** | **Mo** | **Sn** | **Ba** | **Pb** | **Bi** |
| | | 0 | 0 | 0 | 0 | 0 | 0 | 0 | 0 | 0 | 0 | 0 |
| Plastic | Polymer Materials | **H** | **B** | **C** | **N** | **O** | **F** | **Na** | **Mg** | **Al** | **Si** | **P** |
| | | 0.089 | 0 | 0.651 | 0.015 | 0.126 | 0 | 0 | 0 | 0 | 0.047 | 0 |
| | | **S** | **Cl** | **K** | **Ca** | **Ti** | **V** | **Cr** | **Mn** | **Fe** | **Co** | **Ni** |
| | | 0 | 0.071 | 0 | 0 | 0 | 0 | 0 | 0 | 0 | 0 | 0 |
| | | **Cu** | **Zn** | **Br** | **Sr** | **Zr** | **Nb** | **Mo** | **Sn** | **Ba** | **Pb** | **Bi** |
| | | 0 | 0 | 0 | 0 | 0 | 0 | 0 | 0 | 0 | 0 | 0 |

**Table S-3.** Building material elemental composition sources and assumptions.

| Category | Material | Notes/Assumptions | Source | Sub materials (see above table for source information) |
|---|---|---|---|---|
| Aggregate | Crushed Coarse Aggregate | Assume 100% crushed limestone | | Limestone |
| | Natural Coarse Aggregate | Assume equal amounts of granite, basalt, quartzite, and limestone | | Granite, basalt, quartzite, limestone |
| | Crushed Fine Aggregate | Assume 100% crushed limestone | | Limestone |
| | Recycled Concrete Aggregate | Assume NaO2 is a typo for Na2O (https://www.mdpi.com/2075-163X/8/6/237/htm) | (Moreno-Pérez et al., 2018; Sánchez-Cotte et al., 2020) | |
| | Natural Fine Aggregate | Assume river sand - dune sand too fine for concrete | | Sand |
| Aluminum Window Frame | | Alloy number identified by industry website; Alloy composition from ASTM B221; assume max % for each element range, remove 'others' category and re-weight | ("Aluminium Profiles for Windows and Doors Manufacturer & Supplier," n.d.; B07 Committee, n.d.) | |
| Asphalt Shingles | | Asphalt shingle roofing system EPD by the Asphalt Roofing Manufacturers Association; ignore <1% items; assume "laminating adhesive" is just asphalt - as said in this source; Assume "mineral granules" are natural course aggregate; Assume "mineral stabilizers" are limestone | (*Asphalt Roofing Shingles Into Energy Project Summary Report*, 2008; *Asphalt Shingle Roofing System, Installation: Fastened*, 2016) | Limestone, natural coarse aggregate, Roofing asphalt, natural fine aggregate, Fiberglass batt |
| Brick | | Make assumption that the remainder of weight is oxygen because most/all of these | (*Standard Reference Material 679* | |

| | | | |
|---|---|---|---|
| | elements are oxides in natural form | *Brick Clay*, 1987) | |
| Carpet | Assume 'additives' = 0 b/c no way to tell exactly what they are; assume 'biocide, pigment, dye, stabilizer' = 0 b/c small and do not know details; assume 'basic aluminosilicates' = kaolin | (*AtlasMasland - Modular Carpet Family*, 2020; *Carpet Roll*, 2021; *Tufted broadloom carpet - luxury class LC1-LC5 - with 1200 g/m2 maximum surface pile weight - pile material made of polyamide 6, textile backing*, 2016; *Tufted carpet tiles - luxury class LC1-LC5 - with 1200 g/m2 maximum surface pile weight - pile material made of polyamide 6.6, bitumen based heavy backing*, 2016) | Nylon, polyamide; Polyester felt (PET); VAE; Vinyl (PVC), Fiberglass loose fill |
| Cellulose Insulation | Source 1: North American Industry wide EDP, | (Raclavská et al., 2021b) | Softwood |

| | | | | |
|---|---|---|---|---|
| | | Conventional loose fill cellulose insulation<br>Source 2:<br>Assume waste paper etc. category = ave of cellulose, paper board, waste office paper (latter 2 are trace elements with the remaining = cellulose);<br>Assume cardboard and fiber residuals are only wood pulp = softwood | 2/3/2026 12:28:53 PM | |
| Cementitious Materials | Cement | Just take number, ignore ±<br>CO2 taken from LOI between 550 and 950C - ignore other LOI, Free CaO category (already CaO from other method included), sulfide (already SO3 from other method) insoluble residue | (*Standard Reference Material 1885b Portland Cement*, 2013) | |
| | Mortar | ASTM C1329 - Standard Specification for Mortar Cement for use where mortar masonry is required;<br>Take average of N, S, M types;<br>Assume w/cm = 0.5;<br>Assume sand is natural fine aggregate | (C01 Committee, n.d.) | Natural fine aggregate; cement |
| | Concrete | Appendix B: Mix design specifications and raw material quantitites per 1 cubic yard ready mix concrete; assumed to be 5000 psi with 40% fly ash;<br>don't incorporate admixtures (~1 oz added to 1 cubic yard of concrete;<br>w/cm = 0.35; | (The Athena Sustainable Materials Institute, n.d.) | Cement; Coal fly ash; Slag – GGBFS; Crushed coarse aggregate, natural coarse aggregate, crushed fine aggregate, natural fine aggregate |

| | | | | |
|---|---|---|---|---|
| | | air entrainment yes for all | | |
| | Stucco | ASTM C926-23a - Standard Specification for Application of Portland Cement-Based Plaster;<br>Assume sand is natural fine aggregate;<br>Assume 1 m3 of material for weight proportioning | (C11 Committee, n.d.; Holcim US, 2022) | Cement; Hydrated lime, natural fine aggregate |
| Ceramic Tiles | | Industry wide EPD of ceramic tiles produced in USA;<br>assume ash is coal fly ash (likely coal heating the kilns);<br>Assume additives are Lignin (biopolymers seem to be a common additive) see plywood sheet | (*North America Ceramic Tile: Porcelain, Pressed Floor, Mosaic Quarry, Glazed Wall*, 2020) | Clay – Kaolin; Sand; Talc; Feldspar; Frit; Coal fly ash; lignin; Ink; Glaze |
| Cross Laminated Timber | | softwood lumber with PUR or PRF resins; assume half PUR and half PRF | (*Crosslam CLT: EPD for Cross Laminated Timber produced by Structurlam in Okanagan Falls, BC*, 2020) | Softwood; Polyurethane resin (PUR); Phenol-resorcinol-formaldehyde |
| Dimensional Lumber, Window Frames | | | (*North American Softwood Lumber*, 2020) | Softwood |
| EPDM | | Solely EPDM polymer: Ethylene Propylene Diene Terpolymer | ("Polymeric Geomembrane Components in Landfill Liners - ScienceDirect," n.d.) | Polyethylene; Polypropylene, APP; Diene polymers |

| | | | |
|---|---|---|---|
| Fiber Cement | Source 1:<br>EPD for JamesHardie company in USA<br>Source 2:<br>Cembrit EPD - Cembrit functional boards<br>Source 3:<br>Equitone Linea/Lunara fibre cement sheets ETEX<br>Source 4:<br>Swisspearl Group AG - large-size fibre cement plates Swisspearl<br>Source 5:<br>Weinberger SNK fibre cement flat sheets<br>Source 6:<br>ETEX Natura, Textura, and Materia fiber-cement panels,<br>assume equal weighting of polypropylene, nylon, and polyethylene,<br>assume just water (dye is likely small amount)<br><br>all: assume w/cm = 0.3 | (*Cembrit functional boards*, 2016; *Environmental Product Declaration Fibercement Cladding*, 2019; *EQUITONE [Linea / Lunara] fibre cement sheets*, 2020; *Large-size fibre cement plates Swisspearl*, 2018; *NATURA, TEXTURA and MATERIA Fiber-Cement Panels*, n.d.)<br><br>https://www.wienerberger.co.uk/content/dam/wienerberger/united-kingdom/marketing/documents-magazines/technical/svk-technical-documents/UK_MKT_DOC_TEC_FAC_SVK_Environmental_P | C-S-H; Cellulose; C-A-S-H; Polyacrylates – Acrylics; Limestone; Mica; PVA; Natural fine aggregate; pigments; trass; wollastonite; clay- Kaolin; Softwood; Polyethylene; Silica fume; Nylon – polyamide; Polypropylene, APP |

| | | | |
|---|---|---|---|
| | | roduct_Declaration.pdf | |
| Fiberglass Batt | | (Berdan II and Bertram, n.d.) | Glass cullet; limestone; binder coatings; sand |
| Fiberglass Loose Fill | | (Berdan II and Bertram, n.d.) | Glass cullet; limestone; binder coatings; sand |
| Fiberglass Window Frame | Wagners Fiber reinforced plastic for structural protrusion specification - composite fiber technologies ;<br>Assume equal amounts of two resins | (Wagners CFT Manufacturing Pty Ltd, n.d.) | ECR glass fibers; Vinyl resins: bispheol-A-epoxy esters; Vinyl resins: epoxy novolac vinyl ester |
| Fire-rated Type X Gypsum Board | Ignore anything with 0.08% or less;<br>Ignore dispersants and soaps b/c complex and variable compositions;<br>Ignore ball mill accelerator (BMA) b/c complex and variable compositions | (*Industry Average EPD for 5/8" Type X Conventional Gypsum Board*, 2020; Savoly and Elko, 2015) | Fiberglass batt; Gypsum; Cellulose; Amylum, Starch, Cornstarch |
| Polyurethane Foam Insulation | Two sides are equal volumes;<br>Density of side A = 0.022-0.026 g/cm3, go with 0.024 g/cm3 average;<br>overall density of the PU foam insulation = 2 lb/ft3 = 0.032 g/cm3;<br>by rule of mixtures, the density of side B (when vol% of each = 50%) = 0.04 g/cm3;<br>assume 1cm3 volume = 37.5 wt% side A, 62.5 wt% side B;<br>Source 3: Assume compatibilizer is | (Lesage et al., 2007; *Spray polyurethane foam insulation (HFO/HFC)*, 2022; "The chemistry of polyurethanes," n.d.) | Methylene diphenyl diisocyanate (MDI); Polyester felt (PET); Mannich base; TCPP; Silicone |

| | | | |
|---|---|---|---|
| | lignocellulose, which assume is softwood; Source 4: List of various metal catalysts - assume average of all | | |
| Glass Mat Gypsum Panel 5/8" | Ignore anything less than 0.08% Ignore dispersants and soaps b/c complex and variable compositions | (*Industry-wide cradle-to-gate EPD for 1 /2” and 5 /8” Glass-mat Gypsum Boards*, 2021; Savoly and Elko, 2015) | Fiberglass batt; Gypsum; Silicone |
| Glued Laminated Timber | Softwood with Phenol-resorcinol-formaldehyde (PRF) resin | (*North American Glue Laminated Timber*, 2020; Puettmann and Wilson, n.d.) | Softwood; Phenol-resorcinol-formaldehyde (PRF) resin |
| Gypsum Board / Moisture Resistant Gypsum Board | ASTM says it has to be at least 70% gypsum (CaSO4.2H2O); Ignore 'others' category; fiberglass' = fiberglass batt; assume paper = cellulose | https://portal.environdec.com/api/api/v1/EPDLibrary/Files/97c7687f-85f7-4f5b-97f6-e555486f561c/Data | Gypsum; Fiberglass batt; Amylum, Starch, Cornstarch; Cellulose |
| Heraklith | Source 1: MSDS - composition %; assume wood spruce is softwood; Source 2: Info brochure: binder = Portland cement | (Knauf Insulation, 2013) | Softwood; Cement |

| | | | |
|---|---|---|---|
| | assume binder = Portland cement with w/cm = 0.5 | | |
| Joint Compound | Ignore <, > signs; change limestone to 75 because it says >65 and the rest say < | (United States Gypsum Company, 2014) | Limestone; Talc; Attapulgite; Mica; Perlite; Clay - Kaolin |
| Latex Paint | NIST generic latex paint products report | (Darling, 2005) | Vinyl resin (vinyl acrylic, polyvinyl acrylic, styrene acrylic); limestone |
| Linoleum | Source 1:<br>EPD Forbo Marmoleum ; assume 'polyolefin foam' is same as polyolefin film = PE<br>Source 2:<br>EPD Gerflor Linoleum compact ; assume 'oils' = half linseed and half soybean, assume 'wood flour' is softwood<br>Source 3:<br>EPD Gerflor Linoleum compact ; assume 'oils' = half linseed and half soybean, assume 'wood flour' is softwood<br><br>all sources here are for linoleum flooring (all EPDs for specific products, no industry average that I could find)<br>group 3 pigment categories together | (*DLW Linoleum Acousticplus Gerford DLW Linoleum Acousticplus Sheet Flooring*, 2020; *DLW Linoleum Compact Gerflor DLW Linoleum Compact Sheet Flooring*, 2020; *MARMOLEUM DECIBEL FORBO FLOORING SYSTEMS RESILIENT LINOLEUM FLOOR COVERING*, 2018) | Linseed oil; Gum rosin; Tall oil; Softwood; Jute – Hessian; Film – polyolefin; Lacquer varnish; Pigment; Epoxidized soybean oil; limestone; Polyurethane foam |
| Metal Roof Cladding | Assume 50% galvalized steel = ASTM A653 CS type A, and 50% galvalume steel = | ("15 USC 206: Standard gauge for sheet and | |

| | | | |
|---|---|---|---|
| | ASTM A792 CS type B with coating; CS type A and CS type B are the same; From US law code about steel coating thicknesses; 26 gauge is the most common thickness for steel roof cladding ; 0.47625 mm thickness | plate iron and steel," n.d.; A05 Committee, n.d., n.d.) | |
| Mineral Fill, Mineral Wool Loose Fill | North American Insulation Manufacturers Association EPD; Mineral wool loose | (*Mineral Wool Loose*, 2013) | Slag; Bauxite; Granite; Feldspar; PEG |
| Mineral Wool, Rockwool | Source 1: North American Insulation Manufacturers Association – EPD, This assumes mineral wool is a combo of both slag and rock wool, Assume 50% light density insulation board and 50% heavy density insulation board<br><br>Source 2: This other EPD for rockwool insulation is also mostly slag, so assume they are the same general one given by the NAIMA, Ignore 'other binder', assume the <1 for everything else is 0.5 | (*Mineral Wool Board*, 2013)<br><br>https://portal.environdec.com/api/api/v1/EPDLibrary/Files/7c773f18-b0ad-4e16-80f8-d5cc9b59e6e4/Data | Slag; Basalt; Feldspar; Cement; Granite; Phenolic resin |
| Modified Bitumen Membrane (Styrene-butadiene-styrene (SBS) and Atactic polypropylene Modified Bitumen Membrane (APP average)) | Source 1: SBS-modified - assume the propane torch Installation method b/c most contained material system and wt% s given; assume 50% of | (*SBS-MODIFIED BITUMEN ROOFING MEMBRANE INSTALLATION: TORCH* | Roofing asphalt; Natural coarse aggregate; limestone; Polyester felt (PET); fiberglass batt; colemanite; SBS, SBR latex; natural fine aggregate; film-polyolefin; polypropylene, APP; polyethylene |

| | | | |
|---|---|---|---|
| | each when written "polyester/fiberglass mat"<br><br>Source 2: assume 50% of each when written "polyester/fiberglass mat" | *APPLIED*, 2018) | |
| Oil Paint/Stain | Zar Oil Based Wood Stain, Black Walnut-125-Old Product; Varathane Premium, Fast Dry Wood Stain, Oil Based, Sunbleached-12/22/2015; Pubchem for formulas | ("CPID," n.d.; Kim et al., 2023) | Linseed oil, polymer with maleic anhydride and pentaerythritol; Naptha Stoddard medium aliphatic; petroleum distillates (JP5 jet fuel); Umber; Naptha light aromatic |
| Oriented Strand Board | Softwood with resin (PF or pMDI) | (*North American Oriented Strand Board*, 2020; Rowell, 2012; Yuan et al., 2021) | Softwood; Phenolic resin; MDI, pMDI |
| Paper Tape | Assume this is joint tape for drywall | (United States Gypsum Conmpany, 2003) | Cellulose; limestone |
| Plywood | Softwood (firs, pines, coniferous) with phenol-formaldehyde resin | (*North American Softwood Plywood*, 2020; Rowell, 2012) | Softwood; Phenolic resin |
| Polystyrene Insulation | Assume 100% polystyrene | | Polystyrene |
| Roofing Asphalt | Source 1:<br>Varying composition - made from crude oil, so composition depends | ("Asphalt (Bitumen) (Cicads 59, 2004)," n.d.; | |

| | | | | |
|---|---|---|---|---|
| | | on where the crude oil came from<br>Source 3: made assumptions on what "small amounts" meant | "WHAT'S THAT STUFF? - Asphalt," n.d.; Petersen, n.d.) | |
| Steel | Structural Steel | Max element % from ASTM used | (A01 Committee, n.d.) | |
| | Steel Fasteners | 330 stainless steel<br>Max element % from ASTM used; if range given average of range used | (B02 Committee, n.d.) | |
| | Galvanized Steel – Cold Rolled | Assume same galvanzied steel coating thickness as used for metal roof cladding | (A05 Committee, n.d.) | Metal roof cladding |
| | Rebar Steel | Max element % from ASTM used | (A01 Committee, n.d.) | |
| | Hot Rolled Steel | Assume CS type A<br>No limit specified for Al, Si, N, B - just ignore b/c no way to tell how much accurately | (A01 Committee, n.d.) | |
| | Copper | Assume 100% Cu metal | | |
| Stone | | Assume this is a weighted combo of some common building stone options according to this report, common stones are granite, limestone, marble, sandstone, slate, travertine, quartzite | PROPERTIESOFBUILDINGSTONES.pdf | Granite, limestone, marble, sandstone, slate, travertine, quartzite |

| | | | |
|---|---|---|---|
| Welded Wire Mesh, Ladder Wire | Source 1: General info and standards ID from National Concrete Masonry Association (NCMA) Source 2: Type of steel ID'd in ASTM A951 Source 3: Steel composition (304) given in ASTM A580 assume max % given for each elemental range | (A01 Committee, n.d., n.d., "Joint Reinforcement for Concrete Masonry," n.d.) | |
| Window Glass | General formula for soda lime glass used for bottles and windows from Corning Museum of Glass website | ("Soda-lime glass \| All About Glass," n.d.) | |

**Table S-4.** Building sub-material elemental composition sources and assumptions.

| Category | Material | Notes/Assumptions | Source(s) |
|---|---|---|---|
| Stone-mineral-materials | Hydrated lime | Hydrated limebarnard (type S) specified in ASTM C207, use Holcim SDS as composition estimates | (C07 Committee, n.d.; Holcim US, 2022) |
| | Limestone | NIST SRM 1d: agrillaceous limestone | (*Standard Reference Material 1d Limestone, Argillaceous*, 2005) |
| | Gypsum | | (Dana, 1985) |
| | Umber | assume n=1 | ("Pigments through the Ages - Technical Information - Umber," n.d.) |
| | Basalt | | (Korkanç and Tugrul, 2004) (Kim et al., 2019a) ("Chemical composition of basalt aggregate, quartz sand and |

| | | | |
|---|---|---|---|
| | | | WFS (in %).," n.d.)<br><br>(Al-Akhaly, 2018)<br><br>("Chemical Composition of Basaltic aggregate [3]," n.d.) |
| | Slag – ground granulated blast furnace slag (GGBFS) | | (El-Chabib, 2020; Siddique and Cachim, 2018) |
| | Clay - Kaolin | found on chemical book by searching CAS number given in USG source given here | (United States Gypsum Company, 2023)<br><br>("ChemicalBook, " 2023) |
| | Talc | found on chemical book by searching CAS number given in USG source given here | (United States Gypsum Company, 2023)<br><br>("ChemicalBook, " 2023) |
| | Attapulgite | found on chemical book by searching CAS number given in USG source given here | (United States Gypsum Company, 2023)<br><br>("ChemicalBook, " 2023) |
| | Mica | found on chemical book by searching CAS number given in USG source given here | (United States Gypsum Company, 2023)<br><br>("ChemicalBook, " 2023) |
| | Perlite | | (Kim et al., 2023) |
| | Frit | Assume transparent frit | ("The Chemistry, Physics and Manufacturing of Glaze Frits," n.d.) |
| | Ink | assume general blue ink because composition easily accessible; will vary based on color | (CoorsTek, 2017) |
| | Travertine | Source 1: average of some travertines | (Fereidooni and Khajevand, 2018; |

| | | | |
|---|---|---|---|
| | | Both sources: assume "other" = MgCO3 b/c it was an example listed | Nimfopoulos et al., 2002) |
| | Marble | Source 1: average Egyptian marble | (Fakhry et al., 2017; Fares et al., 2011; Sariisik, 2012; Zumrawi and Abdalla, 2018) |
| | Quartzite | | (Abbood, 2018; Cala et al., 2019; Kim et al., 2019b; Tufail et al., 2017a) |
| | Silica fume | Just take number, ignore ± | (*Standard Reference Material 2696 Silica Fume (powder form)*, 2017) |
| | Cement | Just take number, ignore ±<br>CO2 taken from LOI between 550 and 950C - ignore other LOI, Free CaO category (already CaO from other method included), sulfide (already SO3 from other method) insoluble residue | (*Standard Reference Material 1885b Portland Cement*, 2013) |
| | C-S-H | | ("Calcium silicate hydrate \| chemical compound \| Britannica," 2024) |
| | C-A-S-H | Assume naturally occuring mineral Tacharanite | ("Tacharanite Mineral Data," n.d.) |
| | Pigment | Assume equal weighting of TiO2, Fe2O3, Cr2O3 | |
| | Sandstone | Source 1:<br>mid of ranges given for average sandstone<br>Source 2:<br>EDXRF sandstone rock<br>Source 4:<br>Average of 9 samples | ("Chemical Properties of Sandstone,Sandstone Chemical Properties,Chemical Composition of Sandstone," n.d.)<br><br>(Fořt, 2015; Kyzs'michev et al., 2006; Mubiayi, 2014) |
| | Slate | Source 1:<br>middish value for range given - to get to 100%, not quite mid for all<br>Source 3: | ("Chemical Properties Of Slatestone,Slatest |

| | | | |
|---|---|---|---|
| | | industry website | one Chemical Properties,Chemical Composition of Slatestone," n.d.)<br><br>("Industrial Minerals \| Slate Powder \| Slate Granules \| Delabole Slate," n.d.; Rodriguez et al., 1997; Wintsch et al., 1991) |
| | Glaze | assume general formula (lots of variations possible): 0.2K2O, 0.1NaO, 0.7CaO)•0.5Al2O3•4.0SiO2 | ("Techno File," n.d.) |
| | Granite | | (Kim et al., 2019a)<br><br>("Home \| Reade Advanced Materials," n.d.)<br><br>(Tufail et al., 2017b) 2/3/2026 12:28:53 PM |
| | Sand | Source 1: Assume river sand b/c used for concrete | (Makani et al., 2010)<br><br>(Taylor et al., 2012) |
| | Colemanite | | (Kim et al., 2023) |
| | Coal fly ash | elements listed; assume remaining is CO2 that burned out<br>Just take number, ignore ± | (*Standard Reference Material 2690 Coal Fly Ash*, 2015) |
| | Feldspar | Assume 33% of each KAlSi3O8, NaAlSi3O8, CaAl2Si2O8 | ("Feldspar - Mineral Composition, Uses, Types \| Britannica," 2024) |
| | Wollastonite | | (Andrews and Division, 1970) |
| | Bauxite | | (*Standard Reference Material 69b* |

| | | | |
|---|---|---|---|
| | | | *Bauxite (Arkansas)*, 1991) |
| Natural materials | Amylum, starch, cornstarch | | (Institution, n.d.; Kim et al., 2023) |
| | Paraffin wax | | (“ChemicalBook, ” 2023) |
| | Lignin | | (Kim et al., 2023) |
| | Jute-Hessian | assume water soluble materials = mostly water so just H2O | (“JUTE FIBRE, CHEMICAL COMPOSITION OF JUTE FIBRE, CHEMICAL FORMULA OF JUTE FIBRE,” n.d.; Kim et al., 2023) |
| | Softwood | ignore "ash" contribution, so small and undefined | (*North American Softwood Plywood*, 2020, p. 1; Rowell et al., 2012; U.S. Environmental Protection Agency, 2016) |
| | Hemi-cellulose | | (Rodriguez Alonso, 2015) |
| | Cellulose | Assume n = 1 | (Chen, 2014) |
| | Trass | | (Kocak, 2016; Moazenian, 2017; Risdanareni et al., 2014) |
| | Goat hair | | https://www.barnardhealth.us/amino-acids-2/mohair-biology-and-characteristics.html |
| | Waste paper | assume average of cellulose, paper board, and waste office paper | (Raclavská et al., 2021a) |
| | Glass cullet | Source 1: recycled soda lime glass | (“Properties of inorganic polymer (geopolymer) mortars made of |

| | | | |
|---|---|---|---|
| | | | glass cullet \| Journal of Materials Science," n.d.; "See our Glass Cullet properties," n.d.) |
| Polymer materials | "plastic" | General category - take average other plastics: PVC, polyethylene, polystyrene, polypropylene, acrylic, PET, nylon 6, silicone | |
| | Nylon, Polyamide | assume n = 1 | ("Nylon 6 vs. nylon 6/6," n.d.) |
| | Silicone | Assume just PDMS because this is the most basic, and lots of variations to CH3 group make different properties for different applications | http://polymerdatabase.com/polymer%20classes/Silicone%20type.html<br><br>(Kim et al., 2023) |
| | Polyester felt (PET) | Assume polyester, PET | (Van der Vegt and Govaert, 2005) |
| | Polyacrylates, Acrylics | assume all acrylic, polyacrylate polymers are PMMA<br>assume n = 1 | (Kim et al., 2023) |
| | Vinyl (PVC) | | ("CAS Common Chemistry," n.d.) |
| | Polyethylene | | ("ChemicalBook, " 2023) |
| | Polystyrene | | (Singh Jadaun et al., 2022) |
| | Polypropylene, APP | | (Kim et al., 2023) |
| | Polyvinyl alcohol (PVA) | | ("POLYVINYL ALCOHOL \| CAMEO Chemicals \| NOAA," n.d.) |
| | Polyethylene glycol (PEG) | | (Kim et al., 2023) |
| | Polypropylene glycol (PPG) | | (Kim et al., 2023) |
| | VAE | Assume ethylene vinyl acetate | (Kim et al., 2023) |
| | Tris Chloroisopropyl Phosphate (TCPP) | | ("Tris (chloroisopropyl) phosphate (TCPP) - The Chemical Company," n.d.) |
| | ECR glass fibers | | (D13 Committee, n.d.) |
| | Rayon - viscose | | ("ChemicalBook, " 2023) |
| | dioctyl terephthalate | | (Kim et al., 2023) |

| | | | |
|---|---|---|---|
| | Styrene-butadiene-styrene (SBS), SBR-latex | | (“SBS mixture,” n.d.) |
| | Binder coatings | assume even amounts of each constituent b/c don't have weightings, just materials used; PubChem for consistent formulas; phenol-formaldehyde resin and lignin pulled from this table | (Kim et al., 2023; U.S. Environmental Protection Agency, 2016) |
| | Diene polymers | Assume EPDM polymer material | (“Polymeric Geomembrane Components in Landfill Liners - ScienceDirect,” n.d.) |
| | Film-polyolefin | assume polyethylene film – see polyethylene in this table | (Research, 2023) |
| | MDI, pMDI , polymeric methylene di-isocyanate | | (Kim et al., 2023) |
| | Polyester polyol | assume this is the formula because there will be lots of this (n repeat units), the remaining C2H6O3 will end up being minor amounts in comparison | (“Polyols \| Polyester Polyols \| Supplier & Distributor,” 2019) |
| | potassium 2-ethyl hexanoate | assume no H2O (not hydrated) | (“3164-85-0, 182.3, Potassium 2-Ethylhexanoate - 49E820\|E0121-500G - Grainger,” n.d.) |
| | Pentane | | (Kim et al., 2023) |
| Resins | Organic silicone resin (silicone) | RnSiXmOy, assume methyl group as most simple = (CH2)2 Si O | https://en.wikipedia.org/wiki/Silicone_resin |
| | Stryene-acrylic | | (Darling, 2005) |
| | Polyester resin (PET) | assume just polyester, PET | (Van der Vegt and Govaert, 2005) |
| | Vinyl Resin: vinyl ester | two types of vinyl ester resin: bispheol-A-epoxy esters, epoxy novolac vinyl ester, structural depictions given in this polymer database source, chemical formula taken from counting up these structural depictions, assume just the repeat unit and ignore end groups (will be pretty low percentage anyway) | https://polymerdatabase.com/polymer%20classes/Epoxy%20Vinyl%20ester%20type.html |
| | Vinyl Resin: vinyl acrylic | | (Darling, 2005) |
| | Vinyl Resin: polyvinyl acrylic | | (Darling, 2005) |

| | | | |
|---|---|---|---|
| | Polyurethane resin (PUR) | | https://www.worldofchemicals.com/chemicals/chemical-properties/polyurethane.html |
| | Phenol-resorcinol-formaldehyde (PRF) | | ("ChemicalBook," 2023) |
| | Gum rosin | | (Kim et al., 2023) |
| | Epoxy resin | | (Kim et al., 2023) |
| | Phenolic resin, Phenol-Formaldehyde resin | | (Kim et al., 2023) |
| Oil materials | Tall oil | image of the approximate structure - counted by hand | ("ChemicalBook," 2023; Shivokevich and Weinberg, 2021) |
| | Neutrals | "Neutrals may include a wide range of chemical compounds, although alkanes (hydrocarbons), steroid-type compounds, ketones, aldehydes, alcohols, mercaptans, and salts have all been found within the neutral class of substances in tall oil"; assume 'salts' = NaCl, 'mercaptan' = methanethiol, 'alcohols' = ethanol | (Shivokevich and Weinberg, 2021) |
| | Fatty acids | | (Shivokevich and Weinberg, 2021) |
| | Rosin acids | | (Shivokevich and Weinberg, 2021) |
| | Mannich base | | (Kim et al., 2023) |
| | Caprolactam | | (Kim et al., 2023) |
| | Linseed oil | formulas from Pubchem | (Kim et al., 2023; Rwahwire et al., 2019) |
| | Lacquer, Varnish | main component is urushiol - assume 100%<br>formula from Pubchem | (Kim et al., 2023; Lu and Miyakoshi, 2015) |
| | Linseed oil polymer with maleic anhydride | | ("Linseed oil, polymer with maleic anhydride, pentaerythritol and polyethylene glycol ether with glyce - 127970-91-6 \| Vulcanchem," n.d.) |
| | Naptha, Stoddard solvent, medium aliphatic | | ("Stoddard Solvent, Thermo Scientific |

| | | | |
|---|---|---|---|
| | | | Chemicals, Quantity: 1 kg \| Fisher Scientific," n.d.) |
| | Naptha light aromatic | | ("Solvent naphtha (petroleum), light arom.," n.d.) |
| | Petroleum distillates, JP5 | assume C9-C16 paraffins are half C10 and half C16;<br>assume cycloparaffins = cyclohexane;<br>assume 'aromatics' = benzene;<br>assume olefins are the same as cycloparaffin | (Fuels, 1996; Kim et al., 2023) |
| | Epoxidized soybean oil | | (Kim et al., 2023) |

# 3. Worked Examples to Demonstrate AUI Results

This subsection provides the equations and assumptions used in the case studies of CO2 mineralization, biogenic carbon sequestration, and metal (iron) supply chain estimates for a modern cell phone.

## 3.1. $CO_2$ Mineralization (Calcination)

This calculation assumed a stoichiometric basis for calcium carbonate:

$$Ca^{2+} + CO_3^{2-} \xrightarrow{yields} CACO_3.$$

From this reaction, one mole of carbon dioxide ($CO_2$) reacts with calcium (Ca) to form one mole of calcium carbonate ($CaCO_3$). Calcium carbonate has a molar mass of approximately 100.09 g/mol and $CO_2$ has a molar mass of 44.01 g/mol.

Thus, to sequester 1 tonne, or 1000 kg, of $CO_2$:

$$\frac{1000000\ g}{44.01\ g/mol} = \sim 22{,}722\ mol\ CO_2, \text{ which results in } \sim 22{,}722\ mol\ CaCO_3.$$

Therefore, this calcination process results in:

$$\frac{22722\ mol\ CaCO_3}{100.09\ g/mol} = \sim 910000\ g \text{ of Ca, or } 910\ kg \text{ of Ca to sequester 1 tonne of } CO_2.$$

The median AUI for Ca is 4214 mol/$m^2$, or 169 kg/$m^2$. Therefore,

$$\frac{169\ kg/m^2}{910\ kg\ per\ 1\ tonne\ of\ CO_2} = \sim 0.186\ tonne/m^2 \text{ of } CO_2 \text{ sequestered.}$$

Thus, given the AUI of Ca, the floor area of a building to sequester 1 tonne of $CO_2$ must be:

$$\frac{1\ tonne\ of\ CO_2}{0.186\ tonne\ of\ CO_2/m^2} = \sim 5.4\ m^2$$

This is a theoretical stoichiometry calculation. The actual process will have inefficiencies, side reactions, and incomplete conversions.

## 3.2. Biogenic Carbon Storage

This calculation also assumed a stoichiometric basis for the carbon dioxide ($CO_2$) sequestered. Given that the AUI of oxygen in buildings is significantly higher than the other scientific elements, the AUI of carbon (C) would control the amount of $CO_2$ sequestered. The median AUI for C is 6921 mol/$m^2$, or 83 kg/$m^2$. Given that $CO_2$ has a molar mass of 44.01 g/mol:

$$\frac{44.01\ \frac{g}{mol}\ of\ CO_2}{12.01\ \frac{g}{mol}\ of\ C} = 3.67 \text{ conversion factor. Thus, } 83\frac{kg}{m^2} of\ C \times 3.67 = 305\ kgCO_2/m^2$$

This assumes that the $CO_2$ stored is stable mineral carbon.

## 3.3. Iron Supply Chain for Electronics Example

This calculation assumed that the amount of iron (Fe) required to produce a cell phone is 30 g per cell phone (see Christian et al. (2014), "Elemental compositions of over 80 cell phones" and Bookhagen et al. (2020), "Metallic resources in smartphones."). Although the amount of iron varies based on the model of the phone, 30 grams is a conservative estimate.

In the present study, the median AUI for Fe is 857 mol/$m^2$, or 48 kg/$m^2$. The Fe extracted from buildings could thus be used for cell phone manufacturing.

$$\frac{48\,\frac{kg}{m^2}\ of\ Fe}{0.03\ kg\ of\ Fe\ per\ phone} = 1600\ cell\ phones\ per\ m^2.$$

Theoretically, the iron extracted from buildings could be used to manufacture 1600 cell phones per $m^2$. It must be emphasized that many other elements and minerals are used to manufacture cell phones. Yet this quick calculation demonstrates that elemental recovery of iron can indeed support the demand of iron from other sectors, like electronics.

# 4. References


15 USC 206: Standard gauge for sheet and plate iron and steel [WWW Document], n.d. URL https://uscode.house.gov/view.xhtml?req=granuleid:USC-prelim-title15-section206&num=0&edition=prelim (accessed 10.3.24).

3164-85-0, 182.3, Potassium 2-Ethylhexanoate - 49E820|E0121-500G - Grainger [WWW Document], n.d. URL https://www.grainger.com/product/SPECTRUM-Potassium-2-Ethylhexanoate-49E820?gucid=N:N:PS:Paid:GGL:CSM-2296:9JMEDM:20500731&gclid=Cj0KCQjwoK2mBhDzARIsADGbjeokbOJLDWJDfcAKRRSBP8I32tsMOb0apz5dCRzxcGp93w7jr_wAlqQaApUTEALw_wcB&gclsrc=aw.ds (accessed 10.3.24).

A01 Committee, n.d. Specification for High-Strength Carbon-Manganese Steel of Structural Quality. https://doi.org/10.1520/A0529_A0529M-19

A01 Committee, n.d. Specification for Deformed and Plain Low-Alloy Steel Bars for Concrete Reinforcement. https://doi.org/10.1520/A0706_A0706M-24

A01 Committee, n.d. Specification for Steel, Sheet and Strip, Hot-Rolled, Carbon, Structural, High-Strength Low-Alloy, High-Strength Low-Alloy with Improved Formability, and Ultra-High Strength. https://doi.org/10.1520/A1011_A1011M-23

A01 Committee, n.d. Specification for Steel Wire for Masonry Joint Reinforcement. https://doi.org/10.1520/A0951_A0951M-16E01

A01 Committee, n.d. Specification for Stainless Steel Wire. https://doi.org/10.1520/A0580_A0580M-18

A05 Committee, n.d. Specification for Steel Sheet, Zinc-Coated (Galvanized) or Zinc-Iron Alloy-Coated (Galvannealed) by the Hot-Dip Process. https://doi.org/10.1520/A0653_A0653M-23

A05 Committee, n.d. Specification for Steel Sheet, 55% Aluminum-Zinc Alloy-Coated by the Hot-Dip Process. https://doi.org/10.1520/A0792_A0792M-23

Abbood, A.A., 2018. Performance of Self-Compacting Concrete Slab with Grinded Local Rocks. jcoeng 24, 88–97. https://doi.org/10.31026/j.eng.2018.10.07

Al-Akhaly, İ.A., 2018. Engineering Properties of Basalt Coarse Aggregates in Hamdan Area, NW Sana'a, Yemen. Jeoloji Mühendisliği Dergisi 42, 159–174. https://doi.org/10.24232/jmd.486021

Aluminium Profiles for Windows and Doors Manufacturer & Supplier [WWW Document], n.d. URL https://www.wellste.com/aluminium-profiles-for-windows-doors/ (accessed 10.3.24).

Andrews, R.W., Division, I. of G.S. (Great B.M.R., 1970. Wollastonite. H.M. Stationery Office.

Asphalt (Bitumen) (Cicads 59, 2004) [WWW Document], n.d. URL https://inchem.org/documents/cicads/cicads/cicad59.htm#3.1 (accessed 10.3.24).

Asphalt Roofing Shingles Into Energy Project Summary Report (No. DOE/GO86009-Final), 2008. . Owens Corning, Toledo, OH. https://doi.org/10.2172/927606

Asphalt Shingle Roofing System, Installation: Fastened (Environmental Product Declaration), 2016. . Asphalt Roofing Manufacturers Association.

AtlasMasland - Modular Carpet Family (Environmental Product Declaration), 2020. . AtlasMasland.

B02 Committee, n.d. Specification for Nickel-Iron-Chromium-Silicon Alloys (UNS N08330 and N08332) Plate, Sheet, and Strip. https://doi.org/10.1520/B0536-19

B07 Committee, n.d. Specification for Aluminum and Aluminum-Alloy Extruded Bars, Rods, Wire, Profiles, and Tubes. https://doi.org/10.1520/B0221-21

Berdan II, C., Bertram, P.R., n.d. Generic Fiberglass. National Institute of Standards and Technology.

C01 Committee, n.d. Specification for Mortar Cement. https://doi.org/10.1520/C1329_C1329M-16A

C07 Committee, n.d. Specification for Hydrated Lime for Masonry Purposes. https://doi.org/10.1520/C0207-18

C11 Committee, n.d. Specification for Application of Portland Cement-Based Plaster. https://doi.org/10.1520/C0926-23A

Cala, A., Caro, S., Lleras, M., Rojas-Agramonte, Y., 2019. Impact of the chemical composition of aggregates on the adhesion quality and durability of asphalt-aggregate systems. Construction and Building Materials 216, 661–672. https://doi.org/10.1016/j.conbuildmat.2019.05.030

Calcium silicate hydrate | chemical compound | Britannica [WWW Document], 2024. URL https://www.britannica.com/science/calcium-silicate-hydrate (accessed 9.10.24).

Carpet Roll (Environmental Product Declaration), 2021. . GreenTag Global Pty Ltd.

CAS Common Chemistry [WWW Document], n.d. URL https://commonchemistry.cas.org/detail?ref=9002-86-2&terms=Polyvinyl%20chloride (accessed 10.4.24).

Cembrit functional boards (Environmental Product Declaration), 2016. . Cembrit Holding A/S.

Chemical composition of basalt aggregate, quartz sand and WFS (in %). [WWW Document], n.d. . ResearchGate. URL https://www.researchgate.net/figure/Chemical-composition-of-basalt-aggregate-quartz-sand-and-WFS-in_tbl4_338618904 (accessed 9.10.24).

Chemical Composition of Basaltic aggregate [3] [WWW Document], n.d. . ResearchGate. URL https://www.researchgate.net/figure/Chemical-Composition-of-Basaltic-aggregate-3_tbl2_342278361 (accessed 9.10.24).

Chemical Properties of Sandstone,Sandstone Chemical Properties,Chemical Composition of Sandstone [WWW Document], n.d. URL https://www.indianstonehouse.com/sandstone-chemical-properties.html (accessed 10.3.24).

Chemical Properties Of Slatestone,Slatestone Chemical Properties,Chemical Composition of Slatestone [WWW Document], n.d. URL https://www.indianstonehouse.com/slatestone-chemical-properties.html (accessed 10.3.24).

ChemicalBook [WWW Document], 2023. . ChemicalBook. URL https://www.chemicalbook.com/ (accessed 9.10.24).

Chen, H., 2014. Biotechnology of lignocellulose: theory and practice. Springer ; Chemical Industry Press, Dordrecht ; New York.

CoorsTek, 2017. Ceramic Marking Ink Safety Data Sheet. Golden, CO.

CPID [WWW Document], n.d. URL https://www.whatsinproducts.com/types/type_detail/1/12697/standard/span%20style=%22color (accessed 10.3.24).

Crosslam CLT:  EPD for Cross Laminated Timber produced by Structurlam in Okanagan Falls, BC (Environmental Product Declaration), 2020.

D13 Committee, n.d. Specification for Glass Fiber Strands. https://doi.org/10.1520/D0578_D0578M-23

Dana, J.D., 1985. Manual of mineralogy: after James D. Dana, 20. ed. ed. Wiley, New York.

Darling, D., 2005. Generic Latex Paint Products. National Institute of Standards and Technology.

DLW Linoleum Acousticplus Gerford DLW Linoleum Acousticplus Sheet Flooring, 2020. . Gerflor.

DLW Linoleum Compact Gerflor DLW Linoleum Compact Sheet Flooring (Environmental Product Declaration), 2020. . Gerflor.

El-Chabib, H., 2020. Properties of SCC with supplementary cementing materials, in: Self-Compacting Concrete: Materials, Properties and Applications. Elsevier, pp. 283–308. https://doi.org/10.1016/B978-0-12-817369-5.00011-8

Environmental Product Declaration Fibercement Cladding (Environmental Product Declaration), 2019. . James Hardie Building Products Ltd.

EQUITONE [Linea / Lunara] fibre cement sheets (No. EPD-ETE-20190128-CCA1-EN), 2020. . ETEX.

Fakhry, M., Abd El-Kader, M., Al-Abyad, M., El-Shobaki, M., Shouaib, A., 2017. Granite & Marble Industry in Egypt. https://doi.org/10.13140/RG.2.2.12642.12486

Fares, S., Yassene, Ali.A.M., Ashour, A., Abu-Assy, M.K., El-Rahman, M.. A., 2011. Natural radioactivity and the resulting radiation doses in some kinds of commercially marble collected from different quarries and factories in Egypt. NS 03, 895–905. https://doi.org/10.4236/ns.2011.310115

Feldspar - Mineral Composition, Uses, Types | Britannica [WWW Document], 2024. URL https://www.britannica.com/science/feldspar (accessed 10.3.24).

Fereidooni, D., Khajevand, R., 2018. Correlations Between Slake-Durability Index and Engineering Properties of Some Travertine Samples Under Wetting–Drying Cycles. Geotechnical and Geological Engineering 36, 1071–1089. https://doi.org/10.1007/s10706-017-0376-8

Fořt, J., 2015. EFFECT OF SANDSTONE ANISOTROPY ON ITS HEAT AND MOISTURE TRANSPORT PROPERTIES. ms 21, 455–459. https://doi.org/10.5755/j01.ms.21.3.7202

Fuels, N.R.C. (US) S. on P.E.L. for M., 1996. Physical and Chemical Properties of Military Fuels, in: Permissible Exposure Levels for Selected Military Fuel Vapors. National Academies Press (US).

Holcim US, 2022. Hydrated Lime Safety Data Sheet (Safety Data Sheet No. Version 3.1). Chicago, IL.

Home | Reade Advanced Materials [WWW Document], n.d. . Reade. URL https://reade.com/ (accessed 10.3.24).

Industrial Minerals | Slate Powder | Slate Granules | Delabole Slate [WWW Document], n.d. URL http://www.delaboleslate.co.uk/industrial-minerals-spec.asp (accessed 10.3.24).

Industry Average EPD for 5/8” Type X Conventional Gypsum Board, 2020. . Gypsum Association.

Industry-wide cradle-to-gate EPD for 1 /2” and 5 /8” Glass-mat Gypsum Boards, 2021. . Gypsum Association.

Institution, S., n.d. AMYLUM [WWW Document]. Smithsonian Institution. URL https://www.si.edu/object/nmah_993722 (accessed 10.3.24).

Joint Reinforcement for Concrete Masonry, n.d. . Concrete Masonry and Hardscapes Association. URL https://www.masonryandhardscapes.org/resource/tek-12-02b/ (accessed 10.3.24).

JUTE FIBRE, CHEMICAL COMPOSITION OF JUTE FIBRE, CHEMICAL FORMULA OF JUTE FIBRE, n.d. URL https://www.textileadvisor.com/2019/08/jute-fibre-chemical-composition-of-jute.html (accessed 10.3.24).

Kim, S., Chen, J., Cheng, T., Gindulyte, A., He, J., He, S., Li, Q., Shoemaker, B.A., Thiessen, P.A., Yu, B., Zaslavsky, L., Zhang, J., Bolton, E.E., 2023. PubChem 2023 update. Nucleic Acids Research 51, D1373–D1380. https://doi.org/10.1093/nar/gkac956

Kim, S.S., Qudoos, A., Jakhrani, S.H., Lee, J.B., Kim, H.G., 2019a. Influence of Coarse Aggregates and Silica Fume on the Mechanical Properties, Durability, and Microstructure of Concrete. Materials 12, 3324. https://doi.org/10.3390/ma12203324

Kim, S.S., Qudoos, A., Jakhrani, S.H., Lee, J.B., Kim, H.G., 2019b. Influence of Coarse Aggregates and Silica Fume on the Mechanical Properties, Durability, and Microstructure of Concrete. Materials 12, 3324. https://doi.org/10.3390/ma12203324

Knauf Insulation, 2013. Marmox Heraklith Insulation (Safety Data Sheet).

Kocak, Y., 2016. PREDICTING THE EFFECT OF TRASS ON THE COMPRESSIVE STRENGTH OF PORTLAND CEMENT WITH FUZZY LOGIC. Materials, methods & technologies 10, 612–621.

Korkanç, M., Tugrul, A., 2004. Evaluation of selected basalts from Nĭde, Turkey, as source of concrete aggregate. Engineering Geology 75, 291–307. https://doi.org/10.1016/j.enggeo.2004.06.015

Kyzs’michev, A.B., Soloviev, A.V., Gonikberg, V.E., Shapiro, M.N., Zamzhitskii, O.V., 2006. Mesozoic syncollision siliciclastic sediments of the Bols’shoi Lyakhov Island (New Siberian Islands). Stratigr. Geol. Correl. 14, 30–48. https://doi.org/10.1134/S0869593806010035

Large-size fibre cement plates Swisspearl (No. EPD-SWP-20180032-IAD1-EN), 2018. . Swisspearl Group AG.

Lesage, J., Stanley, J., Karoly, W.J., Lichtenberg, F.W., 2007. Airborne Methylene Diphenyl Diisocyanate (MDI) Concentrations Associated with the Application of Polyurethane Spray Foam in Residential Construction. Journal of Occupational and Environmental Hygiene 4, 145–155. https://doi.org/10.1080/15459620601133779

Linseed oil, polymer with maleic anhydride, pentaerythritol and polyethylene glycol ether with glyce - 127970-91-6 | Vulcanchem [WWW Document], n.d. URL https://www.vulcanchem.com/product/main-products/vc475359 (accessed 10.3.24).

Lu, R., Miyakoshi, T., 2015. Chapter 3 - Main compositions of lacquer, in: Lu, R., Miyakoshi, T. (Eds.), Lacquer Chemistry and Applications. Elsevier, Amsterdam, pp. 25–62. https://doi.org/10.1016/B978-0-12-803589-4.00003-1

Makani, A., Vidal, T., Pons, G., Escadeillas, G., 2010. Time-dependent behaviour of high performance concrete: influence of coarse aggregate characteristics. EPJ Web of Conferences 6, 03002. https://doi.org/10.1051/epjconf/20100603002

MARMOLEUM DECIBEL FORBO FLOORING SYSTEMS RESILIENT LINOLEUM FLOOR COVERING (Environmental Product Declaration), 2018. . Forbo Flooring Systems.

Mineral Wool Board (Environmental Product Declaration), 2013. . North American Insulation Manufacturers Association.

Mineral Wool Loose (Environmental Product Declaration), 2013. . North American Insulation Manufacturers Association.

Moazenian, A., 2017. Experimental Study on the Use of Trass as a Supplementary Cementitious Material in Pervious Concrete. Environmental Science and Engineering.

Moreno-Pérez, E., Hernández-Ávila, J., Rangel-Martínez, Y., Cerecedo-Sáenz, E., Arenas-Flores, A., Reyes-Valderrama, Ma.I., Salinas-Rodríguez, E., 2018. Chemical and Mineralogical Characterization of Recycled Aggregates from Construction and Demolition Waste from Mexico City. Minerals 8, 237. https://doi.org/10.3390/min8060237

Mubiayi, M.P., 2014. Mineralogical and Physical Characterisation of QwaQwa Sandstones, in: Yang, G.-C., Ao, S.-I., Gelman, L. (Eds.), Transactions on Engineering Technologies. Springer Netherlands, Dordrecht, pp. 213–225. https://doi.org/10.1007/978-94-017-8832-8_16

NATURA, TEXTURA and MATERIA Fiber-Cement Panels (No. EPD-ELH-20180136-CAC1-EN), n.d. . ETEX.

Nimfopoulos, M., Hadjispyrou, S., Polya, D., Michailidis, K.M., Trontsios, G., 2002. Geochemical conditions and environmental pollution from hydrothermal waters of the Anthemous basin, Thessaloniki, N. Greece. https://doi.org/10.13140/RG.2.1.2888.0407

North America Ceramic Tile: Porcelain, Pressed Floor, Mosaic Quarry, Glazed Wall (Environmental Product Declaration), 2020. . Industry-wide.

North American Glue Laminated Timber (Environmental Product Declaration), 2020. . American Wood Council, Canadian Wood Council.

North American Oriented Strand Board (Environmental Product Declaration), 2020. . American Wood Council, Canadian Wood Council.

North American Softwood Lumber (Environmental Product Declaration), 2020. . American Wood Council, Canadian Wood Council.

North American Softwood Plywood (Environmental Product Declaration), 2020. . American Wood Council, Canadian Wood Council.

Nylon 6 vs. nylon 6/6 [WWW Document], n.d. URL https://www.essentracomponents.com/en-us/news/solutions/fastening-components/the-differences-between-nylon-6-and-nylon-6-6 (accessed 10.4.24).

Petersen, J.C., n.d. Chemical Composition of Asphalt as Related to Asphalt Durability: State of the Art. Transportation Research Record.

Pigments through the Ages - Technical Information - Umber [WWW Document], n.d. URL https://www.webexhibits.org/pigments/indiv/technical/umber.html (accessed 9.10.24).

Polymeric Geomembrane Components in Landfill Liners - ScienceDirect [WWW Document], n.d. URL https://www.sciencedirect.com/science/article/pii/B9780124077218000176 (accessed 10.3.24a).

Polymeric Geomembrane Components in Landfill Liners - ScienceDirect [WWW Document], n.d. URL https://www.sciencedirect.com/science/article/abs/pii/B9780124077218000176 (accessed 10.3.24b).

Polyols | Polyester Polyols | Supplier & Distributor, 2019. . Arpadis. URL https://www.arpadis.com/polyester-polyols/ (accessed 10.3.24).

POLYVINYL ALCOHOL | CAMEO Chemicals | NOAA [WWW Document], n.d. URL https://cameochemicals.noaa.gov/chemical/7523 (accessed 10.4.24).

Properties of inorganic polymer (geopolymer) mortars made of glass cullet | Journal of Materials Science [WWW Document], n.d. URL https://link.springer.com/article/10.1007/s10853-011-6107-2 (accessed 10.3.24).

Puettmann, M.E., Wilson, J.B., n.d. GATE-TO-GATE LIFE-CYCLE INVENTORY OF GLUED-LAMINATED TIMBERS PRODUCTION.

Raclavská, H., Kantor, P., Růžičková, J., Kucbel, M., Švédová, B., Slamová, K., Flodrová, Š., Juchelková, D., 2021a. An Influence of the Fuel Type on Element Behaviour in Domestic Boilers with Respect to the Circular Economy. Applied Sciences 11, 4980. https://doi.org/10.3390/app11114980

Raclavská, H., Kantor, P., Růžičková, J., Kucbel, M., Švédová, B., Slamová, K., Flodrová, Š., Juchelková, D., 2021b. An Influence of the Fuel Type on Element Behaviour in Domestic Boilers with Respect to the Circular Economy. Applied Sciences 11, 4980. https://doi.org/10.3390/app11114980

Research, C.M., 2023. Polyolefin Film Market is expected to grow at a CAGR of 4.9% within [WWW Document]. URL https://www.openpr.com/news/2916818/polyolefin-film-market-is-expected-to-grow-at-a-cagr-of-4-9-within (accessed 10.3.24).

Risdanareni, P., Ekaputri, J.J., Triwulan, 2014. The Influence of Alkali Activator Concentration to Mechanical Properties of Geopolymer Concrete with Trass as a Filler. MSF 803, 125–134. https://doi.org/10.4028/www.scientific.net/MSF.803.125

Rodriguez Alonso, E., 2015. Contribution to the study of formation mechanisms of condensable by-products from torrefaction of various biomasses (PhD Thesis).

Rodriguez, M.A., Rubio, J., Rubio, F., Liso, M.J., Oteo, J.L., 1997. Application of Inverse Gas Chromatography to the Study of the Surface Properties of Slates. Clays and clay miner. 45, 670–680. https://doi.org/10.1346/CCMN.1997.0450506

Rowell, R., Pettersen, R., Tshabalala, M., 2012. Cell Wall Chemistry, in: Handbook of Wood Chemistry and Wood Composites, Second Edition. CRC Press, pp. 33–72. https://doi.org/10.1201/b12487-5

Rowell, R.M. (Ed.), 2012. Handbook of Wood Chemistry and Wood Composites, 0 ed. CRC Press. https://doi.org/10.1201/b12487

Rwahwire, S., Tomkova, B., Periyasamy, A.P., Kale, B.M., 2019. Chapter 3 - Green thermoset reinforced biocomposites, in: Koronis, G., Silva, A. (Eds.), Green Composites for Automotive Applications, Woodhead Publishing Series in Composites Science and Engineering. Woodhead Publishing, pp. 61–80. https://doi.org/10.1016/B978-0-08-102177-4.00003-3

Sánchez-Cotte, E.H., Pacheco-Bustos, C.A., Fonseca, A., Triana, Y.P., Mercado, R., Yepes-Martínez, J., Lagares Espinoza, R.G., 2020. The Chemical-Mineralogical Characterization of Recycled Concrete Aggregates from Different Sources and Their Potential Reactions in Asphalt Mixtures. Materials 13, 5592. https://doi.org/10.3390/ma13245592

Sariisik, G., 2012. Determining Performance of Marble Finished Products on Their Usage Areas by a New Impact-Resistance Test Method. Journal of Testing and Evaluation 40, 1–7. https://doi.org/10.1520/JTE104352

Savoly, A., Elko, D., 2015. Gypsum Wallboard Chemical Additives. Geo Specialty Chemicals.

SBS mixture [WWW Document], n.d. URL https://www.chembk.com/en/chem/SBS%20mixture (accessed 10.3.24).

SBS-MODIFIED BITUMEN ROOFING MEMBRANE INSTALLATION: TORCH APPLIED (Environmental Product Declaration), 2018. . Asphalt Roofing Manufacturers Association.

See our Glass Cullet properties [WWW Document], n.d. URL https://www.allglass.uk/products/glass-cullet-properties/ (accessed 10.3.24).

Shivokevich, P., Weinberg, L.M., 2021. Distilled Tall Oil (Technical Evaluation Report), Crops. U.S. Department of Agriculture.

Siddique, R., Cachim, P. (Eds.), 2018. Waste and supplementary cementitious materials in concrete: characterisation, properties, and applications, Woodhead Publishing Series in civil and structural engineering. Woodhead Publishing, Duxford [England] ; Cambridge, MA.

Singh Jadaun, J., Bansal, S., Sonthalia, A., Rai, A.K., Singh, S.P., 2022. Biodegradation of plastics for sustainable environment. Bioresource Technology 347, 126697. https://doi.org/10.1016/j.biortech.2022.126697

Soda-lime glass | All About Glass [WWW Document], n.d. URL https://allaboutglass.cmog.org/definition/soda-lime-glass (accessed 10.3.24).

Solvent naphtha (petroleum), light arom. [WWW Document], n.d. URL https://www.chembk.com/en/chem/Solvent%20naphtha%20(petroleum),%20light%20arom. (accessed 10.3.24).

Spray polyurethane foam insulation (HFO/HFC) (Environmental Product Declaration), 2022. . Spray Polyurethane Foam Alliance.

Standard Reference Material 1d Limestone, Argillaceous (Certificate of Analysis No. SRM 1d), 2005. . National Institute of Standards and Technology (NIST).

Standard Reference Material 69b Bauxite (Arkansas) (Certificate of Analysis No. SRM 2690), 1991. . National Institute of Standards and Technology (NIST).

Standard Reference Material 679 Brick Clay (Certificate of Analysis No. SRM 679), 1987. . National Institute of Standards and Technology (NIST).

Standard Reference Material 1885b Portland Cement (Certificate of Analysis No. SRM 1885b), 2013. . National Institute of Standards and Technology (NIST).

Standard Reference Material 2690 Coal Fly Ash (Certificate of Analysis No. SRM 2690), 2015. . National Institute of Standards and Technology (NIST).

Standard Reference Material 2696 Silica Fume (powder form) (Certificate of Analysis No. SRM 2696), 2017. . National Institute of Standards and Technology (NIST).

Stoddard Solvent, Thermo Scientific Chemicals, Quantity: 1 kg | Fisher Scientific [WWW Document], n.d. URL https://www.fishersci.com/shop/products/stoddard-solvent-thermo-scientific/AC419730050#?keyword= (accessed 10.3.24).

Tacharanite Mineral Data [WWW Document], n.d. URL https://webmineral.com/data/Tacharanite.shtml# (accessed 10.4.24).

Taylor, P., Wilson, E., Ceylan, H., 2012. Concrete Pavement Mixtures Design and Analysis: The Application of Portable X-Ray Fluorescence Technique to Assess Concrete Mix Proportions.

Techno File: Glaze Lab [WWW Document], n.d. . Default. URL https://ceramicartsnetwork.org/ceramics-monthly/ceramics-monthly-article/techno-file-glaze-lab (accessed 9.10.24).

The Athena Sustainable Materials Institute, n.d. A Cradle-to-Gate Life Cycle Assessment of Ready-Mixed Concrete Manufactured by NRMCA Members – Version 3. National Ready Mixed Concrete Association (NRMCA).

The chemistry of polyurethanes [WWW Document], n.d. URL https://www.l-i.co.uk/knowledge-centre/the-chemistry-of-polyurethanes/ (accessed 10.3.24).

The Chemistry, Physics and Manufacturing of Glaze Frits [WWW Document], n.d. URL https://digitalfire.com/article/the+chemistry%2C+physics+and+manufacturing+of+glaze+frits (accessed 9.10.24).

Tris (chloroisopropyl) phosphate (TCPP) - The Chemical Company [WWW Document], n.d. URL https://thechemco.com/chemical/tris-chloroisopropyl-phosphate-tcpp/ (accessed 10.3.24).

Tufail, M., Shahzada, K., Gencturk, B., Wei, J., 2017a. Effect of Elevated Temperature on Mechanical Properties of Limestone, Quartzite and Granite Concrete. Int J Concr Struct Mater 11, 17–28. https://doi.org/10.1007/s40069-016-0175-2

Tufail, M., Shahzada, K., Gencturk, B., Wei, J., 2017b. Effect of Elevated Temperature on Mechanical Properties of Limestone, Quartzite and Granite Concrete. Int J Concr Struct Mater 11, 17–28. https://doi.org/10.1007/s40069-016-0175-2

Tufted broadloom carpet - luxury class LC1-LC5 - with 1200 g/m2 maximum surface pile weight - pile material made of polyamide 6, textile backing (Environmental Product Declaration No. EPD-GUT-20160117-CCA1-EN), 2016. . Gemeinschaft umweltfreundlicher Teppichboden e.V.

Tufted carpet tiles - luxury class LC1-LC5 - with 1200 g/m2 maximum surface pile weight - pile material made of polyamide 6.6, bitumen based heavy backing (Environmental Product Declaration No. EPD-GUT-20160019-CCA1-EN), 2016. . Gemeinschaft umweltfreundlicher Teppichboden e.V.

United States Gypsum Company, 2023. SHEETROCK® Brand All Purpose Joint Compound, Ready-Mixed (Safety Data Sheet No. 918344).

United States Gypsum Company, 2014. SHEETROCK® Brand All Purpose Joint Compound, Ready-Mixed.

United States Gypsum Conmpany, 2003. USG SHEETROCK® Brand Joint Tape (Safety Data Sheet).

U.S. Environmental Protection Agency, 2016. AP-42: Compilation of Air Emissions Factors from Stationary Sources. U.S. Environmental Protection Agency.

Van der Vegt, A.K., Govaert, L.E., 2005. Polymeren: van keten tot kunststof. VSSD, Delft.

Wagners CFT Manufacturing Pty Ltd, n.d. FRP structural pultrusion technical specifications composite fiber technologies.

WHAT'S THAT STUFF? - Asphalt [WWW Document], n.d. URL https://pubsapp.acs.org/cen/whatstuff/stuff/7747scit6.html (accessed 10.3.24).

Wintsch, R.P., Kvale, C.M., Kisch, H.J., 1991. Open-system, constant-volume development of slaty cleavage, and strain-induced replacement reactions in the Martinsburg Formation, Lehigh Gap, Pennsylvania. Geological Society of America Bulletin 103, 916–927. https://doi.org/10.1130/0016-7606(1991)103<0916:OSCVDO>2.3.CO;2

Yuan, Q., Liu, Z., Zheng, K., Ma, C., 2021. Chapter 5 - Wood, in: Yuan, Q., Liu, Z., Zheng, K., Ma, C. (Eds.), Civil Engineering Materials. Elsevier, pp. 239–259. https://doi.org/10.1016/B978-0-12-822865-4.00005-2

Zumrawi, M., Abdalla, E., 2018. STABILIZATION OF EXPANSIVE SOIL USING MARBLE WASTE POWDER .
